# *Streptomyces* sesquiterpenes elicit 10-HCA secretion and recruit disease-suppressive microbiota to enhance banana *Fusarium* wilt resistance

**DOI:** 10.1038/s41467-026-73928-x

**Published:** 2026-06-03

**Authors:** Yufeng Chen, Junting Feng, Peitao Lü, Dengbo Zhou, Yongzan Wei, Tao Jing, Zai Zheng, Waseem Raza, Dengfeng Qi, Miaoyi Zhang, Yankun Zhao, Kai Li, Wei Wang, Xu Cheng, Jianghui Xie

**Affiliations:** 1 State Key Laboratory of Tropical Crop Breeding, Institute of Tropical Bioscience and Biotechnology & Sanya Research Institute, Chinese Academy of Tropical Agricultural Sciences, Haikou, China; 2 Shenzhen Branch, Guangdong Laboratory of Lingnan Modern Agriculture, Key Laboratory of Synthetic Biology, Ministry of Agriculture and Rural Affairs, Agricultural Genomics Institute at Shenzhen, Chinese Academy of Agricultural Sciences, Shenzhen, China

**Keywords:** Microbiome, Soil microbiology, Environmental biotechnology

## Abstract

Plant-beneficial microbe interactions are vital for enhancing soil-borne disease resistance, largely through the assembly of a disease-suppressive microbiome. However, the mechanisms governing these interactions remain elusive. Here, we establish an interaction model between banana and *Streptomyces yongxingensis* sp. nov. 2-11. We demonstrate that strain Sy2-11 suppresses banana *Fusarium* wilt (BFW) by recruiting a protective rhizosphere microbiome. Furthermore, we identify sesquiterpenes (aristolene and ledene), produced by strain Sy2-11, as key signaling molecules that trigger banana roots to biosynthesize 10-hydroxycapric acid (10-HCA). Interestingly, 10-HCA specifically enriches beneficial *Bacillus* spp., which is essential for the suppression of BFW. This effect is validated by synthetic communities (SynComs) and chemotaxis-deficient mutants of *Bacillus velezensis*. Our findings reveal a previously unreported mechanism that differs from conventional plant-microbe interactions, whereby *Streptomyces*, acting as a beneficial elicitor, releases sesquiterpene signals to trigger 10-HCA secretion in banana plants, thereby orchestrating the assembly of a rhizosphere microbiome that suppresses BFW. These findings provide a promising strategy for rhizosphere micro-ecological regulation and sustainable soil-borne disease control, with significant potential for advancing sustainable agriculture.

## Abstract (web summary)

*Streptomyces* acts as a beneficial elicitor and releases sesquiterpene signals to trigger 10-HCA secretion in banana plants, thereby orchestrating the assembly of rhizosphere microbiome that suppresses banana Fusarium wilt.

## Introduction

Soil-borne plant diseases pose a significant threat to global food security by severely impacting crop health and yield, causing substantial economic losses worldwide^1,2^. Among these, *Fusarium* wilt, caused by *Fusarium oxysporum*, is particularly devastating affecting over a hundred species of essential fruit and staple crops^3–6^. Bananas, a staple food for approximately 500 million people, are highly susceptible to banana *Fusarium* wilt (BFW), caused by *Fusarium oxysporum* f. sp. *cubense* (*Foc*)^7^. This disease, often referred to as the “cancer” of bananas, is especially virulent in its tropical race 4 strain (*Foc* TR4), which can infect nearly all banana cultivars, and its spores remain viable in the soil for over 30 years, making long-term control extremely challenging. The disease progresses from asymptomatic infiltration into the host roots to colonization of the xylem vessels and further spread into the aboveground tissues, ultimately causing wilt and plant death^6^. Furthermore, mycotoxins secreted by the fungus can diminish the quality of agricultural products and pose potential health risks to humans^6,8^. Therefore, controlling BFW has become a global concern^9^. Conventional chemical strategies have proven ineffective and unsustainable, threatening human health and incurring significant environmental costs^6,10^. Biocontrol agents are thus proposed as environmentally friendly alternatives to chemical pesticides in agricultural systems^4^. Developing novel and environmentally friendly approaches to reduce BFW incidence and prevent crop yield loss is essential.

The rhizosphere microbiome plays a crucial role in regulating plant health, as numerous studies have shown that the plant microbiome can inhibit pathogen invasion and mitigate the outbreak of soil-borne diseases^4,11–13^. Plant roots serve as the epicenter for interactions among plants, pathogens, and the rhizosphere microbial community. Under pathogens attack, plants can selectively recruit specific beneficial microbes to form a disease-suppressive rhizosphere microbiome, providing consistent and long-lasting protection^14–19^. However, the high virulence of pathogens such as *Foc* TR4 makes them unsuitable as elicitors to trigger the “cry for help” response^13,20,21^. Therefore, identifying proper elicitors is a sustainable strategy to explore the natural defense mechanisms provided by the plant’s “cry for help” response.

*Streptomyces*, a genus of filamentous bacteria, are renowned to produce broad-spectrum bioactive metabolites and their role in controlling plant diseases^22,23^. These soil-rich bacteria typically exhibit slow growth rates and have evolved specific survival strategies within plant and soil habitats^24^. *Streptomyces* spp. can establish extensive networks covering plant roots, occupying ecological niches and subsequently enhancing the plants’ resilience to environmental stresses^24,25^. Additionally, numerous *Streptomyces* spp. possess structures capable of penetrating plant cells, aiding their colonization of plant surface and interior tissues^26^. This makes them a promising candidate for sustainable agriculture. The value of *Streptomyces* in agriculture and biological control cannot be underestimated. The most studied strategies for controlling plant diseases by *Streptomyces* include antibiotic synthesis, iron competition, inducing plant systemic resistance, and regulation of defense-related metabolism^27^. However, the role of *Streptomyces* acts as an elicitor in prompting plants to initiate a “cry for help” response remains poorly understood.

In this study, we characterize the antagonistic potential and underlying mechanisms of *Streptomyces yongxingensis* sp. nov. 2-11 (Sy2-11) strain against BFW. We further established a tri-trophic interaction model system consisting of strain Sy2-11, *Foc* TR4, and banana plants. Our objectives are to: (1) assess the impact of strain Sy2-11 on banana growth and the suppression of *Foc* TR4; (2) investigate the modulation of the rhizosphere microbiome during disease suppression and identify key beneficial microbiome members; (3) examine the metabolic profile changes in banana root exudates induced by *Streptomyces* Sy2-11 strain and identify key metabolites responsible for recruiting a core suppressive microbiome; (4) exploring the pathways through which strain Sy2-11 alters banana root metabolites; (5) use synthetic microbial communities (SynComs) and plant-derived metabolites to validate the role of beneficial microbiome members in disease suppression; (6) construction of motility-related genes mutants in *B. velezensis* members and analysis of their chemotaxis toward 10-HCA; and (7) propose a comprehensive model for rhizosphere microbiome interactions driven by strain Sy2-11 during *Foc* TR4 suppression in banana.

## Results

### *S. yongxingensis* sp. nov. 2-11 strain controls the banana *Fusarium* wilt under natural soil conditions

In our previous study, a strain*, S. yongxingensis* sp. nov. 2-11, was newly identified from the soft coral *Menella woodin*^10^. The type strain Sy2-11^T^ ( = GDMCC 4.213 ^T^ = JCM 34965 ^T^) is a gram-positive, aerobic, and non-motile actinobacteria forming branched substrate and aerial mycelia, which differentiate into short and spiral spore chains. Strain Sy2-11 was highly effective in controlling BFW^10^. To investigate underlying mechanisms by which the strain Sy2-11 controls the BFW, we established a generic assay by inoculating strain Sy2-11 in sterile soil and natural soil to assess its impact on BFW incidence. In natural soil, after 35 days of inoculation, we observed that the disease incidence of banana seedlings inoculated with strain Sy2-11 was only 13.33%, representing a 60% reduction compared to the control (Tukey’s HSD test, *p*  <  0.05; Supplementary Fig. 1). However, when the soil was sterilized, the disease incidence of banana seedlings inoculated with strain Sy2-11 was 66.67%, which was 13% lower than that of the control group (Tukey’s HSD test, *p*  <  0.05; Supplementary Fig. 1). Quantitative analysis showed that after inoculation with strain Sy2-11, the pathogen density was decreased by 44.93 times in natural soil compared to the control. When the natural soil was sterilized, the activity of strain Sy2-11 significantly declined by more than 10 times. These results showed that strain Sy2-11 significantly reduced BFW incidence in the presence of the rhizosphere microbiome.

To determine the ability of strain Sy2-11 to colonize the banana rhizoplane and endorhiza, strain Sy2-11 was labeled with a GFP marker to investigate its competence to colonize banana roots using confocal laser scanning microscopy (CLSM). Abundant GFP-Sy2-11 mycelia were observed on the root surface at 3, 7, and 14 days post-inoculation (dpi) in the PST group (Supplementary Fig. 2). In contrast, no GFP‑Sy2‑11 fluorescence was detected inside root tissues, indicating that the strain colonizes the rhizosphere effectively, and has the potential to establish long-term associations with banana plants. Similarly, another trial result of ampicillin‑resistant marker of strain Sy2‑11 and its colonization showed that strain Sy2-11 can effectively colonize in rhizosphere soil and rhizoplane at 3, 7, and 14 dpi, respectively. However, no strain Sy2‑11 was detected within banana endorhiza (Supplementary Fig. 3).

**Fig. 1:**
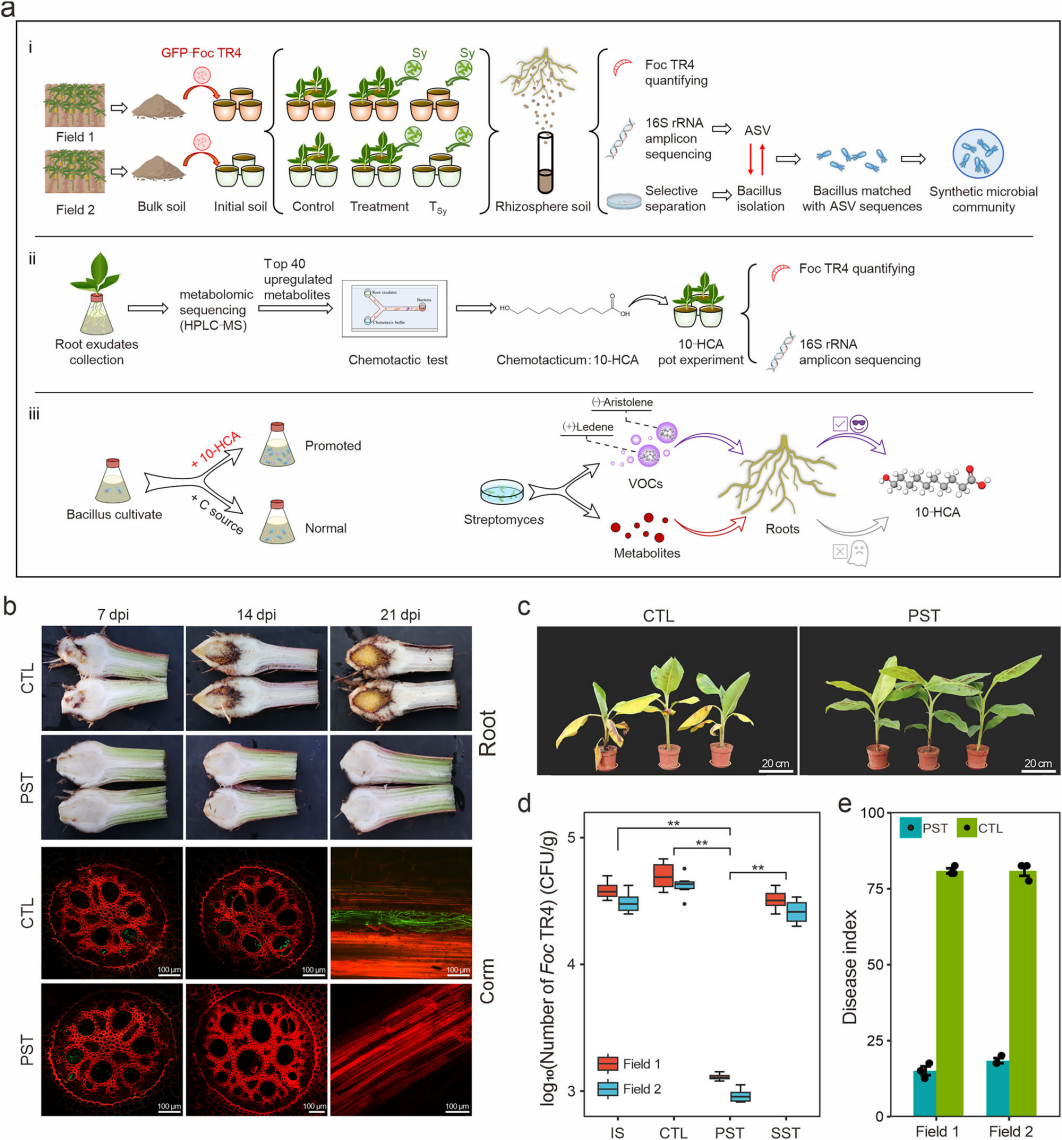
*S. yongxingensis* sp. nov. Sy2-11-induced banana rhizosphere microbiota recruitment and *Fusarium* wilt disease resistance. **a** An overview of the experimental workflow for this study. **b** Cross-sectional observations of banana corms and roots following inoculation with the *Foc* TR4 in Field 1 (*n* = 3). Green: *Foc* TR4, red: the background color of plants. **c** Phenotypic comparison of banana seedlings in the control and treatment groups at 35 dpi. While the treatment group showed robust health, the control group exhibited distinct symptoms of *Fusarium* wilt in Field 2. **d** A comparison of *Foc* TR4 content in the PST group versus the IS, CTL, and SST groups, demonstrating a significantly reduced pathogen load in the PST group. Horizontal bars within boxes represent the median. The tops and bottoms of boxes represent 75th and 25th quartiles, respectively. The upper and lower whiskers represent the range of non-outlier data values. Statistical significance was assessed using two-sided Student’s *t*test, with ‘**’ indicating *P*  <  0.01 (*n* = 6). **e** Disease index analysis revealed a significantly lower disease incidence in the PST group compared to the CTL group across both orchard soils. Data are presented as mean values ± SEM (*n* = 3). In (**b**–**e**), IS: initial soil; CTL: control treatment consisted of plants treated with sterilized water; PST: plants inoculated with strain Sy2-11; SST: soil (without plants) inoculated with strain Sy2-11.

To elucidate the infection dynamics of *Foc* TR4 and the disease resistance mechanisms conferred by strain Sy2-11, soil samples were collected from two banana plantations with a history of monoculture and high BFW incidence. Greenhouse experimental systems, with and without plants, were established and inoculated with GFP-labeled *Foc* TR4 and strain Sy2-11 (Fig. 1a). The dynamic infection process of GFP-*Foc* TR4 was subsequently tracked at various time points. At 7 dpi, GFP-*Foc* TR4 hyphae and spores were observed adhering to root epidermal cells in the control group (Fig. 1b). The hyphae penetrated the epidermal cells and moved along the root vascular bundles. By 14 dpi, GFP-*Foc* TR4 had colonized the banana stems, producing a proliferation of spores and mycelia (Fig. 1b). At 21 dpi, hyphae spread longitudinally through intercellular spaces and entered the xylem of the vascular bundle, a phenomenon not observed in the strain Sy2-11 treated group (Fig. 1b). Cross-sectional analyses of banana corms revealed black spots in the control group at 7 dpi, which progressed to complete browning over time. In contrast, corms treated with strain Sy2-11 remained healthy, devoid of black spots (Fig. 1b). By 35 dpi, the control group exhibited complete leaf wilting and extensive corm rot, whereas the strain Sy2-11-treated group remained vigorous (Fig. 1c**;** Supplementary Fig. 4). Quantitative analysis of *Foc* TR4 in the soil showed significantly lower densities in the strain Sy2-11 treated group compared to other groups (Tukey’s HSD test, *p*  <  0.05; Fig. 1d). Evaluation of disease severity in plants grown in two types of soil revealed an average disease index of 80% in the control group, compared to 17.5% in the strain Sy2-11 treated group (Fig. 1e). Additionally, strain Sy2-11 significantly enhanced plant performance, as evidenced by increased plant height, fresh and dry weights, pseudo-stem girth, leaf area, and chlorophyll content (Supplementary Fig. 5). Furthermore, to evaluate the effect of inoculum dosage of strain Sy2‑11 on the disease index, we performed disease resistance assays with a range of strain Sy2-11 concentrations. Compared with the control group, which exhibited a disease index of 79.17%, inoculation with strain Sy2-11 at concentrations of 10^7^, 10^6^, and 10^5^ CFU/g soil significantly reduced the disease indices to 18.06%, 19.44%, and 20.83%, respectively (Supplementary Fig. 6). Even at a low inoculation concentration (10^4^, 10^3^ CFU/g soil), strain Sy2-11 conferred a significantly lower disease index than other *Streptomyces* strains (5-6 and H7) inoculated at higher concentrations (10^6^ CFU/g soil, Supplementary Fig. 6).

### *S. yongxingensis* sp. nov. strain Sy2-11 induces *Bacillus* spp. recruitment in banana rhizosphere

**Fig. 2:**
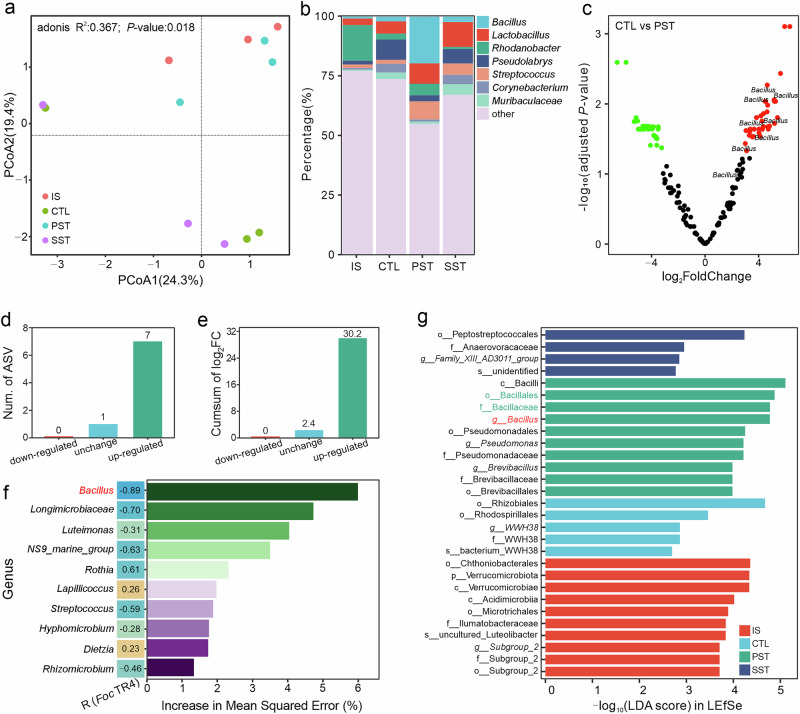
*Bacillus* as the core microbiota recruited by strain Sy2-11-induced banana roots in Field 1. **a** The impact of strain Sy2-11 on the *β*-diversity of bacterial communities in the banana rhizosphere. The R² and *P* value were derived from a PERMANOVA (permutational multivariate analysis of variance) test. **b** Bar plot displaying the relative abundance of Amplicon Sequence Variants (ASVs) at the genus level across the four experimental groups, highlighting the top seven genera. **c** Volcano plot showing a significant increase in *Bacillus* abundance in the PST group compared to the CTL group. Differentially abundant ASVs were identified using DESeq2, and *P*-values were corrected using the Benjamini-Hochberg method. Detailed adjusted *P*-values are provided in Supplementary Data 1. **d** The number of *Bacillus* species that were upregulated and downregulated in response to strain Sy2-11 treatment. **e** Cumulative log_2_ (Fold Change) plot of *Bacillus*. **f** Random forest analysis displaying the top 10 important genera, with a heatmap on the left illustrating the Pearson correlation coefficients between these genera and *Foc* TR4. **g** LEfSe analysis confirming *Bacillus* as the most significant biomarker in the PST group. For LEfSe analysis, the LDA score measures the discriminant effect size for group differentiation, with higher values indicating a greater contribution to intergroup differences. IS: initial soil; CTL: control treatment consisted of plants treated with sterilized water; PST: plants inoculated with strain Sy2-11; SST: soil (without plants) inoculated with strain Sy2-11.

Next, we investigated the impact of strain Sy2-11 on the assembly of the banana rhizosphere microbiome. Our results indicate that the rhizosphere bacterial communities from Field 1 and 2 exhibited similar *α*-diversity, with no significant differences between treatments (Supplementary Fig. 7). Principal coordinate analysis (PCoA) based on Bray-Curtis dissimilarities revealed that the strain Sy2-11 inoculation had the greatest effect on the microbiome composition, with the strain Sy2-11 treated group (PST) distinctly separated from the control (CTL) and soil (without plants) inoculated with strain Sy2-11 (SST) groups along PCoA1 and PCoA2 (PERMANOVA test; R^2^  =  0.367, *P*  =  0.018; Fig. 2a). Overall, inoculation of strain Sy2-11 to banana significantly affected the composition of the rhizosphere bacterial community.

Genus distribution analysis showed that *Bacillus* was the most abundant and significantly enriched in the rhizosphere of banana plants inoculated with strain Sy2-11, compared with 79.9% of the community, which was considerably higher than in the initial soil (IS), CTL, and SST groups (Fig. 2b). This suggests that *Bacillus* may be a core microbiota associated with disease resistance (Detected in more than 70% of samples, with relative abundance ranked in the top 1%). Differential abundance analysis ( | log_2_FC | ≥ 2, *P* <0.05) confirmed significant enrichment of *Bacillus* in the strain Sy2-11 treated group compared to the control and SST groups (Fig. 2c). Among the eight *Bacillus* species detected, seven were enriched in the strain Sy2-11 treated group, with a significant accumulated fold change of 30.2 (Fig. 2d, e). Additionally, random forest analysis between *Foc* TR4 and Amplicon Sequence Variants (ASVs) showed a significantly negative relationship between *Bacillus* ASVs and *Foc* TR4, with a Pearson correlation coefficient of -0.89 (*P*  <  0.05; Fig. 2f). LEfSe analysis further identified *Bacillus* as a biomarker for the strain Sy2-11 treated group, with an LDA value of 4.78 (Fig. 2g). Similar results were observed in the Field 2 soil system, where *Bacillus* was significantly enriched and served as a biomarker in the strain Sy2-11 treated group (Supplementary Fig. 8-9). These results indicate that strain Sy2-11 remodels the assembly of the banana rhizosphere bacterial community and specifically drives marked enrichment of *Bacillus*. This genus exhibits a significant negative correlation with *Foc* TR4 and constitutes a key biomarker tightly associated with disease resistance.

### *Bacillus* displays clustering centrality in *Streptomyces*-induced banana rhizosphere

**Fig. 3:**
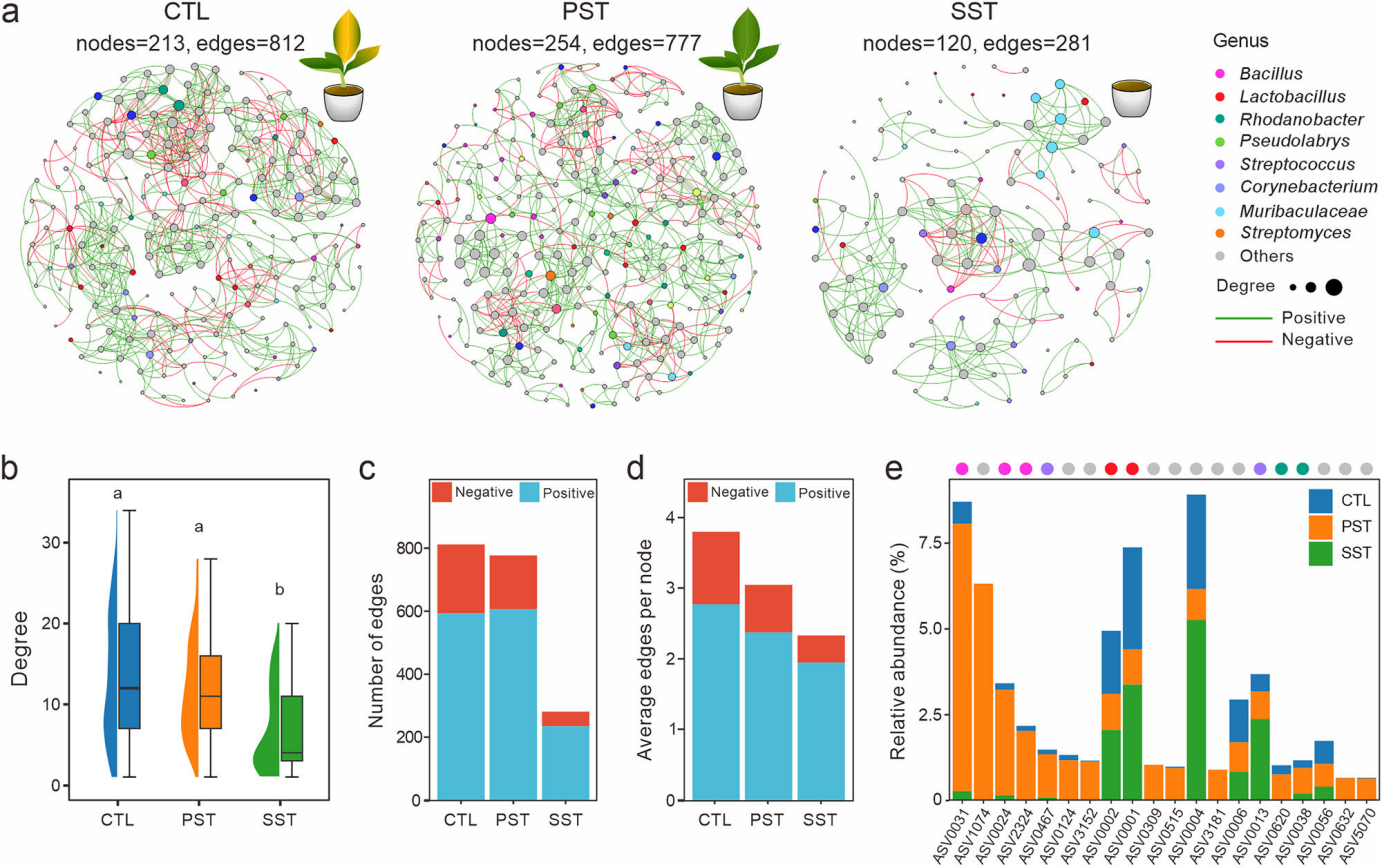
*Bacillus* is a crucial component of microbial networks. **a** The microbial co-occurrence network topology, demonstrating that *Bacillus* occupies a central position in the PST group, in contrast to its placement in the CTL and SST groups. Data represent *n* = 3 biologically independent samples. **b** The network degree for both the PST and CTL groups is significantly higher than that of the SST group. Statistical significance was assessed using LSD multiple comparison tests, with different letters indicating significant differences (*P* < 0.05, n = 213, 254, and 120 for CTL, PST and SST, respectively). Horizontal bars within boxes represent the median. The tops and bottoms of boxes represent 75th and 25th quartiles, respectively. The upper and lower whiskers represent the range of non-outlier data values. **c** The number of network positive edges in the PST group is greater than in the CTL and SST groups. **d** The average number of edges per node in the PST and CTL groups is higher than in the SST group. **e** ASVs wer**e** ranked by relative abundance. ASVs of *Bacillus* exhibited high abundance and centrality in the PST group network, highlighting only the top seven genera. Identical colors represent the same genus in (**a**) and (**e**), with pink indicating *Bacillus*. CTL: control treatment consisted of plants treated with sterilized water; PST: plants inoculated with strain Sy2-11; SST: soil (without plants) inoculated with strain Sy2-11.

The stability of the microbial community is crucial for maintaining functional microbiomes. To elucidate the impact of strain Sy2-11 on the co-occurrence patterns within the banana rhizosphere microbiome, we constructed co-occurrence networks. Our analysis revealed differences in microbial diversity, community composition, and network structure among different treatment groups (Fig. 3a; Supplementary Fig. 10). The results revealed that the IS network comprised multiple highly clustered core modules, characterized by dense within-module connectivity but very few between-module links. The numbers of nodes and edges were substantially higher than those in the CTL, PST, and SST groups (Supplementary Fig. 10). In IS and SST groups, *Muribaculaceae* was the dominant core genus and occupied most of the major modules within the network. By contrast, the PST and CTL groups exhibited significantly higher numbers of degrees, nodes, and edges than the SST group, indicating that taxa in the CTL and PST groups formed more interaction links (Fig. 3b, c). Compared with the CTL group, the PST group contained more nodes but relatively fewer edges, along with a higher proportion of positive interactions (Fig. 3a–d). These network matrices suggest that, in the presence of banana seedlings, the pathogen established a relatively stable pathogenic microbial community, whereas the introduction of strain Sy2-11 disrupted this community, reduced network complexity and stability, and thereby enhanced soil disease resistance.

Among the enriched hub taxa, *Bacillus* emerged as the top-ranking taxon, followed by *Lactobacillus*, *Rhodanobacter*, *Pseudolabrys*, *Streptococcus*, *Corynebacterium*, *Muribaculaceae*, and *Streptomyces*. (Fig. 3a). *Bacillus* ASVs displayed higher relative abundance and centrality in the strain Sy2-11 treated group. Specifically, ASV0031, ASV0024, and ASV2324, belonging to *Bacillus*, exhibited significantly higher abundance (Fig. 3e). These results indicate that *Bacillus* is a key hub taxon in the strain Sy2-11 treated group network, playing a crucial role in regulating the entire microbial community. Additionally, *Bacillus* and *Streptomyces*, which were enriched in the strain Sy2-11 treated group, were identified as top hub taxa. A positive regulatory relationship between *Streptomyces* and *Bacillus* was observed, contributing to enhanced network stability.

### Isolation of *Bacillus* strains from core ASVs and their antagonistic activity against *Foc* TR4

To determine whether the highly enriched *Bacillus* taxon contributes to disease suppression, we conducted specific isolation protocols to isolate *Bacillus* strains from the rhizosphere soil of the strain Sy2-11 treated group. A total of 260 *Bacillus* strains were isolated and characterized (Supplementary Fig. 11**;** Supplementary Data 2). We then evaluated the disease-suppressing capabilities of 260 *Bacillus* strains through antagonism assays. The isolated *Bacillus* strains exhibited significantly higher inhibition rates against *Foc* TR4 (average inhibition rate of 62.18%) (Supplementary Fig. 11). Notably, 95.6% of the *Bacillus* isolates demonstrated disease suppression effects (Supplementary Fig. 12**;** Supplementary Data 2). To assess the correspondence between isolates and the core ASVs, we correlated the isolate sequences with ASVs based on 100% sequence similarity. A total of 103 isolates were aligned to 4 ASVs (ASV0024, ASV0031, ASV0350, and ASV7217; Supplementary Fig. 13). Among these ASVs, ASV0024 and ASV0031 were the most abundant in the strain Sy2-11 treated groups (Supplementary Fig. 13) and were also prominent *Bacillus* taxa in the network. These results demonstrate the successful isolation of core microbiota, which will facilitate subsequent experimental validation.

### Strain Sy2-11 mediates *Bacillus* spp. recruitment via root exudate alteration

**Fig. 4:**
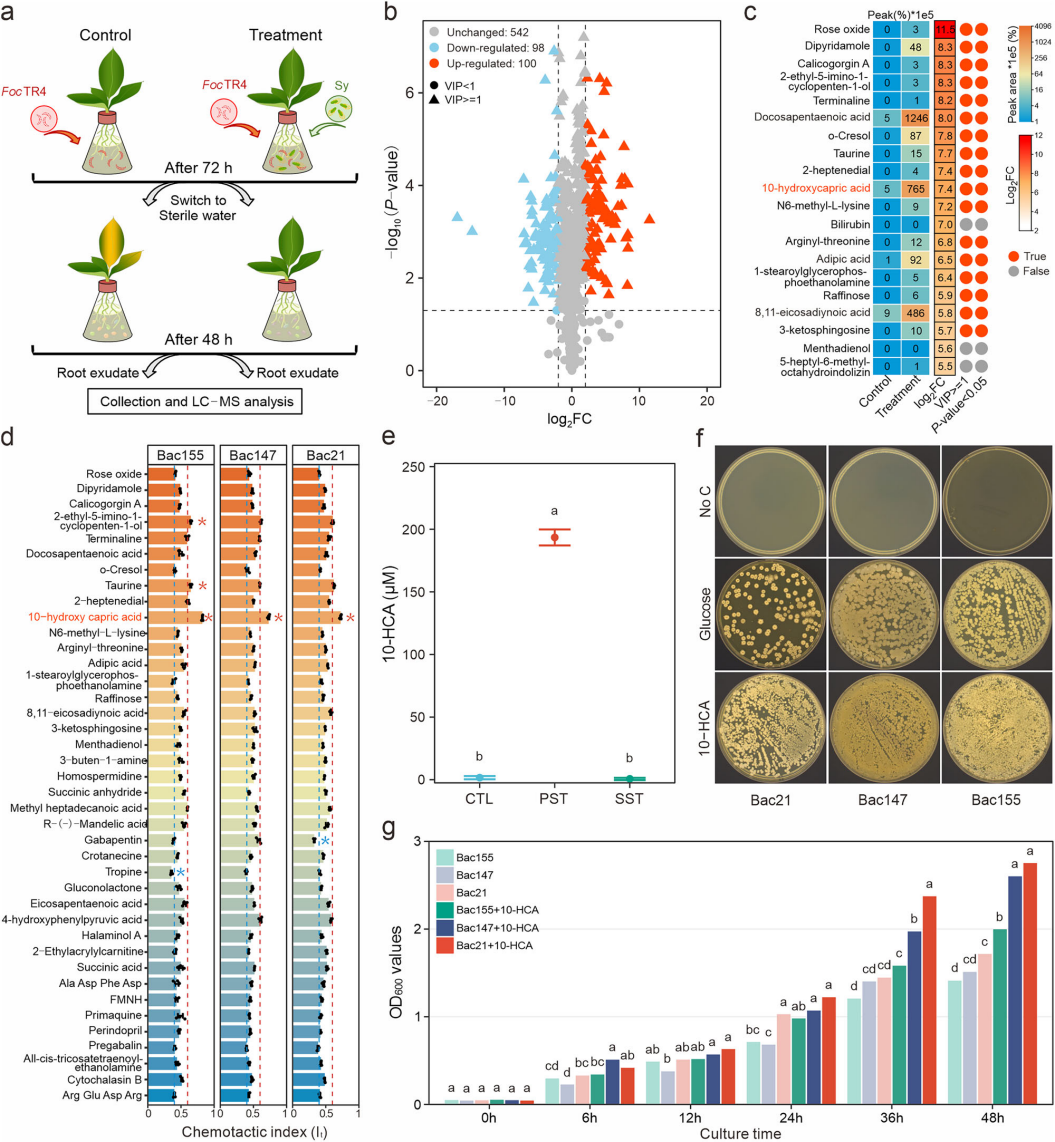
10-HCA in root exudates induces enrichment of disease-suppressing *Bacillus.* **a** Flowchart depicting the methodical collection process of banana root exudates. **b** Volcano plot illustrating differential metabolites. **c** Candidate differential metabolites, highlighted in orange, were identified based on multiple criteria. Statistical significance was assessed using two-sided Student’s *t*test (*n* = 3). *P*values were adjusted using the Bonferroni correction. VIP were obtained using the OPLS method in (**b**) and (**c**). **d** Chemotactic responses of *Bacillus* isolates, including Bac155, Bac147, and Bac21, to the candidate differential metabolites are presented. The green and purple dashed lines indicate the chemotactic thresholds of 0.4 (repulsion) and 0.6 (attraction), respectively. Red and blue asterisks denote statistically significant attraction and repulsion, respectively, based on one-sided t-tests (*P* < 0.05, *n* = 3). *P*values in Supplementary Table 1. **e** Changes in 10-HCA concentration in the rhizosphere soil of CTL, PST, and SST groups. **f** The influence of 10-HCA on the growth and phenotypes of *Bacillus* isolates (Bac155, Bac147, and Bac21) is observed after 48 h of cultivation in inorganic salt media supplemented with glucose (5 g/L) and 100 μM of 10-HCA. **g** Growth of *Bacillus* isolates (Bac155, Bac147, and Bac21) was monitored over time following the addition of glucose (5 g/L) and 10-HCA (100 μM), with cell density measured by OD_600_. In (**e**) and (**g**), statistical significance was assessed using LSD multiple comparison tests, with different letters indicating significant differences (*P* < 0.05). Data are presented as mean values ± SEM (*n* = 3).

To explore the mechanisms underlying the recruitment of *Bacillus* spp. in the rhizosphere, we tested the effects of strain Sy2-11 on the metabolic profile of banana root exudates. Root exudates were collected from hydroponic systems of strain Sy2-11-inoculated plants and non-inoculated controls, and analyzed using high-pressure liquid chromatography-mass spectrometry (HPLC-MS) (Figs. 1a, 4a). Orthogonal partial least squares-discriminant analysis (OPLS-DA) showed that strain Sy2-11 inoculation significantly altered the exudate composition (Permutation test, R^2^X  =  0.88 and Q^2^  =  0.998, Supplementary Fig. 14). To identify key metabolites, we applied stringent criteria ( | log_2_FC | ≥ 2, VIP ≥ 1, *P* < 0.05), yielding 198 differential metabolites, of which 98 downregulated and 100 upregulated in the strain Sy2-11 treated group (Fig. 4b, c). We then used microfluidic chemotaxis assays to evaluate the chemotactic responses of *Bacillus* isolates to the top 40 upregulated metabolites. Key strains, including *B. velezensis* (Bac155), *B. xiamenensis* (Bac147), and *B. subtilis* (Bac21), exhibited strong chemotaxis towards 10-hydroxycapric acid (10-HCA) (It > 0.6) (Fig. 4d). Thus, 10-HCA was identified as a key compound in banana root exudates responsible for recruiting *Bacillus* spp. with disease-suppressing capabilities.

To elucidate the role of 10-HCA in recruiting *Bacillus* spp., we measured the concentration of 10-HCA in the rhizosphere soil. Quantitative analysis revealed that 10-HCA was present in the rhizosphere soil of the PST group at a concentration of 193.45 μM, while was undetectable in the CLT and SST groups (Fig. 4e). Next, we tested its in vitro effects on the growth and biofilm formation of selected *Bacillus* isolates. Results indicated that 10-HCA at concentrations of 10 μM and 100 μM promoted the growth and biofilm formation of *Bacillus* isolates (Bac155, Bac147, and Bac21) compared to conventional carbon sources (D-fructose, glucose, D-mannitol, and sucrose) (Supplementary Fig. 15). Specifically, 100 μM of 10-HCA was the optimal concentration for enhancing growth and biofilm formation, significantly outperforming glucose (Fig. 4f, g). Conversely, 1000 μM 10-HCA inhibited these processes. To visualize their interactions, bacterial cultures were diluted and spread on TSA agar plates. with colony counts indicating that 100 μM 10-HCA significantly increased the abundance of *Bacillus* isolates compared to glucose (Fig. 4f). Therefore, 100 μM 10-HCA served as a preferred carbon source for stimulating growth and biofilm formation of *Bacillus* isolates, rather than acting solely as a signal inducer (Supplementary Fig. 16). In the meanwhile, we also tested the effect of 10-HCA on the growth of strain Sy2-11 under in vitro conditions. We observed that 10-HCA at concentrations of 10 μM and 100 μM significantly promoted the growth of strain Sy2-11 compared with conventional carbon sources (D-fructose, D-glucose, D-mannitol, and sucrose) (Supplementary Fig. 17). Specifically, relative to sucrose (the optimal carbon source), 10 μM and 100 μM 10-HCA can increase the cell number of strain Sy2-11 by 2.22-fold and 5.75-fold, respectively. In contrast, 1000 μM 10-HCA inhibited the growth of strain Sy2-11 (Supplementary Fig. 17). Thus, 10-HCA positively regulates the growth of both *Bacillus* isolates and strain Sy2-11, functioning as both a nutritional carbon source and a regulatory signaling molecule.

### *Streptomyces*-derived VOCs trigger 10-HCA biosynthesis in banana

**Fig. 5:**
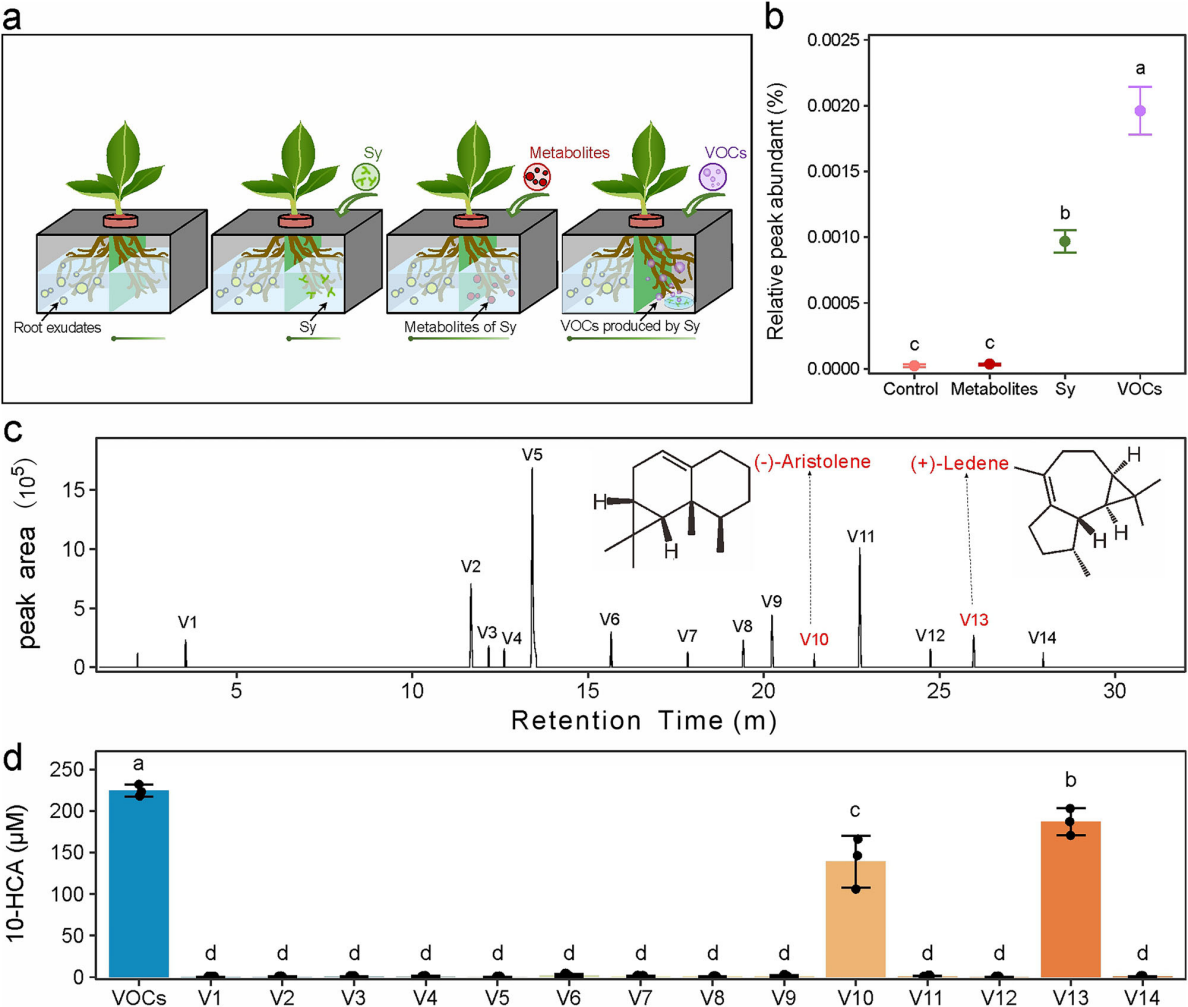
*Streptomyces* volatile organic compounds (VOCs) stimulate banana root to secrete 10-HCA. **a** Flowchart of the experimental design for treating banana roots with strain Sy2-11, its metabolites, and its VOCs and collecting root exudates in a split-root system. **b** The content of 10-HCA in banana root exudates following treatment with strain Sy2-11, its metabolites, and its VOCs. **c** Analysis of VOCs from strain Sy2-11 by GC-MS. Methyl 2-methylbutyrate (V1), 2-Ethylhexanol (V2), methyl 2-ethylhexanoate (V3), 4,7-Dimethylundecane (V4), (2-methoxyethyl) benzene (V5), 2-ethylhexyl acetate (V6), 1H-Indene, 1-ethylideneoctahydro-7a-methyl-, cis- (V7), Trimethyloctane (V8), octyl 2-methylprop-2-enoate (V9), (-)-Aristolene (V10), ( + /-)-Geosmin (V11), Germacrene D (V12), ( + )-Ledene (V13), (S,3E,7E)-α,α,4,8-Tetramethyl-3,7-cyclodecadiene-1-methanol (V14). **d** Changes in the absolute content of 10-HCA induced by different VOCs. In (**b**) and (**d**), Statistical significance was assessed using LSD multiple comparison tests, with different letters indicating significant differences (*P* < 0.05). Data are presented as mean values ± SEM (*n* = 3).

To further explore the elicitors that trigger the biosynthesis of 10-HCA in bananas during the interaction of strain Sy2-11 and banana, we established a split root system of banana seeding culture, and conducted an assay that treated banana roots with strain Sy2-11, candidate metabolites, and VOCs, respectively (Fig. 5a). Subsequently, the composition of root exudates was analyzed using HPLC-MS, and the results showed that treatment with strain Sy2-11 and its derived VOCs significantly increased the relative contents of 10-HCA (*P*  <  0.05, Fig. 5b). This observation confirmed that strain Sy2-11 can via its derived VOCs eliciting 10-HCA biosynthesis in banana. To identify the key functional compounds in the VOCs, the gas chromatography mass spectrometry (GC-MS) analysis was employed to profile the strain Sy2-11 derived VOCs. The results showed that the 14 principal VOCs were identified, including Methyl 2-methylbutyrate, 2-Ethylhexanol, methyl 2-ethylhexanoate, 4,7-Dimethylundecane, (2-methoxyethyl) benzene, 2-ethylhexyl acetate, 1H-Indene, 1-ethylideneoctahydro-7a-methyl-, cis-, Trimethyloctane, octyl 2-methylprop-2-enoate, (-)-Aristolene, ( + /-)-Geosmin, Germacrene D, ( + )-Ledene, (S,3E,7E)-*α*,*α*,4,8-Tetramethyl-3,7-cyclodecadiene-1-methanol (Fig. 5c). These 14 VOCs were then selected to further identify the eliciting factors of *Streptomyces* triggering the secretion of 10-HCA in banana. Interestingly, we discovered that the contents of 10-HCA were increased to 139.15 μM and 186.85 μM under either treatment of aristolen and ledene, respectively (Fig. 5d). These two VOCs were sesquiterpenes with the chemical formula C_15_H_24_. These findings indicate that aristolen and ledene from *Streptomyces* may serve as elicitors to trigger 10-HCA biosynthesis in banana root.

### In situ validation of 10-HCA effects on disease incidence and rhizosphere microbiome

**Fig. 6:**
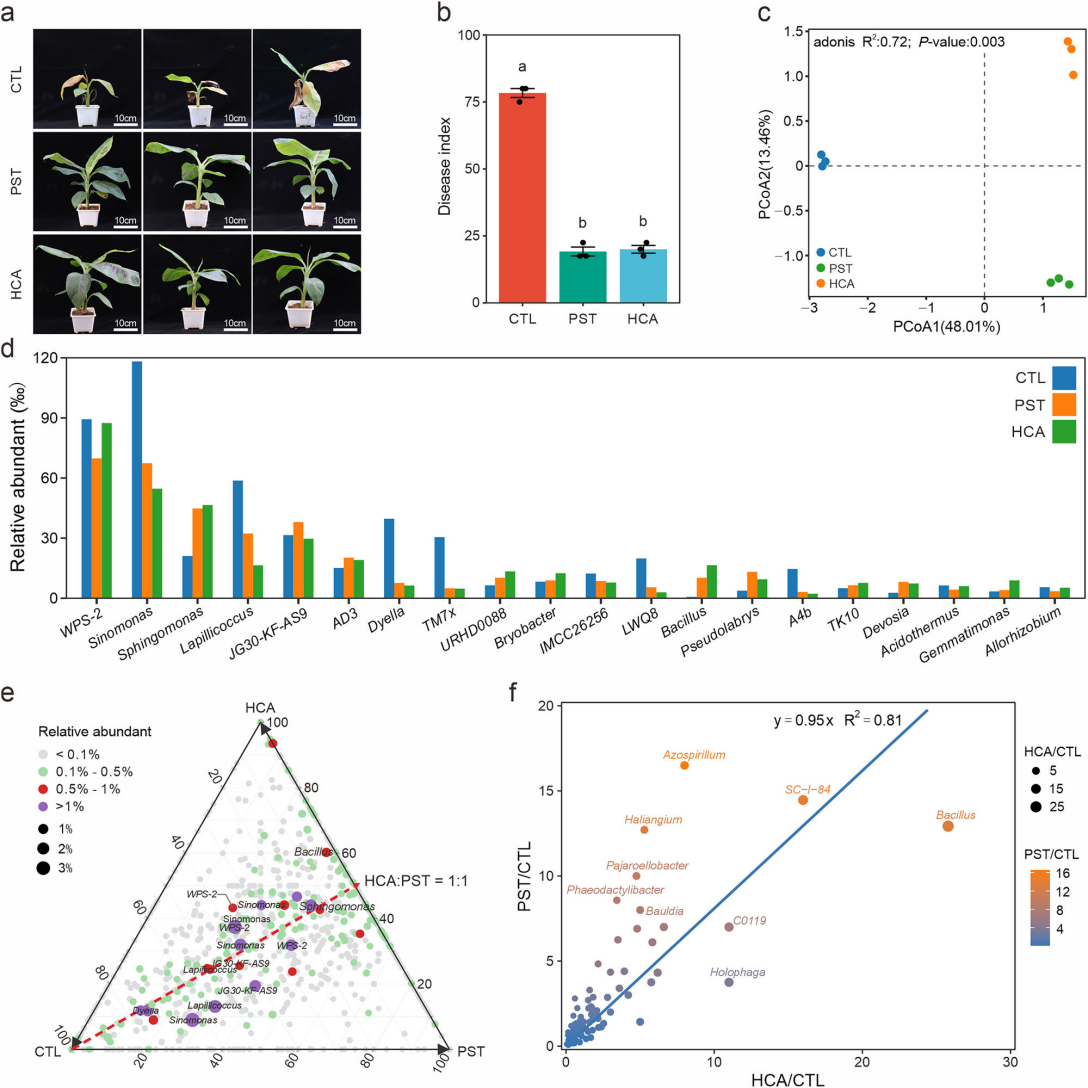
Impact of 10-HCA on wilt disease incidence and rhizosphere microbiome of banana plants. **a** Phenotypic comparison of banana seedlings following treatment with strain Sy2-11 and 10-HCA. Seedings in the 10-HCA and PST groups showed healthy growth, while those in the CTL group exhibited obvious symptoms of *Fusarium* wilt disease. Images were taken at 35 dpi. **b** Disease index comparison among the CTL, PST, and 10-HCA groups. Statistical significance was assessed using LSD multiple comparison tests, with different letters indicating significant differences (*P* < 0.05). Data are presented as mean values ± SEM (*n* = 3). **c** Bacterial *β*-diversity in the rhizosphere soils was analyzed using principal coordinate analysis (PCoA). The symbol colors in (**c**) correspond to the legend provided in (**d**). The R² and *P* value were derived from a PERMANOVA (permutational multivariate analysis of variance) test (*n* = 3). **d** Bar chart illustrating the relative abundance of the top 20 genera. **e** Ternary plot revealing the overall distribution similarity between the PST group and the 10-HCA group. Colors (gray, green, red, and purple) and point sizes indicate different levels of ASV relative abundance, as explained in the legend. The red dashed line represents an HCA:PST ratio of 1:1. Several highly abundant genera are labeled with text. **f** Genus-level fold change analysis relative to the CTL group revealed a strong similarity in microbial profiles between the HCA and PST treatments. Besides, *Bacillus* was identified as the most significantly enriched genus in both the PST and 10-HCA groups compared to the CTL group. CTL: control treatment consisted of plants treated with sterilized water; PST: plants inoculated with strain Sy2-11; HCA: plants inoculated with 10-HCA.

To further verify the role of 10-HCA in microbe recruitment to suppress banana wilt disease, we conducted a pot experiment with three distinct treatments: plants treated with (1) water as a control, (2) strain Sy2-11, and (3) 10-HCA. Results showed that after challenging the seedlings with *Foc* TR4, seedlings in both the strain Sy2-11 and 10-HCA treatments remained healthy, in contrast to the control (Fig. 6a, b). Microbial community analysis revealed no significant differences in *α*-diversity indices between the strain Sy2-11 and 10-HCA groups, but both exhibited significant differences compared to the control group (Supplementary Fig. 18a–d). The *β*-diversity analysis showed that the three treatments formed three distinct clusters. The strain Sy2-11 and 10-HCA groups were clustered distinctly to the control group, explaining 48.01% of the variation along PCoA1. The clusters of strain Sy2-11 and 10-HCA groups spread along PCoA2, which explained 13.46% of the variation (Fig. 6c). Results showed that the treatments have substantial effects on banana rhizosphere microbiome assembly. The strain Sy2-11 and 10-HCA treatment clusters can only be separated along PCoA2, reflecting their more shared analogous microbial compositions. The clear separation of these two clusters indicates that 10-HCA treatment and strain Sy2-11 inoculation similarly alter rhizosphere microbiome assembly, although strain Sy2-11 may involve additional, yet unidentified, mechanisms.

Among the differentially presented taxa, the genera *Sphingomonas*, *Bacillus*, and *Pseudolabrys* were significantly more abundant in the strain Sy2-11 and 10-HCA groups than in the control group, while the genera *Sinomonas*, *Lapillicoccus*, and *Dyella* showed an opposite trend (Fig. 6d). The majority of the ASV abundances were distributed along the mid-vertical line from the control group to the strain Sy2-11 and 10-HCA groups (Fig. 6e), further corroborating the similarity in microbial compositions between the strain Sy2-11and 10-HCA groups. This pattern also indicates that 10-HCA can induce the directional assembly of microbes, mirroring the assembly observed with strain Sy2-11 treatment. Comparative analysis revealed that *Bacillus* was the most abundant genus with the most significant fold change in the 10-HCA group (Fig. 6f), suggesting that the mechanism of suppressing wilt disease is likely consistent with the way strain Sy2-11 recruits *Bacillus* spp. to suppress *Foc* TR4, resulting in robust growth of banana plants (Supplementary Fig. 18e–k).

### Construction of *Bacillus* SynComs and their disease-suppressing ability

**Fig. 7:**
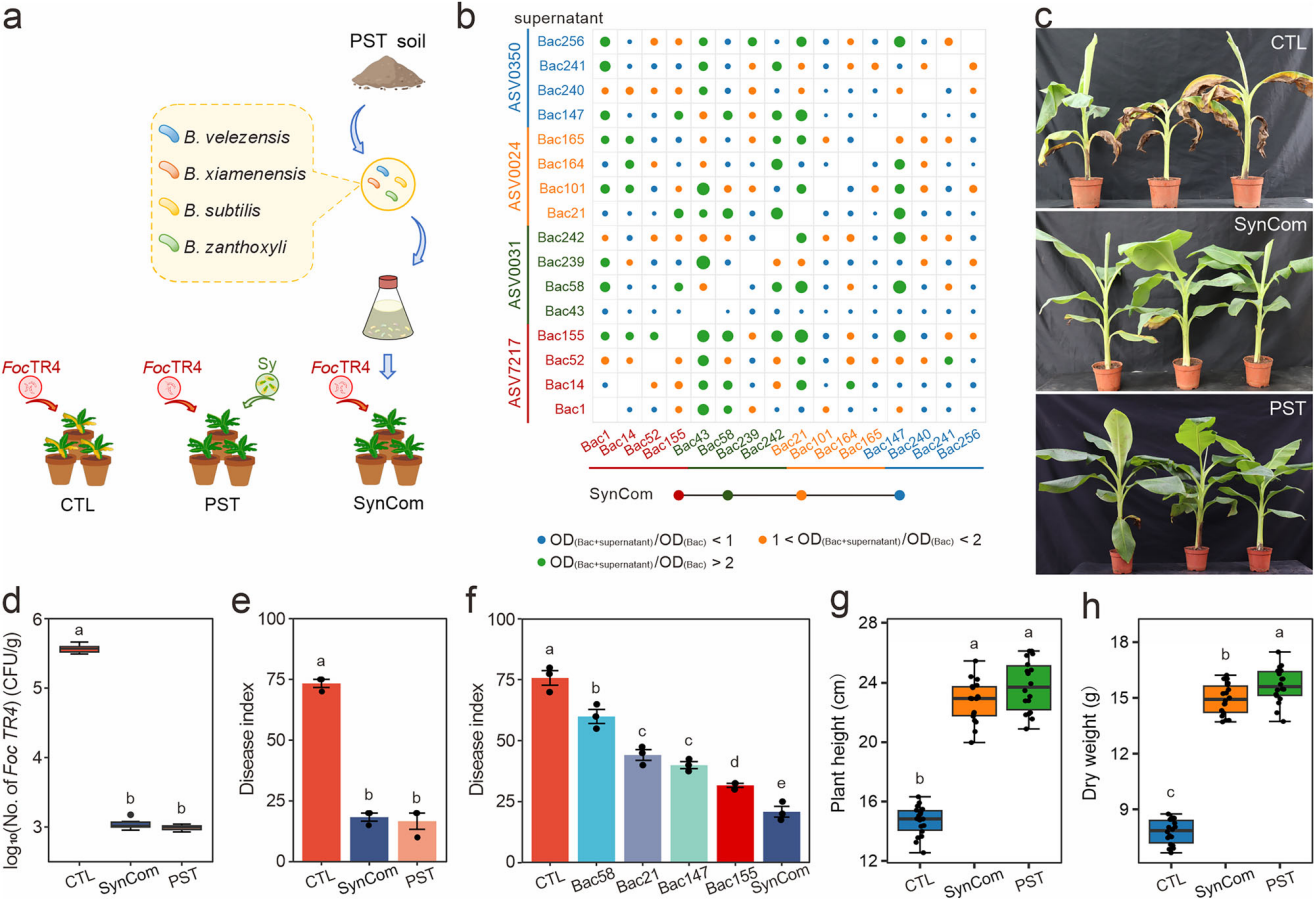
Construction of SynComs using *Bacillus* sp. isolates and validation of suppression ability on *Foc* TR4 and BFW. **a** Schematic representation of the construction process for SynComs. **b** Construction of SynComs and pair interaction of different ASVs *Bacillus* isolates. The size of the circle represents OD_(Bac+supernatant)_/OD_(Bac)_, and the growth-promoting effect of the supernatant on the other *Bacillus* becomes stronger with the increase of the circle. The blue, orange, and green circles represent OD_(Bac+supernatant)_/OD_(Bac)_ < 1, 1 < OD_(Bac+supernatant)_/OD_(Bac)_ < 2, and OD_(Bac+supernatant)_/OD_(Bac)_ > 2, respectively. **c** Growth phenotypes of germ-free banana seedlings inoculated with *Foc* TR4 alone, *Foc* TR4 in combination with SynComs, and *Foc* TR4 in combination with strain Sy2-11, observed at 35 dpi. **d** Comparison of *Foc* TR4 levels in the rhizosphere soil of CTL, SynComs, and PST groups (*n*  =  6). **e** Disease indexes of banana seedlings in CTL, SynComs, and PST groups after *Foc* TR4 inoculation at 5 weeks post-inoculation. Data are presented as mean values ± SEM (*n* = 3). **f** Disease indexes of banana seedlings inoculated with individual *Bacillus* isolates constituting SynComs. Data are presented as mean values ± SEM (*n* = 3). Growth phenotypes of banana seedlings in CTL, SynComs, and PST groups (*n*  =  6 biologically independent soil samples). The parameters measured include (**g**) plant height, (**h**) dry weight (*n*  =  10). CTL: control treatment consisted of plants treated with sterilized water; PST: plants inoculated with strain Sy2-11; SynCom: plants inoculated with SynCom. Source data are provided as a Source Data file. In (**d–h**), Statistical significance was assessed using LSD multiple comparison tests, with different letters indicating significant differences (*P* < 0.05). In (**d**), (**g**), (**h**), Horizontal bars within boxes represent the median. T**h**e tops and bottoms of boxes represent 75th and 25th quartiles, respectively. The upper and lower whiskers represent the range of non-outlier data values.

The construction of SynComs is crucial for verifying microbiome function, studying plant-microbiome interactions, and translating these findings into field applications. We selected four *Bacillus* strains corresponding to ASVs, including *B. velezensis* (Bac155), *B. xiamenensis* (Bac147), *B. subtilis* (Bac21), and *B. zanthoxyli* (Bac58), to construct a SynComs (Fig. 7a). To maximize the disease-suppressing ability of *Bacillus* and exclude the inhibitory effect of metabolites from one strain on the growth of others, we constructed SynComs based on the non-antagonistic interactions between strains. Based on the antagonistic activity of *Bacillus* isolates against *Foc* TR4 and sequence alignments with ASVs, we selected sixteen unique *Bacillus* strains perfectly matched with ASVs, belonging to ASV7217, ASV0031, ASV0024, and ASV0350 (Fig. 7b). Pairwise interaction experiments of these strains indicated that most *Bacillus* strains could coexist with each other and showed positive interaction. However, there were negative interactions between several pairs of strains. Due to the significant promotion of the growth of most other strains by the supernatant of Bac155, we chose Bac155 as the core strain to construct a SynCom by a balanced combination of bottom-up strategies (Fig. 7b). The SynCom group significantly enhanced the resistance of banana plants against BFW (Fig. 7c) and reduced the abundance of *Foc* TR4 in the rhizosphere (Fig. 7d). The effect of SynCom on the disease index was evaluated. Results showed that the disease index in the CTL group was 73.33%, and inoculation of the SynCom significantly reduced disease incidence to 18.33%, which is similar to the disease suppression level of the strain Sy2-11 treatment group (disease index of 16.67%) (Tukey’s HSD test, *P*  <  0.05; Fig. 7e). To determine the contribution of each isolate to the disease suppression, we performed the drop-out SynCom experiments, excluding an individual isolate each time. Further pot experiments with individual strains demonstrated that the control effect was the result of the combined action of all the strains, with Bac155 contributing the most (66% of SynCom’s effect) (Fig. 7f). This finding aligns with in vitro observations of antagonistic activity against *Foc* TR4 (Supplementary Fig. 11). Additionally, the SynCom significantly increased the plant height, pseudostem girth, fresh weights, dry weights, and chlorophyll content (Fig. 7g, h**;** Supplementary Fig. 19). Collectively, these *Bacillus* isolates exhibited a significant suppressive effect on BFW, providing reliable evidence that *Bacillus* taxa were core microbiota recruited by banana roots for disease resistance.

### The chemotaxis-related gene *cheW* regulates the chemotaxis behaviors of *Bacillus* spp. towards 10-HCA

**Fig. 8:**
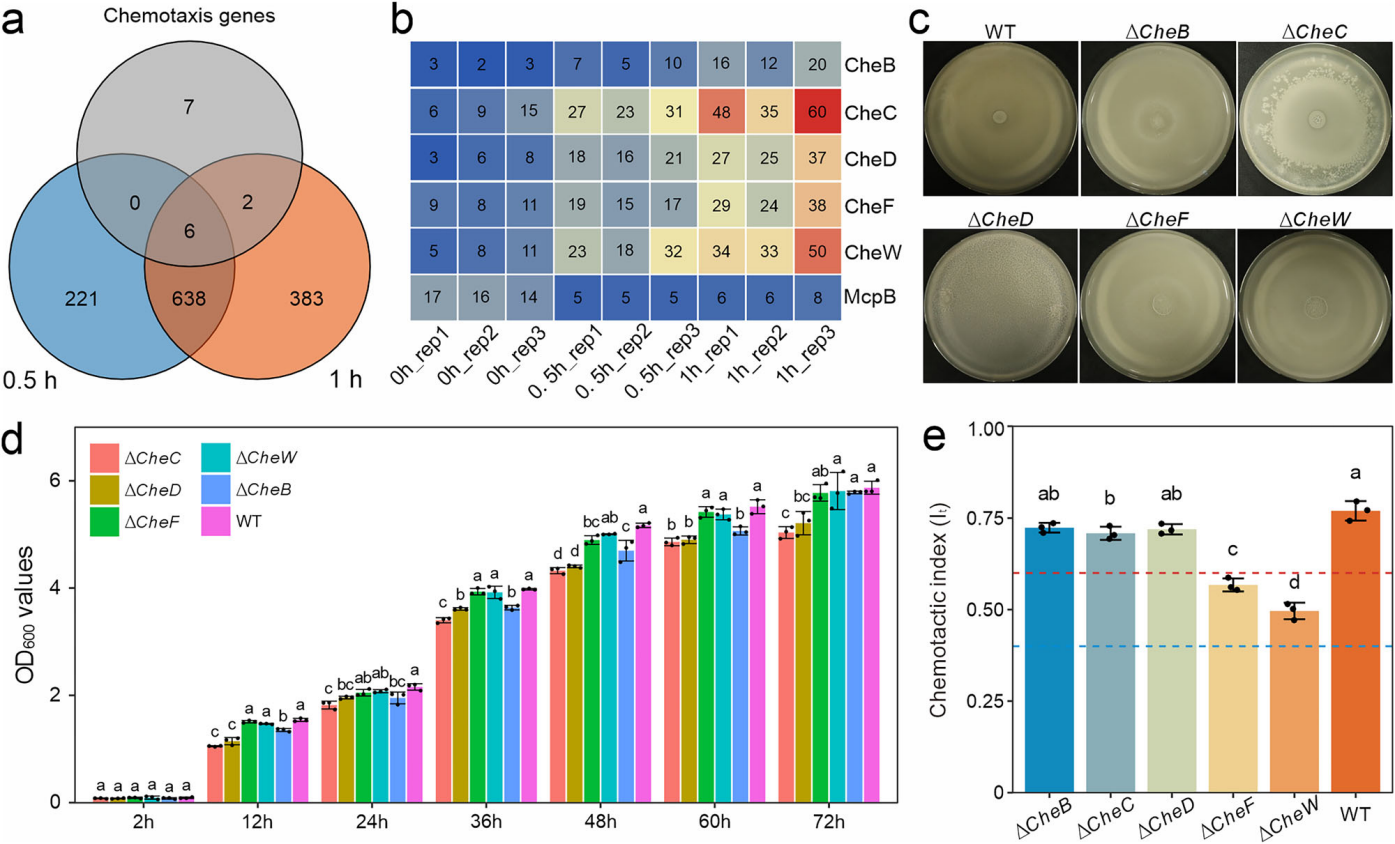
Identification of chemotaxis-related genes and their role in the chemotaxis of *B. velezensis* towards 10-HCA. **a** 865 and 1029 DEGs were identified at 0.5 h and 1 h, respectively. **b** A total of 6 differentially expressed chemotaxis genes were identified. Both numbers and colors display the gene expression with FPKM values. **c** Growth characteristics of mutant and wild-type (WT) strains on TSA medium. **d** Growth curves of mutant and WT strains on TSA medium. **e** Chemotactic responses of mutant and WT strains towards 10-HCA. The blue and red dashed lines indicate the chemotactic thresholds of 0.4 (repulsion) and 0.6 (attraction), respectively. In (**d**) and (**e**), Statistical significance was assessed using LSD multiple comparison tests, with different letters indicating significant differences (*P* < 0.05). Data are presented as mean values ± SEM (*n* = 3).

To gain a more in-depth understanding of the correlation between 10-HCA and the functions of enriched *Bacillus* spp., we performed RNA-seq sequencing on *B. velezensis* (Bac155) treated with 10-HCA for 0.5 h and 1 h. Results showed 865 and 1029 differentially expressed genes (DEGs) were identified at 0.5 h and 1 h, respectively ( | log_2_FC | ≥1 & *P* < 0.05; Supplementary Data 3). Based on the genome sequencing, we identified 15 chemotaxis genes in *B. velezensis*. The Venn diagram revealed that a total of 6 differentially expressed chemotaxis genes were identified (Fig. 8a). Among them, *McpB* was down-regulated, while *CheB*, *CheC*, *CheD*, *CheF*, and *CheW* were all up-regulated (Fig. 8b). These results suggested that the expression of motility-related genes was increased under 10-HCA treatment. Thus, we hypothesized that the motility-related gene plays an important role in the chemotaxis of *Bacillus* sp. towards 10-HCA. To further elucidate the gene function, we successfully knocked out these 5 genes, including *CheB*, *CheC*, *CheD*, *CheF*, and *CheW*, using the homologous recombination method. The mutant strains exhibited phenotypes largely similar to the wild-type strain (Fig. 8c). The growth rates of most mutant strains were comparable to those of the wild type on TSA medium (Fig. 8d). Moreover, quantitative chemotaxis of the six mutant strains toward 10-HCA were assayed. The results showed that mutant strains *ΔcheB*, *ΔcheC*, and *ΔcheD* still exhibited obvious chemotaxis toward 10-HCA. However, the chemotactic ability of *ΔcheF* was impaired, whereas *ΔcheW* completely lost the chemotactic behavior (Fig. 8e). Overall, *CheW* is an important regulatory gene for *B. velezensis* to move towards 10-HCA and towards banana roots. To further verify the role of *CheW* in the *Bacillus* spp. chemotaxis and disease-suppressive, we performed an in situ validation of strain Bac155 and the *ΔcheW* mutant strain via a compartmentalized rhizobox system. Analysis of soil samples revealed that the T*ΔcheW* group still exhibited a significantly higher *Foc* TR4 abundance (2.87 × 10^4^ CFU/g soil) compared to the CTL group. In contrast, the *Foc* TR4 abundance in the rhizosphere of TBac155 group was significantly reduced (3.82 × 10³ CFU/g soil, *P* < 0.05, Duncan’s test). The results further showed that the disease indices of the CTL group and T*ΔcheW* group were 70.83% and 65.28%, respectively, while that of the TBac155 group was only 29.17%. This indicates that strain Bac155 migrated towards and colonized the rhizosphere where 10-HCA was applied, thereby effectively reducing the incidence of *Foc* TR4 (Supplementary Fig. 20).

## Discussion

The rhizosphere microbiome is pivotal in plant stress resistance and pathogen suppression^2,28,29^. Its assembly is shaped by host genotype, growth and developmental stages, root exudates, pathogen invasion, and beneficial microbial colonization^30–32^. Previous studies have shown that when pathogen attacks can trigger plants emit “cry for help”, whereby plants recruit beneficial microbes from the soil to reconfigure the rhizosphere microbiome and enhance disease resistance^33,34^. This mechanism is increasingly recognized as a major driver of disease-suppressive microbiome assembly^14,15,35^. However, the pathogenicity of the elicitor that triggers this response has limited agricultural applications. Here, we propose “rhizosphere microecological regulation”, which involves the external addition of beneficial microorganisms to establish a stable microecological balance among plants, the rhizosphere microbiome, and pathogens, thereby enhancing plant resistance to pathogens.

In this study, we employed *Streptomyces* strain Sy2-11 to investigate its inhibitory effects against BFW and its impact on the rhizosphere microbiome. *Streptomyces* has been widely recognized for plant disease control, primarily through multiple strategies such as producing antifungal metabolites, hydrolases, and VOCs to suppress pathogenic fungi^8,10,36^. Similarly, niphimycin C produced by strain Sy2-11 exhibits anti‑*Foc* TR4 activity in vitro^10^, yet its practical application faces significant challenges. The synthesis of antifungal compounds in *Streptomyces* requires specific conditions (e.g., stable C:N ratio, pH>7.0), and these compounds may be produced at lower levels or become less stable in non-native environments. In addition, effective suppression of BFW usually requires sustained secretion of antifungal metabolites, which is difficult to maintain under open-field conditions. These constraints might explain that the suppression of BFW by strain Sy2-11 is not attributable to simple direct antagonism but instead relies on more effective indirect mechanisms. Concentration-response experiments further support this conclusion (Supplementary Fig. 6). These findings suggest that the biocontrol function of strain Sy2-11 largely depends on its ability to reshape the rhizosphere microbiome. By contrast, our study revealed a specific disease resistance mechanism in *Streptomyces*, distinct from many previous reports. Specifically, strain Sy2-11 acts as an “elicitor”, reshaping the rhizosphere microbiome by recruiting beneficial core microbiota *Bacillus* sp. as partners, and assisting in *Fusarium* wilt disease resistance (Fig. 2). Although previous studies have reported that exogenous beneficial microbes can enrich disease-suppressive taxa such as *Flavobacterium*, *Pseudomonas*, *Bacillus*, *Chryseobacterium*, *Lysobacter*, *Agrobacterium*, and *Lysinibacillus*^37–40^, *Streptomyces* has not previously been implicated as inducers or recruitment factors for partner microbes. Our findings identify *Streptomyces* as a potential “elicitor” that promotes the formation of a disease-suppressive rhizosphere microbiome (Fig. 2), which may be beneficial for agricultural applications.

Our results further indicate that strain Sy2-11 reshapes the rhizosphere microbiome by altering the key root exudates of the banana. In particular, *Bacillus* species with antifungal activity were specifically enriched and acted as cooperative partners to suppress pathogens in plant roots and soil, which differs from previous findings. Previous studies have demonstrated that some plant growth-promoting rhizobacteria (PGPR) recruit indigenous beneficial bacteria as cooperative partners via cross-feeding and nutritional interactions mediated by their secondary metabolites, thereby promoting plant growth and suppressing diseases^40^. Root exudates are central mediators of plant-microbe interactions, including sugars, amino acids, organic acids, flavonoids, and nucleotides that attract microbes from the bulk soil into the rhizosphere^16,41^. Previous studies have shown that plants under stress or microbial induction often altered the secretion of flavonoids (e.g., xanthine), amino acids (e.g., proline), and organic acids (e.g., malic acid and citric acid)^18,42^. Here, we identified 10-HCA as a key chemoattractant metabolite in root exudates responsible for recruiting core microbiota (Fig. 4). 10-HCA was significantly increased in the root exudate of banana with the third highest log_2_FC value after strain Sy2-11 inoculation, and representative *Bacillus* isolates Bac21, Bac147, and Bac155 showed strong chemotaxis toward 10-HCA (Fig. 4c, d; Supplementary Table 1). 10-HCA is a saturated fatty acid with antifungal and anti-inflammatory properties^43^. Other fatty acids, including docosapentaenoic acid, adipic acid, eicosapentaenoic acid, and 4-hydroxyphenylpyruvic acid, were also detected in this study and significantly enriched in the *Streptomyces*-treated roots (Fig. 4b; Supplementary Data 4). Previous studies have demonstrated that rhizosphere bacteria prefer aromatic organic acids secreted by plants (nicotinic, shikimic, cinnamic, and salicylic)^28^. Similarly, fatty acid could trigger *Bacillus* chemotaxis, resulting in heightened motility^40^. Interestingly, our results further indicated that 10-HCA, as “food” for *Bacilus*, plays a crucial role in shaping microbial community structure and multifunctionality, thereby limiting the ecological niche for the pathogen to survive^44^. Previous studies have demonstrated that upon pathogen infection, plants secrete flavonoids, acting as signaling molecules to initiate chemotaxis or symbiotic interactions with beneficial microbes^18,42,45^. By contrast, we propose a distinct “cry for help” mechanism in which 10-HCA, a dual-functional mediator, serves as both the signaling molecule and nutrition of *Bacillus* in the *Streptomyces*-induced response. Specifically, it not only elicits strong chemotaxis in core *Bacillus* isolates but also provides a direct nutrient source to promote their enrichment and enhance rhizosphere microbiome stability. This unique dual functionality sets our mechanism apart from flavonoid-mediated systems. Nonetheless, we cannot eliminate the likelihood that other root exudates may also contribute to the microbial community alterations under the *Streptomyces*-induced conditions. Additional studies are necessary to unravel if and how 10-HCA interacts with other root exudates to regulate *Bacillus* sp. enrichment.

*Streptomyces* mediates alterations in plant root exudates, and the underlying molecular mechanisms remain poorly understood. Interestingly, we identified the aristolene and ledene as signaling molecules released by *Streptomyces* strain Sy2-11. They were key players triggering 10-HCA secretion in banana roots (Fig. 5). Sesquiterpenes were C15-terpenoids that consist of three isoprene units, such as aristolene, ledene, *β*-caryophyllene, *α*-farnesene, etc. They are generally considered strong candidates for underground signaling due to their superior diffusivity in complex soil environments^46^. *β*-caryophyllene can stimulate shoot growth and biomass in lettuce plants challenged with *Fusarium oxysporum*^47,48^. Here, we demonstrate that aristolene and ledene function as heretofore unrecognized signaling molecules in the *Streptomyces*-induced biosynthesis of banana root exudates. Importantly, *Streptomyces* are typically slow-growing, sessile, non-motile, and poor colonizers in soil, which limits their direct interaction with plants. Our findings reveal a distinct mechanism whereby *Streptomyces*-derived sesquiterpene VOCs elicit plant stress responses and stimulate 10-HCA biosynthesis, overcoming the limitation imposed by their poor motility. This study, therefore, provides a promising strategy for the efficient utilization of *Streptomyces*.

Sesquiterpenes are the key mediators during plant-plant communication, within-plant self-signaling, and plant-microbe interactions. However, the receptors and downstream signaling pathways through which plants perceive these VOCs remain poorly understood. In *Petunia*, the karrikin-insensitive receptor PhKAI2ia has been shown to stereospecifically perceive (-)-germacrene D and activate a KAI2-dependent signaling cascade that modulates plant fitness^49^. We identified five homologs of the KAI2 gene family in banana, among which only the g24040 gene was significantly upregulated at 12 h (Supplementary Fig. 21). Additionally, we also identified 11 banana homologs encoding TOPLESS-like proteins, which have been implicated in VOC sensing in tobacco and shown to possess *β*-caryophyllene-binding activity^50^. Among these, g12130, g09280, and g04820 were significantly downregulated at both 6 h and 12 h (Supplementary Fig. 21). These transcriptional responses suggest that sesquiterpenes, particularly ledene, may be perceived by the banana root system and may modulate the expression of candidate receptor-associated genes. To further elucidate the 10-HCA biosynthetic pathway, transcriptomic analysis identified four enzymes involved in fatty acid anabolism, including fatty acyl-CoA-binding enzyme (g12360), fatty acid desaturase (g03200/g33950), and fatty acid hydroxylase (g20040) (Supplementary Fig. 21). However, full elucidation of the 10-HCA pathway will require functional validation of these candidate genes. Therefore, elucidating their recognition mechanisms and pathways constitutes an intriguing topic for future research, and we aim to clarify these scientific questions one by one in subsequent studies.

Microbes in SynComs interact with each other in multiple ways, including mutual inhibition through the production of metabolites and mutual promotion through cross-feeding^51,52^. In this study, paired antagonism experiments were selected for bacteria that utilize each other’s metabolic resources to facilitate mutual growth. We selected the five isolates of *Bacillus* as a commensal microbial SynCom, which further confirmed that these enriched *Bacillus* sp. exhibit suppression ability on *Foc* TR4 and BFW. These findings align with studies showing that *Bacillus* can antagonize *Fusarium* infection through multiple mechanisms, such as the production of antimicrobial substances, niche competition, biofilm formation, and activation of plant defense responses^40^. Although this SynCom did not comprehensively mirror the whole microbial diversity of the rhizosphere soils, it furnished direct evidence that the enrichment of the core microbiota *Bacillus* was driven by *Streptomyces*-induced effects.

Here, an important question arising from these findings is why *Streptomyces* would induce bananas to recruit *Bacillus* sp.? Within the soil, *Streptomyces* display a wide range of social behaviors, such as “hitchhiking”, “exploration” growth, and inducing plant resistance, etc., rendering it a valuable model for examining cooperative group behavior and the evolutionary stability^53,54^. Notably, they display inducible exploratory behaviors, such as accelerated colony expansion through non-branching vegetative hyphal, as well as intricate sporulation mechanisms and diversified transmission modes of spores, enabling them to survive and spread in challenging conditions^55–57^. Previous work has shown that interactions with fungi can stimulate exploratory growth in *Streptomyces* via secreted metabolites and VOCs, enabling them to rapidly traverse solid surfaces and thereby enhancing rhizosphere colonization^58,59^. Additionally, *Streptomyces* spores can attach to flagella of motile bacteria (e.g., *Bacillus subtilis* and *Pseudomonas fluorescens*) and “hitchhiking” to plant root microenvironments^54^. Interestingly, our studies demonstrate that strain Sy2-11 interacts with the *Bacillus* isolates Bac 21, Bac 147, and Bac 155, promoting the extension growth of *Streptomyces* mycelium and expanding its range of movement. In addition, strain Sy2-11 also produces a large amount of fungal hypha-like aerial hyphae (Supplementary Fig. 22). Furthermore, in situ colonization assays showed that *Bacillus* isolates facilitate strain Sy2-11 colonization on banana roots (Supplementary Fig. 23). The hitchhiking dispersal method of *Streptomyces* provides a beneficial colonization strategy by directly transferring its spores to favorable environments, offering a trade-off between benefits and risks. Therefore, *Streptomyces* interacts with bananas, creating a win-win situation, that is, *Streptomyces* releases sesquiterpenes signaling molecules, triggering banana roots to secrete 10-HCA as a “food” for specific *Bacillus*. This attracts *Bacillus* to move directionally and colonize the banana roots, forming a partnership with *Streptomyces* that facilitates the growth of *Streptomyces* and collaboratively targets the suppression of BFW.

**Fig. 9:**
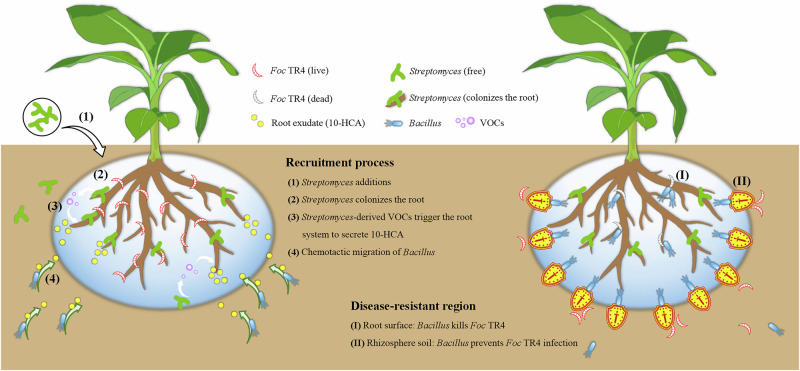
Conceptual overview of *Streptomyces* as an “elicitor” induced 10-HCA secretion by banana roots to recruit *Bacillus* and control *Fusarium* wilt. This conceptual figure illustrates the mechanisms through which *S. yongxingensis* sp. nov. Sy2-11 triggers the secretion of 10-HCA by banana roots, thereby recruiting *Bacillus* species and managing *Fusarium* wilt. The illustration highlights that strain Sy2-11-derived VOCs trigger bananas to release 10-HCA, a substance that acts as a preferred nutrient for *Bacillus* taxa, effectively enlisting these bacteria as allies in the battle against disease and bolstering the plant’s resistance. The established partnership between *Bacillus* sp. and *Streptomyces* facilitates the targeted movement of *Streptomyces*, enabling the colonization of ecological niches in the rhizosphere and assisting banana seedlings in resisting the invasion of BFW.

Collectively, we study on the *Streptomyces*-induced root microbiome of bananas, complemented by subsequent controlled experiments designed to corroborate hypotheses derived from multi-omics data. We have synthesized the results of our experiments into a conceptual model that elucidates the mechanism through which *Streptomyces* application results in the suppression of *Fusarium* wilt disease (Fig. 9). Our results show that *Streptomyces* can release sesquiterpenes signaling molecules to induce bananas to secrete 10-HCA, which serves as “food” for specific *Bacillus* taxa, recruiting those beneficial bacteria as companions and assisting in disease resistance. Moreover, *Bacillus* sp. forms a partnership with *Streptomyces*, facilitating the directed motility and exploration growth of *Streptomyces*. This allows them to occupy ecological niches in the banana rhizosphere and establish themselves at the roots, ultimately assisting bananas in resisting the invasion of BFW. This study will provide theoretical support and effective approaches for “rhizosphere microecological regulation”, and it will play a more active role in sustainable agriculture.

## Methods

### Description of Streptomyces yongxingensis sp. nov

*Streptomyces yongxingensis* (yong.xing.en’sis. N.L. masc./fem. Adj. *yongxingensis* of yongxing, an island in China, referring to the place where the type strain was first isolated). The type strain, Sy2-11^T^ ( = GDMCC4.213 ^T^ = JCM34965^T^), was isolated from the soft coral sample of *Menella woodin* in the ‘yongxing’ island of Hainan province, China. The cultural, physiological, and biochemical characteristics of the type strain are described as follows, as shown in ref. ^10^. The predominant menaquinones are MK9 (H2), MK9 (H6), and MK9 (H8). Major fatty acids ( > 10.0%) are iso-C_15:0_, anteiso-C_15:0_, and C_16:0_ (Supplementary Table 2). The genome of the type strain is characterized by a size of 11,310,836 bp and a G + C content of 71.26%. Raw reads of the type strain Sy2-11 were deposited in the SRA database under accession number JBVLZX000000000, and its 16S rRNA gene sequence was submitted to GenBank under accession number MT122802.

### Experimental design and sampling

To investigate the effects of *Streptomyces*-induced microbial community manipulation on the protection of banana plants in *Fusarium* wilt disease-affected soil, we established a series of microcosm experiments for banana cultivation in a greenhouse at the Chinese Academy of Tropical Agricultural Sciences in Haikou, China. The greenhouse maintained an average temperature of 30 °C and humidity of 70%. The experimental soil was sourced from two fields in Danzhou (109°54'66“E, 19°44′63“N, Field 1) and Lingao (109°54'66“E, 19°44′63“N, Field 2), both with a history of over a decade of banana monoculture and a high incidence of *Fusarium* wilt disease (60 ~ 70%). The soil was evenly distributed into 90 polypropylene pots, each containing 10 kg of soil, and each pot was transplanted with one banana seedling (Musa *acuminata* AAA group, Cavendish cv. ‘BaXiJiao’, a variety susceptible to *Foc* TR4). We established three experimental groups: (1) control treatment consisted of plants treated with sterilized water (CTL), (2) plants inoculated with strain Sy2-11 (PST), and (3) soil (without plants) inoculated with strain Sy2-11 (SST). Here, selected *Streptomyces* at a concentration of 1 × 10^6^ CFU/g soil was applied to the roots of each plant. The mesocosm experiment was performed in a randomized complete block design with three biological replicates, each consisting of thirty pots.

Next, to evaluate the effect of the inoculum dosage of strain Sy2‑11 on the disease index, we performed disease resistance assays using a series of concentrations of strain Sy2‑11. Eight treatments were included: a sterilized-water control (CTL), *Streptomyces* sp. 5-6 (Str. 5-6), and *Streptomyces* sp. H7 (Str. H7) was applied at 1 × 10^6^ CFU/g soil, and strain Sy2-11 was applied at 1 × 10^7^ CFU/g soil, 1 × 10^6^ CFU/g soil, 1 × 10^5^ CFU/g soil, 1 × 10^4^ CFU/g soil, 1 × 10^3^ CFU/g soil. All treatments were subsequently inoculated with *Foc* TR4.

To test the colonization dynamics of strain Sy2-11 in the banana root, a high-resolution upright laser scanning microscope (LSM910, Zeiss, Germany) equipped with two emission-collecting channels was used to examine the banana root colonization patterns of a modified Sy2-11 strain overexpressing green fluorescent protein (GFP-Sy2-11), as described in ref. ^60^. Colonization of banana roots was examined at 3, 7, and 14 dpi. To determine the ability of strain Sy2-11 to colonize the banana rhizosphere soil, rhizoplane, and endorhiza, an ampicillin-resistant marker of strain Sy2-11 was used to quantify its population under axenic conditions, as described by ref. ^60^. Detailed experimental procedures were provided in Supplementary Methods. Disease indexes (DI) were evaluated according to the criteria described by ref. ^10^. Plant growth indicators, including chlorophyll content, leaf area, leaf thickness, stem diameter, plant height, dry weight, and fresh weight, were measured according to previously described methods^8^.

### *Foc* TR4 strains isolation and GFP transformation

To test the colonization dynamics of *Foc* TR4 in the banana rhizosphere, we isolated *Foc* TR4 strain from the diseased soil as described by ref. ^61^. Briefly, ten grams of soil samples were added into 90 mL of sterile water and shaken at 180 rpm/min for 30 min. A 10-fold gradient dilution method was used to dilute the soil leachate, and 100 µL of a 10^-3^ dilution was evenly spread onto a modified Komada’s medium plate^62^. The plates were incubated at 28 °C for 3 d for fungal growth. Specific colonies were selected for the identification of *Foc* TR4 strains by PCR amplification using a series of ITS (ITS1/ITS4), *Fusarium oxysporum*-specific primers (W106F/W106R), *Foc*-specific primers (*Foc*Sc-1/*Foc*Sc-2), and *Foc* TR4-specific primers (*Foc* TR4-F/*Foc* TR4-R) (Supplementary Table 3). The cultural characteristics, morphology of spores and hyphae, and pathogenicity of the identified *Foc* TR4 strain were characterized and assayed according to the method described by ref. ^63^. To further confirm its taxonomic status, the genome of the *Foc* TR4 strain was sequenced using the PacBio Sequel‌ platform (Majorbio Bio-pharm Technology Co., Ltd, Shanghai, China). Based on a comprehensive analysis of phenotypic, morphological, pathogenic, and genomic characteristics, the *Foc* TR4 strain was identified and classified as belonging to the same taxon as the *Foc* TR4 strain No.58 reported in ref. ^64^ (Supplementary Fig. 24). Subsequently, the GFP gene was cloned into the pKNT vector using a one-step cloning kit (Vazyme Biotech, China), and transformed into the GFP-*Foc* TR4 mutant. This transformation allowed for the visualization and tracking of *Foc* TR4 in the rhizosphere, providing valuable insights into its colonization patterns and interactions with banana plants. The spore suspension of GFP-*Foc* TR4 strain was inoculated into the soil at a concentration of 1 × 10^6^ CFU/g soil, following the methodology of ref. ^8^.

### Soil sampling and *Foc* TR4 quantitative analysis

Rhizosphere soil samples were collected according to the method described by ref. ^38^. Samples from each treatment group were immediately stored at -80 °C for subsequent DNA extraction, quantification of pathogen *Foc* TR4 abundances, and isolation of culturable core microbiota. The absolute abundances of *Foc* TR4, expressed as target gene copies per gram of dry soil, were determined using a primer-specific qPCR system with specific primers *Foc* TR4-F and *Foc* TR4-R (Supplementary Table 3), following previously described protocols^39,65^. The population densities of *Foc* TR4 colony-forming units (CFU/g) in the banana rhizosphere were quantified using a modified Komada’s medium^62^.

### DNA extraction, 16S rRNA gene amplicon sequencing, and data processing

Genomic DNA was extracted from all soil samples using the E.Z.N.A.® soil DNA Kit according to the manufacturer’s protocol. DNA concentrations were measured using a NanoDrop spectrophotometer (ND2000, Thermo Scientific, DE, USA). The V3-V4 regions of the 16S rRNA gene were amplified with the primers 338 F and 806 R (Supplementary Table 3). All samples were sequenced on the Illumina MiSeq-PE300 at the Majorbio Bio-Pharm Technology Co., Ltd (Shanghai, China). Data analysis was performed using Qiime2 software (v2022.2.0)^66^. Raw data were processed to remove adapter sequences and primers, and filtered to exclude low-quality reads (sequences with three or more consecutive bases with a quality score below 20), yielding clean data. Paired-end reads were merged using the Vsearch tool, and denoising was performed with the deblur method to generate ASVs. Only ASVs occurring with a frequency greater than 2 and present in at least two samples were retained for further analysis. Taxonomic annotation of ASVs was performed using the Silva database (release 138)^67^. The feature table generated was used for subsequent analyses.

Differentially expressed ASVs were calculated using DESeq2 based on the raw feature tablec^62^. The raw feature table was rarefied using the vegan package in R, and alpha and beta diversity metrics were calculated, along with the generation of bar plots at the genus level. Random forest classification analysis was performed using the randomForest package^68^. LefSe analysis was conducted using the microeco package^17^. For each treatment, ASVs appearing at least twice were retained. A co-occurrence network was constructed based on Spearman correlation coefficients, with network-related attributes calculated using the igraph package^35^.

### *Bacillus* isolation from the banana rhizosphere

To investigate the function and mechanism of action of *Bacillus* species specifically recruited in the banana rhizosphere, we employed a culture-dependent method to isolate *Bacillus* strains. In brief, five grams of soil samples were mixed with 45 mL of sterilized water in a 150 mL Erlenmeyer flask by shaking at 180 rpm for 30 min. The resulting suspensions were then diluted to various concentrations (10^-1^ ~ 10^–6^) using a serial dilution method. One hundred microliters of the dilutions from 10^–5^ to 10^–6^ were evenly spread on three selective separation culture media plates designed for *Bacillus*, namely TSA (tryptic soy agar), NA (nutrient agar), and LB (Luria-Bertani)^69^. *Bacillus* strains were selected and purified three times. The full-length 16S rRNA sequences were amplified and submitted to EzBioCloud (www.ezbiocloud.net/) (Supplementary Table 3). All isolated strains were stored at –80 °C with 20% (v/v) glycerol. The full-length 16S rRNA sequences were aligned using MUSCLE^70^, trimmed with TrimAL^71^, and the optimal model was determined using IQ-TREE^72^ to construct a maximum likelihood phylogenetic tree.

### Antagonistic test of *Bacillus* isolates against *Foc* TR4

The antifungal activity of each *Bacillus* isolate against *Foc* TR4 was detected using the agar diffusion method^8^. Briefly, a 5 mm-diameter disc of *Foc* TR4 was placed at the center of a potato dextrose agar (PDA) plate. Each isolate was then inoculated at four symmetrical points, 2.5 cm from the center of the PDA. A control plate containing *Foc* TR4 but without the *Bacillus* isolate was also prepared. All plates were incubated at 28 °C for 5–7 days to allow for fungal growth and interaction. The diameter of the inhibition zone surrounding each isolate was measured using the cross-crossing method. The inhibition ratio was calculated according to previously described methods^10^.

### Root exudates collection and metabolomic sequencing

To investigate the interaction between strain Sy2-11 and banana, pathogen-free banana tissue-cultured seedlings were transplanted into a sterile hydroponic system for the collection of root exudates (Fig. 4a). The plants were randomly divided into two groups: a treatment group (*Foc* TR4 + *Streptomyces* Sy2-11 spore suspension) and a control group (*Foc* TR4 + sterile water). Each treatment consisted of three replicates, with each replicate containing 20 seedlings. After incubation for 3 days, the plant roots were washed five times with sterile double-distilled water to eliminate any influence from *Foc* TR4 and strain Sy2-11. All plants were then transplanted into a fresh sterile hydroponic system and incubated at 28 °C with a 16 h/8 h light/dark cycle for 48 h to collect root exudates. Additionally, the colonization of strain Sy2-11 on the surface of banana roots was observed using an CLSM at 3 dpi, following the identical method as detailed above. CLSM observations confirmed that strain Sy2-11 was able to colonize the banana root surface efficiently in the treatment group (Supplementary Fig. 25).

To further explore the elicitors that trigger the biosynthesis of 10-HCA in bananas during the interaction between strain Sy2-11 and bananas, a split-root system of plant culture was designed (Fig. 5a). The split-root system was cultured in sterile double-distilled water for an additional two days to check for contamination. Subsequently, the roots on the right side of the chamber were treated with strain Sy2-11, metabolites, and VOCs, respectively. Over the next 3 days, all plants in the systems were cultured at 28 °C with a 16 h/8 h light/dark cycle. Then, the root exudates were collected from the left side of the chamber. The culture solution from the left chamber was subsequently spread onto the YE medium. The results confirmed that the root exudate collection system was free of contamination (Supplementary Fig. 26). Next, a single VOC-treated experiment was conducted using the same method.

The root exudates, which were primarily water-based, were filtered using 0.45 µm filters (Millipore) to remove root debris. The filtrates were freeze-dried and stored at –80 °C for further studies (*n* = 3 biological replicates). This method of collection ensured that no compounds derived from microorganisms were included in the samples. The root exudates were analyzed using a UPLC-ESI-MS/MS system and a Tandem mass spectrometry system at Biomarker Technologies Co., Ltd (Beijing, China). Metabolites were identified using both mass spectra and retention time. Missing values were filtered and filled using the Random Forest model method and normalized using Omicsolution online tools (webtools link). Partial Least Squares Discrimination Analysis (PLS-DA) was used to evaluate the community of metabolites. Differentially expressed metabolites (DEMs) were identified based on the difference in multiple methods, the *P* value, the Fold-Change (FC), and the VIP value of the OPLS-DA model with 200 permutations using the ropls package in R^73^. Statistical significance was considered at FDR-adjusted *P* < 0.05.

### Quantitative chemotaxis assays

The chemotactic behavior of the *Bacillus* isolates was assessed using a microfluidic chip method, with minor modifications to the protocol described by ref. ^74^. *Bacillus* isolates were initially cultured in an inorganic salt medium supplemented with 0.5% glucose (Supplementary Table 4), and cultures were grown to an OD_600_ of approximately 0.6. The cells were collected, washed twice with chemotaxis buffer (30 mM K_2_HPO_4_, 19 mM KH_2_PO_4_, 20 µM EDTA, and 0.05% (v/v) glycerol, pH 7.0), and resuspended in chemotaxis buffer to achieve an OD_600_ of 0.1. The “Y” microchannel of the microfluidic chip was washed twice with chemotaxis buffer, followed by the injection of 5 μL of root exudates solution (100 μM), chemotaxis buffer (negative control), and *Bacillus* suspension into the three chambers of the microfluidic chip (Fig. 1a). The chip was then positioned to connect the two side chambers with the middle chamber, permitting free bacteria movement towards the sides. After incubation at 30 °C in the dark for 30 minutes, the number of *Bacillus* in the side chambers was observed and counted using an inverted fluorescence microscope. The chemotaxis index was calculated by the following formula: I_t_ = N_e_ / (N_e_ + N_c_), where N_e_ represents the number of *Bacillus* cells in the chamber containing the root exudates solution, and N_c_ is the number of *Bacillus* cells in the chemotaxis buffer chamber. According to previously described methods^74^, when I_t_ = 0.4 ~ 0.6, it indicates no chemotactic response to the substance; when I_t_ < 0.4, it suggests avoidance behavior; and when I_t_ > 0.6, it indicates an attractive response.

### Extraction of the rhizosphere soil metabolites

Rhizosphere soil metabolites were extracted according to the method described by ref. ^18^. Briefly, 1.0 g of the soil sample was loaded into a 10 mL centrifuge tube. Add 4 mL of methanol solution (V_methanol_: V_H2O_ = 3:1) and use a ball mill at 45 Hz for 5 min, followed by ultrasonic treatment 5 times, each time lasting for 5 min. The soil suspension was centrifuged at 10,000 × *g* and 4 °C for 15 min, and the supernatant was collected into a fresh tube. A second extraction was performed with ethyl acetate using the same method as above, and the resultant extracts were combined. The concentration of 10-HCA in soil extracts was determined using an HPLC system.

### Effects of 10-HCA on the growth and signal sensing of isolated *Bacillus* strains

To investigate the regulatory roles of 10-HCA on the rhizosphere behavior of recruited *Bacillus* strains, we conducted two distinct experiments to explore the mechanisms underlying directed movement in *Bacillus*, focusing on both nutrient-driven and signal-driven behaviors.

Experiment 1: we initially tested the effects of 10-HCA on the growth of isolated *Bacillus* strains. The strains were inoculated in LB liquid medium at 30 °C with agitation at 180 rpm for 24 h. The cell density was adjusted to an OD_600_ of 0.01 using sterilized water. A 2 µL aliquot of the *Bacillus* suspension was then transferred to a sterile 96-well plate containing 200 µL of inorganic salt medium. Carbon sources (1% w/v) including D-fructose, glucose, D-mannitol, and sucrose were added. 10 μM, 100 μM, and 1000 μM concentrations of 10-HCA were applied as carbon sources, replacing other carbon sources in the bacterial culture in each well with equal volumes. The same volume of medium served as a control. The 96-well plates were incubated at 30 °C with shaking at 180 rpm for 48 hours, and the absorbance at OD_600_ was measured at regular intervals using a SpectraMax M3 (Bio-Tek Synergy H1, USA). The effect of 10-HCA on the growth of strain Sy2-11 was assessed using the identical method described above.

Experiment 2: Subsequently, we assessed the signal response of *Bacillus* isolates to 10-HCA on solid LB agar plates (1.5% agar, w/v). The plates were prepared to evaluate the chemotactic behavior of the isolates towards 10-HCA, providing insights into the signaling pathways involved in the response to this specific compound.

### RNA-seq sequencing and data analysis of banana

To further investigate the recognition process and signaling molecular mechanisms of banana roots in response to sesquiterpene VOCs, we treated banana roots with ledene and collected banana root samples at 0 h, 3 h, 6 h, and 12 h post-treatment, immediately frozen in liquid nitrogen, and stored at –80 °C. Total RNA was extracted, and genomic DNA was removed using the RNA extraction kit (TIANGEN, Beijing, China). RNA integrity and concentration were detected using 1.2% agarose gel electrophoresis and NanoDrop One (Thermo, Wilmington, America). Sequencing was performed on the Illumina NovaSeq X Plus (Majorbio Bio-pharm Technology Co., Ltd, Shanghai, China). Raw data quality was assessed via FastQC (v0.11.9), and adapters, poly-N, and low-quality reads were filtered using fastp (v0.20.0). Clean reads were aligned to the ‘Baxijiao’ reference genome (Banana Genome Hub, https://banana-genome-hub.southgreen.fr/) with Hisat2 (v2.1.0), and alignment quality was evaluated by QualiMap (v2.2.1). Gene expression levels were quantified as FPKM. DEGs were identified using the DESeq2 R package (v1.32.0). The DEGs were identified based on the following criteria: an FC of ≥ 2 and an adjusted p value of <0.05. Protein function annotation was conducted using the eggNOG-mapper (v2.1.9) against the reference database. GO and KEGG enrichment analyses of DEGs were conducted using the clusterProfiler R package.

### Effects of 10-HCA on BFW incidence and the rhizosphere microbiome

To verify the effect of 10-HCA on BFW incidence and the rhizosphere microbial community, we performed an in situ validation of the role of 10-HCA through a mesocosm experiment in a greenhouse. Topsoil (0–25 cm depth) was collected from a banana orchard in Danzhou City (109°54'66“E, 19°44′63“N), Hainan, China, where the incidence of *Fusarium* wilt disease is known to be high (approximately 65%). The soil was promptly transported to the greenhouse for pot experiments. Before use, the topsoil was sifted through a 2 mm diameter screen to remove stones and plant debris. Each plastic pot was filled with 1000 × *g* of soil. Banana plants were multiplied and aseptically planted into plastic pots. The experimental design included three treatments: (1) CTL: control treatment consisted of plants treated with sterilized water; (2) PST: plants inoculated with strain Sy2-11 (1 × 10^6^ CFU/g soil); (3) HCA: plants inoculated with 10-HCA. Each treatment was conducted using 90 plants in three replicates (30 plants per replicate).

The preparation of the soil, banana seedling, strain Sy2-11 spore suspension, and 10-HCA solution (100 μM) followed previously described methods. 100 μM of 10-HCA was added to the soil every 2 weeks, with each seedling irrigated with 20 mL for 2 months. Plant disease incidence, chlorophyll content, leaf area, stem diameter, plant height, dry weight, and fresh weight were recorded for each treatment throughout the experiment as described above. To monitor the microbial community’s response to 10-HCA, rhizosphere soil was collected for qPCR analysis of *Foc* TR4 and 16S rRNA gene amplicon sequencing.

### Designing and construction of SynComs for disease suppression

To evaluate the disease suppression effects of *Bacillus* isolates, we meticulously designed an experiment involving the reintroduction of a SynComs composed of the aforementioned *Bacillus* sp. into sterile soil. We selected sixteen unique *Bacillus* strains from rhizosphere soil isolates of the PST group, including four ASVs of ASV7217, ASV0031, ASV0024, and ASV0350. To investigate the pair interaction between these strains, i.e., antagonism or cooperation, the growth promotion of the isolates in the supernatant of other strains was compared via metabolic cross-feeding according to the method described by ref. ^75^. In brief, the isolates were cultured in TSA liquid media for 48 hours, fermentation broth was centrifuged to remove living cells. The strain was cultured at 30 °C for 24 h and then adjusted to a cell density of OD 0.5. Subsequently, 20 μL of the supernatant from one isolate and 2 μL of the overnight cultures from the other isolates were added to 180 μL of sterile TSA liquid medium, and the mixture was incubated at 180 rpm and 30 °C. The OD was measured at 600 nm after 24 hours. The type of paired interaction between the two species was determined by calculating the ratio of the OD value of supernatant-treated isolates (Bac+supernatant) to that of the single isolate (Bac). Four isolates (Bac155, Bac147, Bac21, Bac58) were ultimately selected to construct SynComs according to the principle of non-antagonism between pairwise interactions.

The soil was subjected to autoclaving twice to ensure the elimination of all indigenous soil microbes. The effectiveness of the sterilization process was confirmed by the absence of colony growth on LB agar when soil samples were plated. Banana seedlings cultivated under sterile conditions were then transplanted into the sterilized soil in polypropylene pots. Subsequently, a spore suspension of *Foc* TR4 (1 × 10^6^ CFU/g soil) was introduced into the soil. We established three treatments: (1) Control with sterile water (CTL), (2) SynCom, (3) Strain Sy2-11 treatment group (PST) with bananas planted in natural soil.

All strains were cultured in 250 mL shake flasks containing 100 mL of LB medium, incubated at 180 rpm and 30 °C for 24 h, and then diluted with sterile water to achieve a cell density of OD 0.01. Each strain was mixed in equal proportions, and the final concentration was adjusted to 1 × 10^7^ CFU/mL. Subsequently, 100 mL of the SynCom mixture was added to inoculate the soil at a concentration of 1 × 10^6^ CFU/g soil. The experiment was performed in a randomized complete block with three biological replicates, each containing thirty pots.

### RNA-seq sequencing and data analysis of *B. velezensis*

To investigate the chemotactic mechanism of *Bacillus* sp. isolates towards 10-HCA, we performed RNA-seq sequencing of *B. velezensis* (Bac155) with 10-HCA treated. Briefly, the culture medium of strain Bac155 was collected at different time points (0 h, 0.5 h, 1 h) after 10-HCA treated. The samples were promptly frozen in liquid nitrogen and subsequently stored at a temperature of –80 °C for future analysis and experimentation. Total RNA was extracted, and genomic DNA was removed using the RNA extraction kit (TIANGEN, Beijing, China). RNA integrity and concentration were detected using 1.2% agarose gel electrophoresis and NanoDrop One (Thermo, Wilmington, America). Each cDNA library was constructed using 3 μg RNA and sequenced by the Illumina NovaSeq X Plus platform (Majorbio Bio-Pharm Technology Co., Ltd., Shanghai, China). Raw sequencing data were detected using the FastQC (v0.11.9) software to calculate the Q20, Q30, and GC content. The trimmomatic software (v0.39) was utilized for the removal of adapters and low-quality reads. The clean reads were then aligned with the reference genome of *B. velezensis* using Bowtie2 (v2.5.4). Gene expression levels were quantified with FPKM (expected number of fragments per kilobase of transcript sequence per million base pairs sequenced) using StringTie (v2.2.1). Differential expression analysis was performed to identify DEGs among different treatments. The DESeq2 R package (v1.32.0) was employed for this purpose. The DEGs were identified based on the following criteria: a FC ≥ 2 and an adjusted p value < 0.05. Protein function annotation was conducted using the eggNOG-mapper (v2.1.9) against the reference database.

### Mutants construction of *B. velezensis*

Gene deletion mutants of *cheB*, *cheC*, *cheD*, *cheF*, and *cheW* were constructed in *B. velezensis* using the suicide vector pK18mobsacB through homologous recombination. Briefly, the genomic DNA of *B. velezensis* served as the template for PCR amplification. The upstream and downstream fragments of each target gene were amplified using primer pairs *cheB*-UF/UR and *cheB*-DF/DR, respectively (Supplementary Table 3). The purified fragments were ligated into the linearized suicide plasmid pK18mobsacB (digested with BamHI and HindIII). The recombinant plasmids were first transformed into Escherichia coli DH5α, and positive clones were confirmed by PCR and sequencing. Verified constructs were subsequently introduced into the helper strain E. coli S17-1, which mediated plasmid transfer into *B. velezensis* via conjugation. Candidate mutants were selected on NA plates supplemented with kanamycin sulfate (50 μg/mL) and ampicillin (50 μg/mL), and validated by PCR and sequencing. To exercise the integrated suicide vector, potential mutants were subjected to sucrose counter-selection on NA medium containing 10% (w/v) sucrose. Final deletion mutants were confirmed by PCR using primer pair *cheB*-UF/*cheB*-DR and sequencing, and the confirmed *cheB* deletion mutant was designated *ΔcheB*. Other target gene mutants were constructed in the same manner using the respective primers.

### In situ validation of the effect of *the ΔcheW mutant* on BFW incidence

To further examine the chemotactic behavior and disease-suppressive activity of the *ΔcheW* mutant strain in soil, we performed in situ validation using a compartmentalized rhizobox system according to the method described by ref. ^76^. The compartmentalized rhizobox system comprised Compartment A and Compartment B. The soil was subjected to autoclaving twice to ensure the elimination of all indigenous soil microbes. Banana seedlings cultivated under sterile conditions were then transplanted into the sterilized soil in the compartmentalized rhizobox system. Each banana seedling was irrigated with a 100 μM 10-HCA solution at a volume of 20 mL, while the *ΔcheW* mutant strain and Bac155 strain were separately inoculated into Compartment B. The experimental design included three treatments: (1) CTL: control treatment consisted of compartment B treated with sterilized water; (2) T*ΔcheW*: compartment B inoculated with *ΔcheW* mutant strain; (3) TBac155: compartment B inoculated with strain Bac155. Each treatment was conducted using 90 plants in three replicates (30 plants per replicate). Plant disease incidence and the population densities of *Foc* TR4 were recorded for each treatment throughout the experiment as described above.

### Mutual interaction testing between strain Sy2-11 and *Bacillus* isolates

To further explore the potentially synergistic interactions between strain Sy2-11 and *Bacillus* isolates, we performed population and swimming plate assays on TSA semisolid media plates (0.5% agar, w/v) according to the method described by ref. ^54^. Briefly, strain Sy2-11 and *Bacillus* isolates were cultivated in YE and LB liquid medium for growing to an OD_600_ of approximately 0.6, respectively. To investigate the dispersal of strain Sy2-11 spores by *Bacillus* isolates, 3 μL of *Bacillus* isolates cells were inoculated onto the plate, followed by the addition of 3 μL of strain Sy2-11 spore stocks either at the *Bacillus* isolates inoculation site or at a separate site. The plates were then incubated at 30 °C for 5 days.

### Statistical analysis

All statistical analyses were performed using R (v4.2.2, http://www.r-project.org/). A One-way analysis of variance (ANOVA), followed by Fisher’s least significant difference (LSD) test with Bonferroni correction for multiple comparisons, was used to identify significant differences among the groups. The threshold for statistical significance was established at *P* < 0.05. Data are presented as mean ± MSE from independent biological replicates.

### Reporting summary

Further information on research design is available in the Nature Portfolio Reporting Summary linked to this article.

## Supplementary information

Supplementary Information

Description of Additional Supplementary Files

Supplementary Data 1-4

Reporting Summary

Transparent Peer Review file

## Source data

Source Data

## Data Availability

The data generated in this study are provided in the Supplementary Information/Source Data file. The amplicon sequencing data and transcriptome data generated in this study have been deposited in the GenBank database under accession code PRJNA1203960. Metabolomics datasets have been provided as part of the Supplementary Materials (Source data) for this manuscript. The partial 16S rRNA gene sequences of the bacterial isolates have been deposited in the GenBank database under accession codes PQ813662 - PQ814106. Source data are provided with this paper.
