# Supplementary material for: Streptomyces sesquiterpenes elicit 10-HCA secretion and recruit disease-suppressive microbiota to enhance banana Fusarium wilt resistance
Source: Nat Commun. 2026 Jun 3;17:7138. doi: 10.1038/s41467-026-73928-x (PMC13396392; doi:10.1038/s41467-026-73928-x)
Supplement: Supplementary file 1 — Supplementary Information [file 41467_2026_73928_MOESM1_ESM.docx]

**Supplementary** **Information for**

***Streptomyces* sesquiterpenes elicit 10-HCA secretion and recruit disease-suppressive microbiota to enhance banana *Fusarium* wilt resistance**

Yufeng Chen^1†^, Junting Feng^1†^, Peitao Lü^1^, Dengbo Zhou^1^, Yongzan Wei^1^, Tao Jing^1^, Zai Zheng^1^, Raza Waseem^1^, Dengfeng Qi^1^, Miaoyi Zhang^1^, Yankun Zhao^1^, Kai Li^1^, Wei Wang^1^^*^, Xu Cheng^2*^, Jianghui Xie^1*^

^1^ State Key Laboratory of Tropical Crop Breeding, Institute of Tropical Bioscience and Biotechnology & Sanya Research Institute, Chinese Academy of Tropical Agricultural Sciences, Haikou, 571101, China

^2^ Shenzhen Branch, Guangdong Laboratory of Lingnan Modern Agriculture, Key Laboratory of Synthetic Biology, Ministry of Agriculture and Rural Affairs, Agricultural Genomics Institute at Shenzhen, Chinese Academy of Agricultural Sciences, 518120, Shenzhen, China

^†^ These authors have contributed equally to this work

^*^ Corresponding author: [xiejianghui@itbb.org.cn](mailto:xiejianghui@itbb.org.cn); [chengxu@caas.cn](mailto:chengxu@caas.cn); [wangwei@itbb.org.cn](mailto:wangwei@itbb.org.cn)

**Supplementary** **Figures**


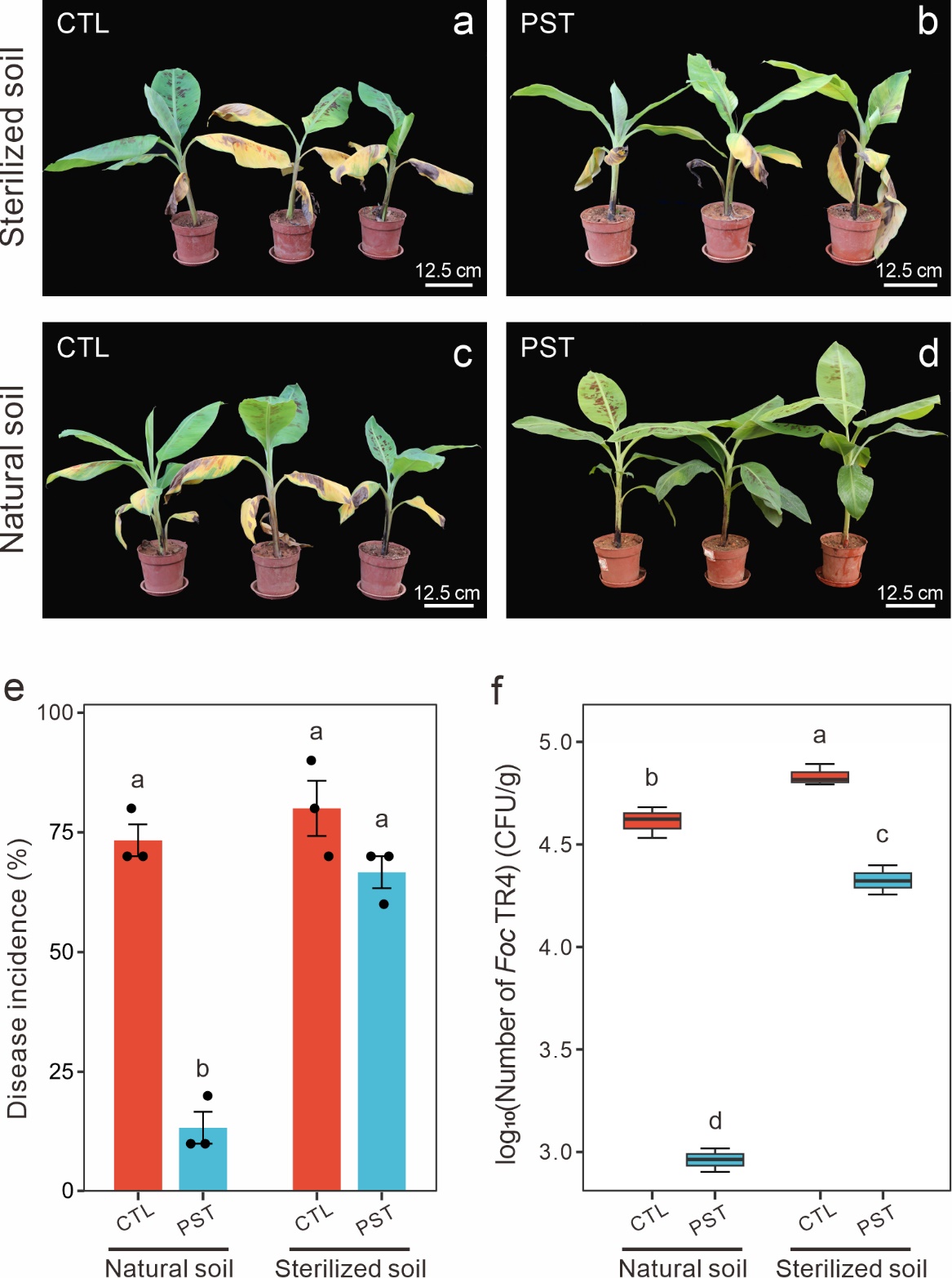


**Supplementary Fig. 1.** **Strain Sy2-11 reduces the incidence of banana *Fusarium* wilt (BFW) by driving microbial reassembly.** (**a, b, c, d**) represent the CTL and PST groups in sterilized and natural soil, respectively. (**e**) A comparison of the disease incidence (%) in the natural soil (CTL and PST groups) and sterile soil (CTL and PST groups). Data are presented as mean values ± SEM (n=3) (**f**) A comparison of *Foc* TR4 content in the natural soil (CTL and PST groups) and sterile soil (CTL and PST groups). Horizontal bars within boxes represent the median. The tops and bottoms of boxes represent 75th and 25th quartiles, respectively. The upper and lower whiskers represent the range of non-outlier data values (n=6). CTL: control treatment consisted of plants treated with sterilized water; PST: plants inoculated with strain Sy2-11. Statistical significance was assessed using LSD multiple comparison tests, with different letters indicating significant differences (*P* < 0.05).


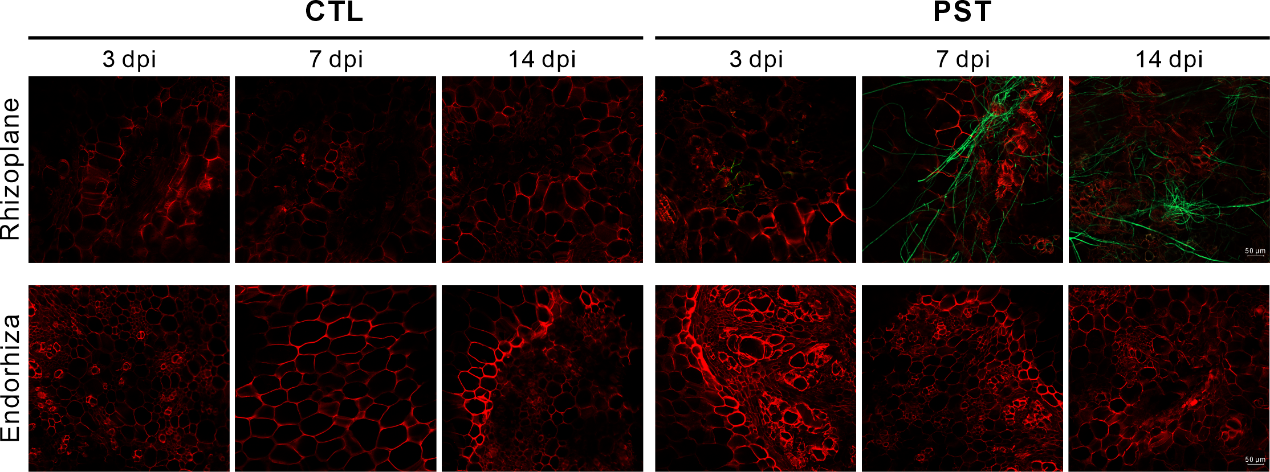


**Supplementary Fig. 2****.** **Colonization of banana** **rhizoplane and endorhiza by strain Sy2-11**. Roots from uninoculated plants (CTL) and plants inoculated with strain Sy2-11 (PST) were observed using confocal laser scanning microscopy (CLSM) at 3, 7, and 14 days post-inoculation (dpi). Green: *Foc* TR4, red: the background color of plants.


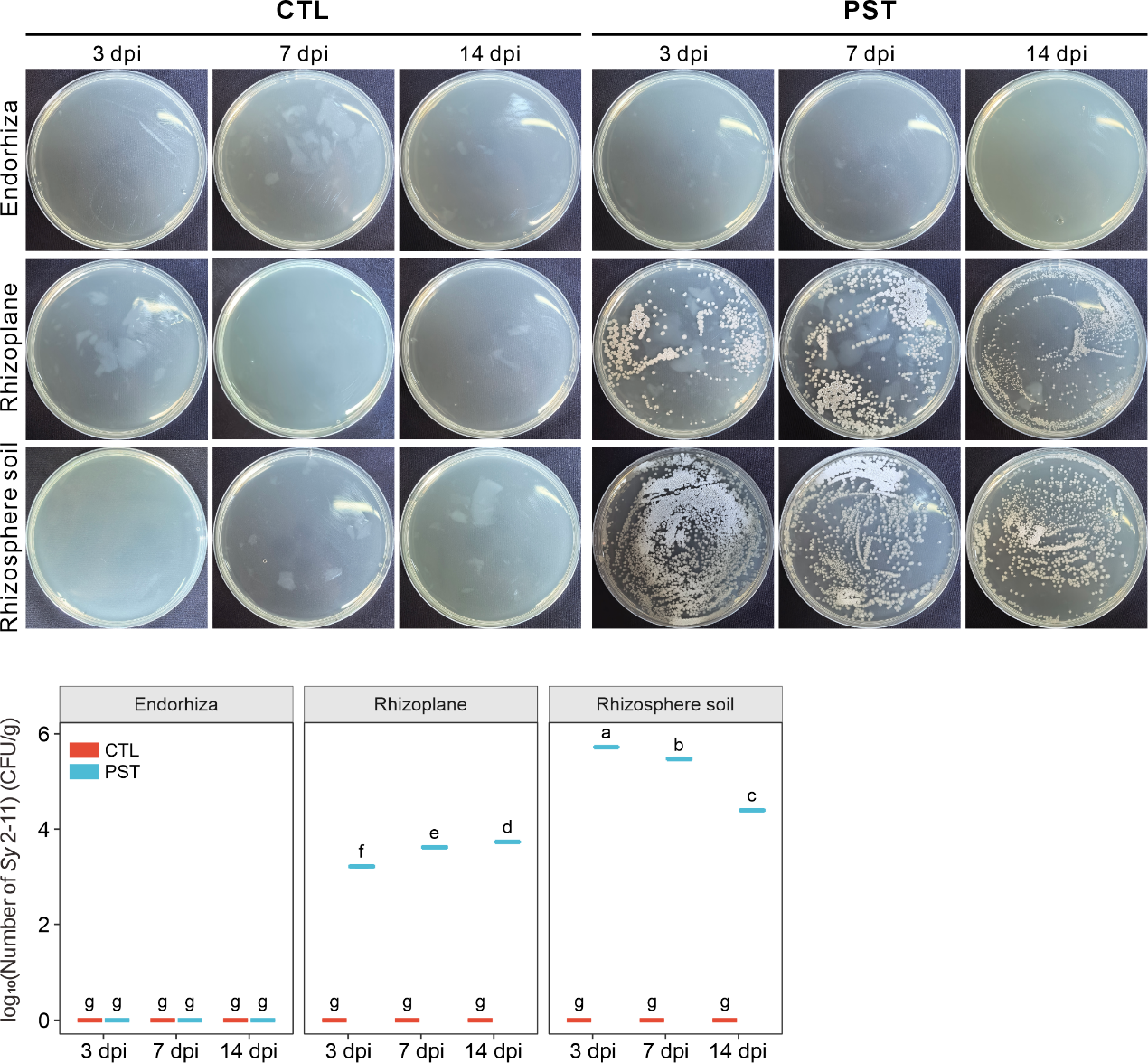


**Supplementary Fig. 3.** **Colonization dynamics of strain Sy2-11 to colonize the banana rhizosphere soil,** **rhizoplane, and endorhiza.** Soils and roots from uninoculated plants (CTL) and plants inoculated with strain Sy2-11 (PST) were used to quantify their population at 3, 7, and 14 days post-inoculation (dpi). Data are presented as mean values ± SEM. Statistical significance was assessed using LSD multiple comparison tests, with different letters indicating significant differences (*P* < 0.05, n=3).


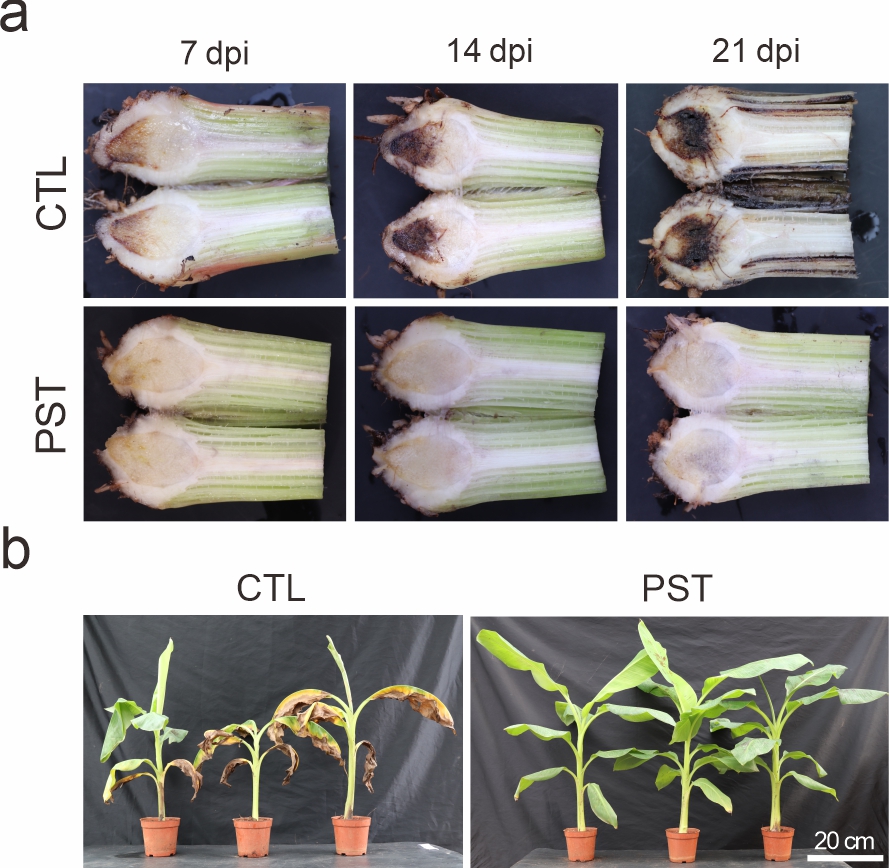


**Supplementary Fig. 4.** ***S. yongxingensis* sp. nov.** **Sy2-11 improves banana seedlings’ resistance to *Fusarium* wilt disease**. (**a**) Cross-sectional observations of banana corms following inoculation with the *Foc* TR4 in Field 2. (**b**) Phenotypic comparison of banana seedlings in the CTL and PST groups at 35 days post-inoculation (dpi) in Field 2. CTL: control treatment consisted of plants treated with sterilized water; PST: plants inoculated with strain Sy2-11.


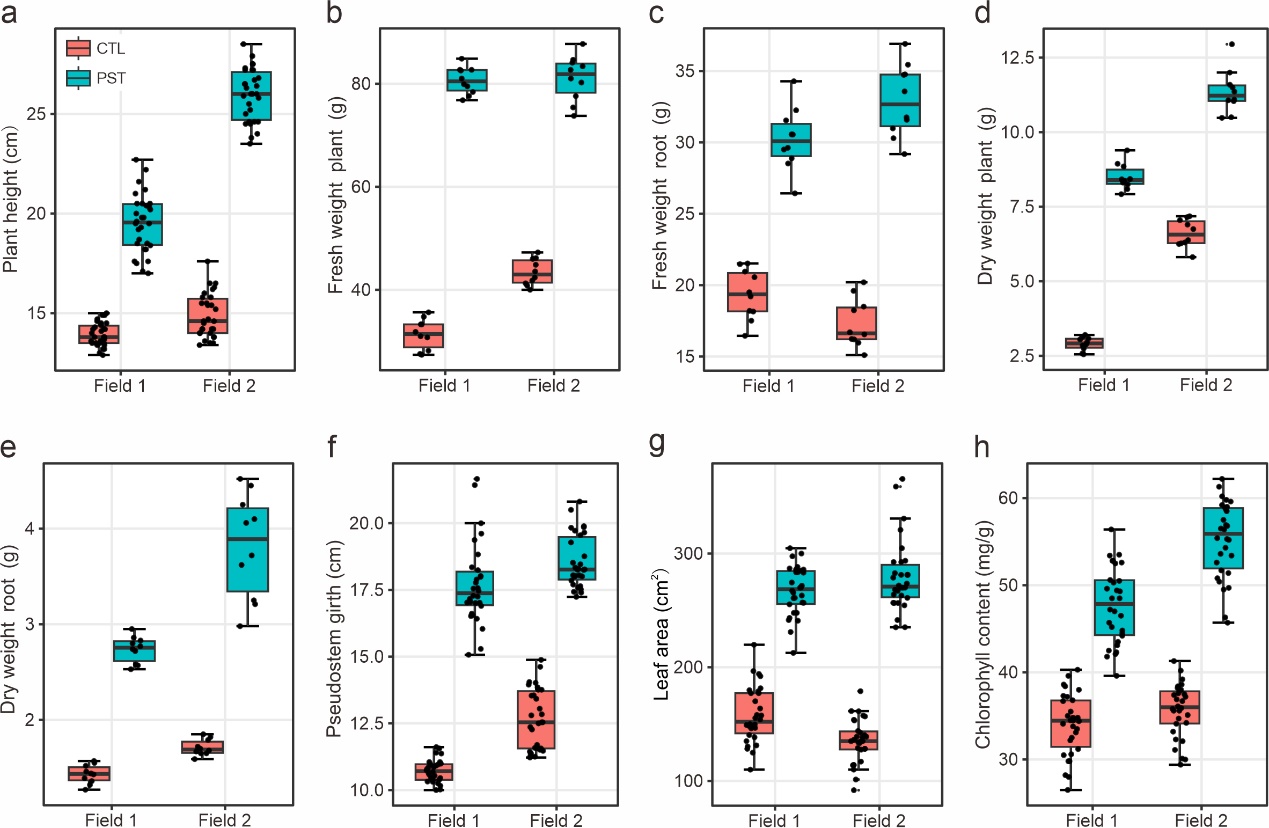


**Supplementary Fig. 5.** **Phenotypes of banana seedlings in CTL and PST groups**. (**a**) plant height, (**b**) plant fresh weight (excluding roots), (c) root fresh weight, (**d**) plant dry weight (excluding roots), (**e**) root dry weight, (**f**) pseudostem girth, (**g**) leaf area, (**h**) leaf chlorophyll content. CTL: control treatment consisted of plants treated with sterilized water; PST: plants inoculated with strain Sy2-11. Horizontal bars within boxes represent the median. The tops and bottoms of boxes represent 75th and 25th quartiles, respectively. The upper and lower whiskers represent the range of non-outlier data values (n=10).


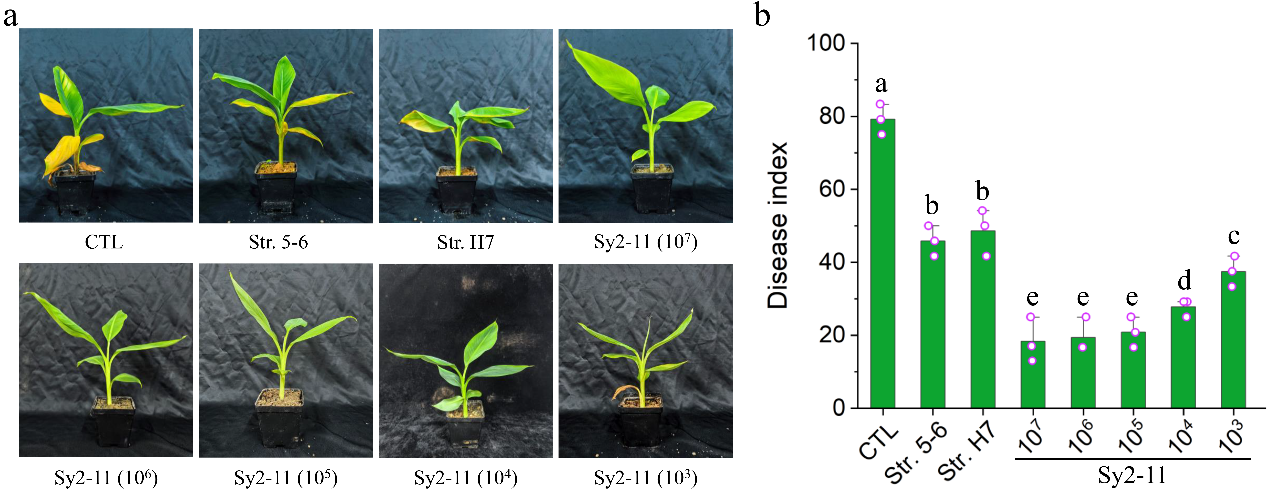


**Supplementary Fig. 6. The inhibitory effect of inoculating different concentrations of strain Sy2-11 against** **banana *Fusarium* wilt.** (a) phenotypic comparison of banana seedlings following treatment with different concentrations of strain Sy2-11. (b) Disease index comparison among inoculating different concentrations of strain Sy2-11. CTL: control treatment consisted of plants treated with sterilized water; Str. 5-6: plants inoculated with strain 5-6. Str. H7: plants inoculated with strain H7. Data are presented as mean values ± SEM (n=3). Statistical significance was assessed using LSD multiple comparison tests, with different letters indicating significant differences (*P* < 0.05, n=3).


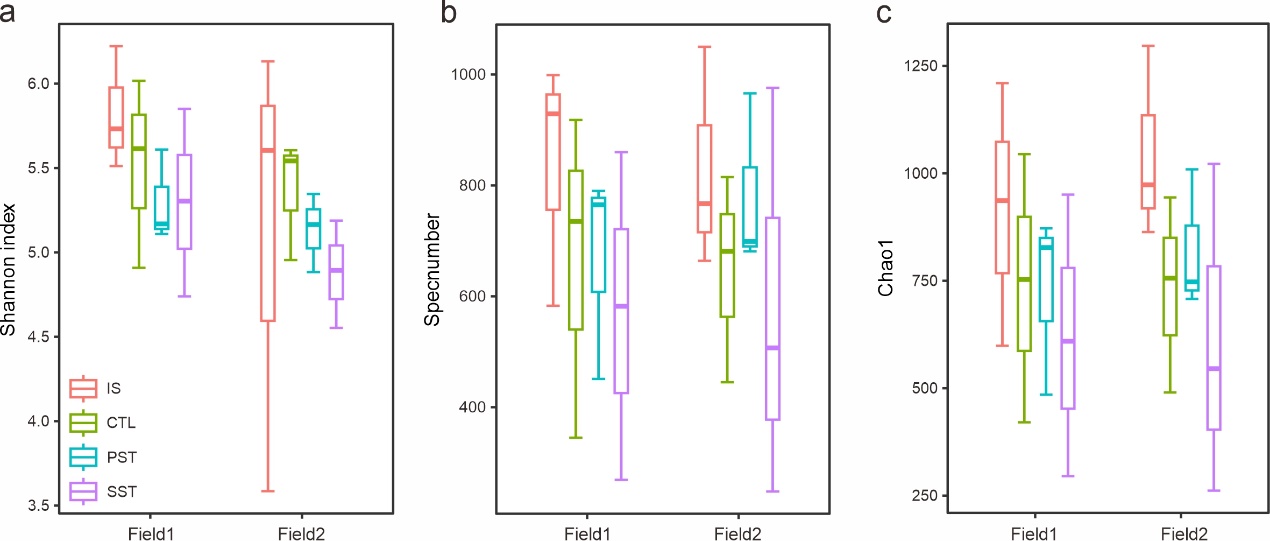


**Supplementary Fig. 7. Influence of strain Sy2-11 on alpha diversity of bacterial communities in banana rhizosphere**. (**a**) Shannon index, (**b**) Specnumber index, (**c**) Chao1 index. IS: initial soil; CTL: control treatment consisted of plants treated with sterilized water; PST: plants inoculated with strain Sy2-11; SST: soil (without plants) inoculated with strain Sy2-11.


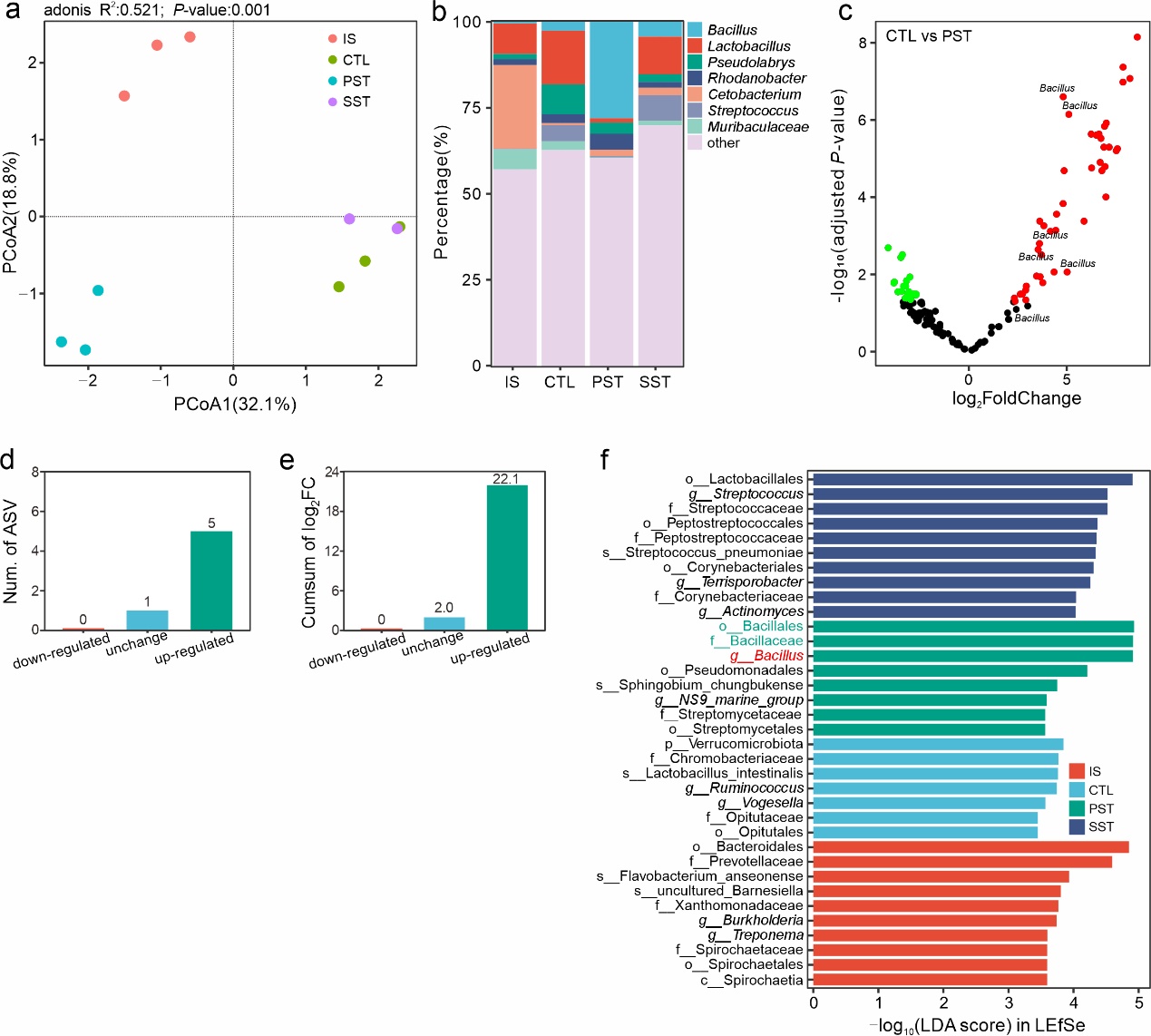


**Supplementary Fig. 8.** ***Bacillus* as the core microbiota recruited by strain Sy2-11-induced banana roots in Field 2.** (**a**) The impact of strain Sy2-11 on the *β*-diversity of bacterial communities in the banana rhizosphere. (**b**) Bar plot displaying the relative abundance of Amplicon Sequence Variants (ASVs) at the genus level across four experimental groups, highlighting the top seven genera. (**c**) Volcano plot showing a significant increase in *Bacillus* abundance in the PST group as compared to the CTL group. (**d**) The number of the *Bacillus* species that were upregulated and downregulated in response to strain Sy2-11 treatment. (**e**) Cumulative log_2_ (Fold Change) plot of *Bacillus*. (**f**) LEfSe analysis confirming *Bacillus* as the most significant biomarker in the PST group. IS: initial soil; CTL: control treatment consisted of plants treated with sterilized water; PST: plants inoculated with strain Sy2-11; SST: soil (without plants) inoculated with strain Sy2-11.


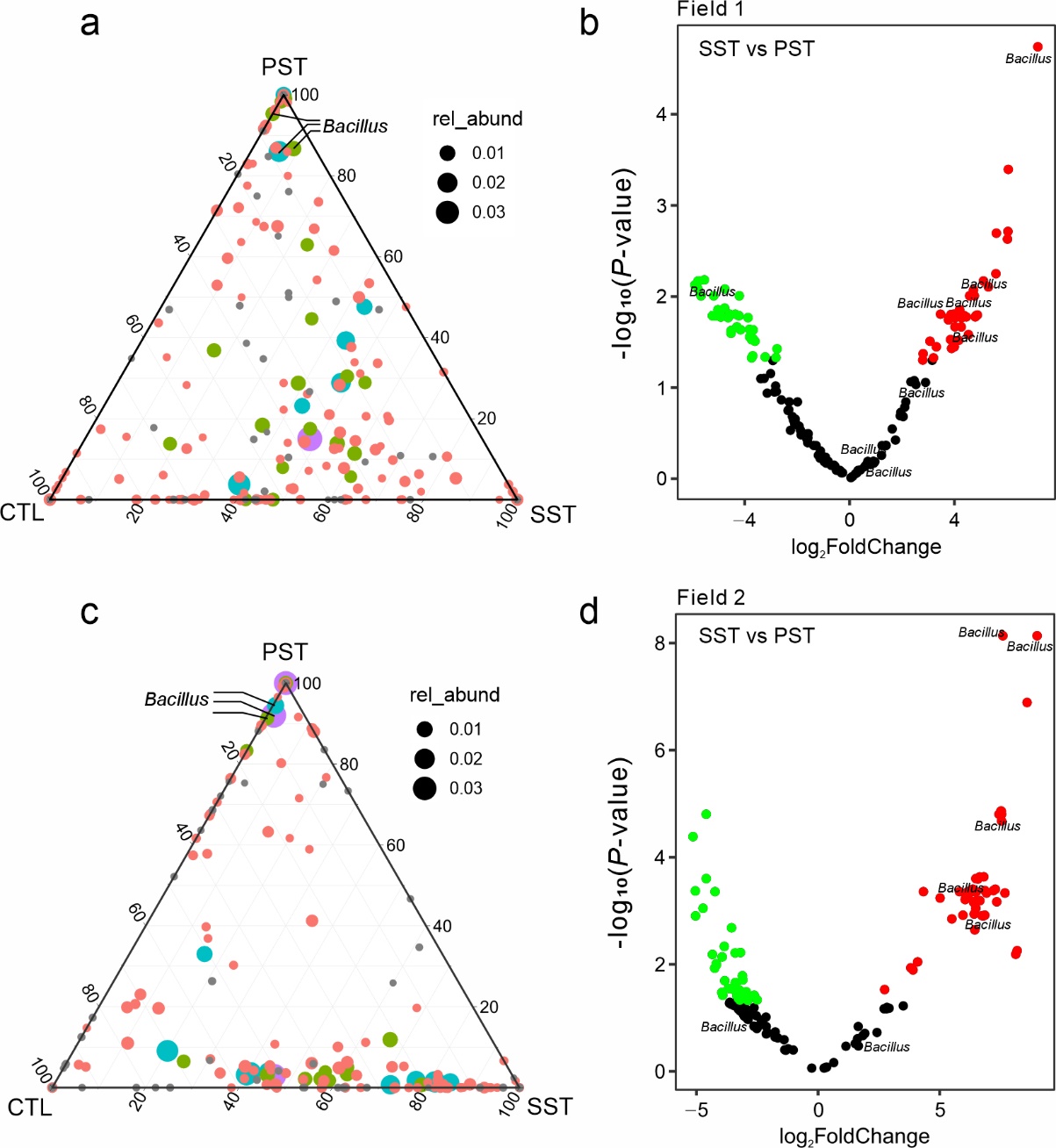


**Supplementary Fig. 9.** ***Bacillus* enrichment in PST group across Fields**. (**a**, **c**) Ternary plots demonstrate the significant enrichment of *Bacillus* in the PST group as compared to other groups in both Field 1 and Field 2, respectively. (**b**, **d**) Volcano graphs show a significant increase in the number of *Bacillus* spp. in the PST group compared to the SST group across Field 1 and Field 2, indicating the robust response of *Bacillus* to the PST in different environmental contexts. CTL: control treatment consisted of plants treated with sterilized water; PST: plants inoculated with strain Sy2-11; SST: soil (without plants) inoculated with strain Sy2-11.


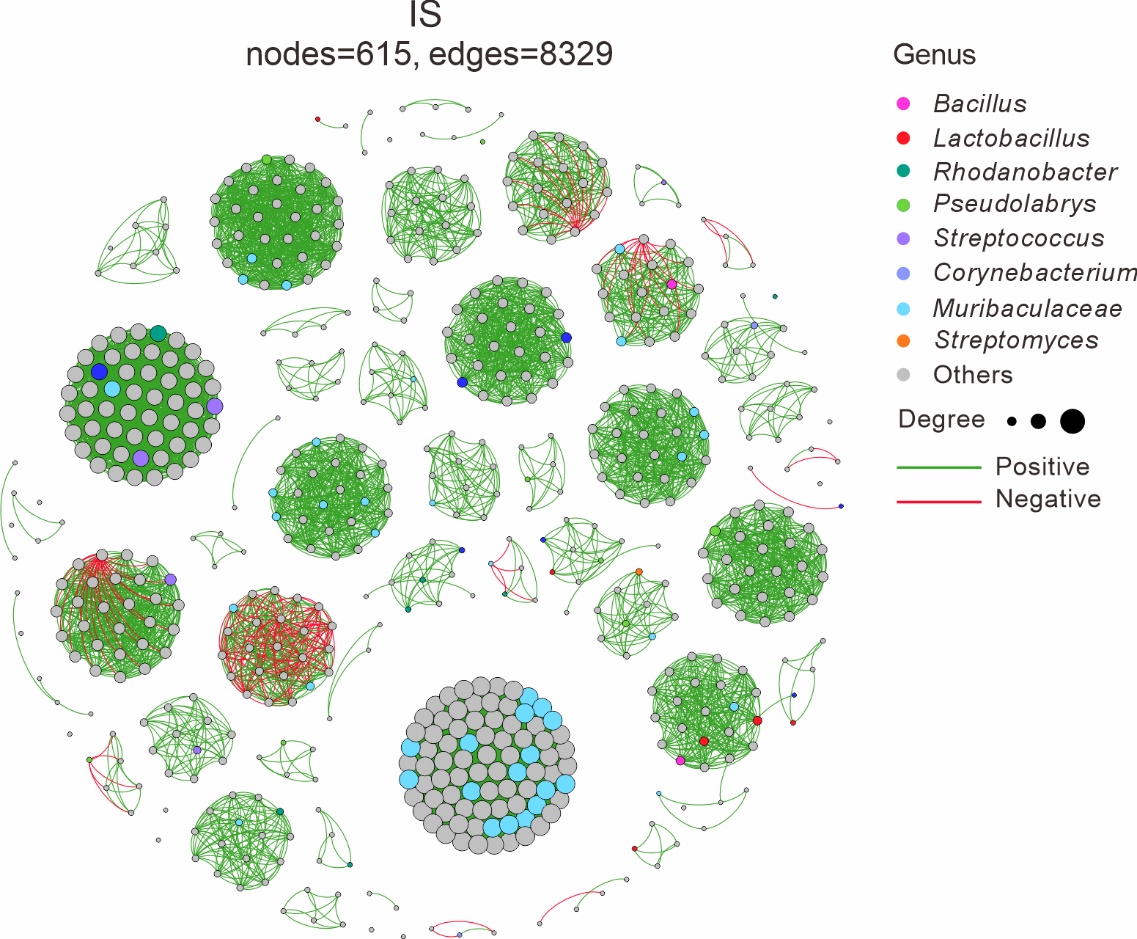


**Supplementary Fig. 10.** Microbial co-occurrence network topology of the initial soil (IS) group. Identical colors represent the same genus in (a) and (e), with pink indicating *Bacillus*.


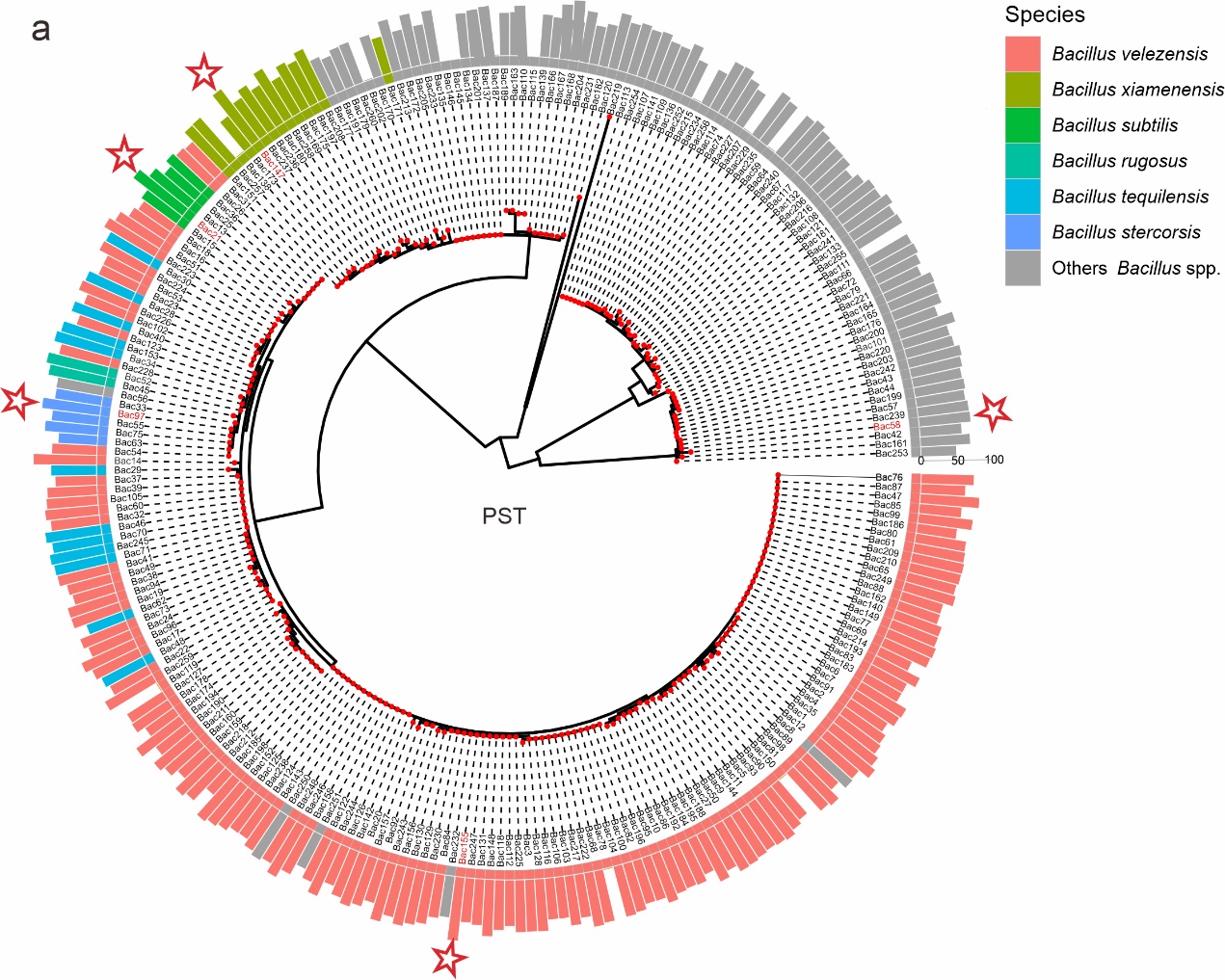


**Supplementary Fig. 11. Isolation of core *Bacillus* sp. and validation of suppression ability on *Foc* TR4 pathogen.** Maximum likelihood phylogenetic tree based on full-length 16S rRNA gene sequences of 260 *Bacillus* isolates from the PST group. The outer layer indicates the inhibition rates of the isolates against *Foc* TR4 in plate assays. PST: plants inoculated with strain Sy2-11.


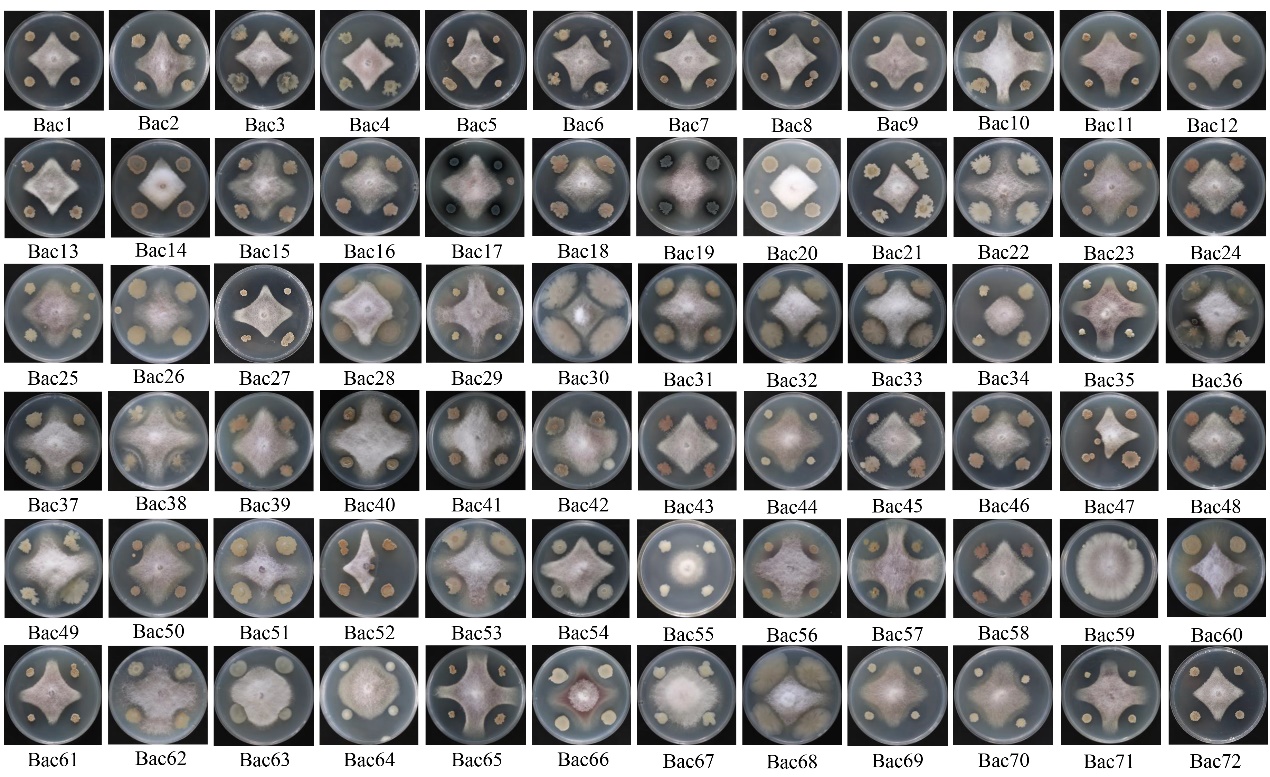


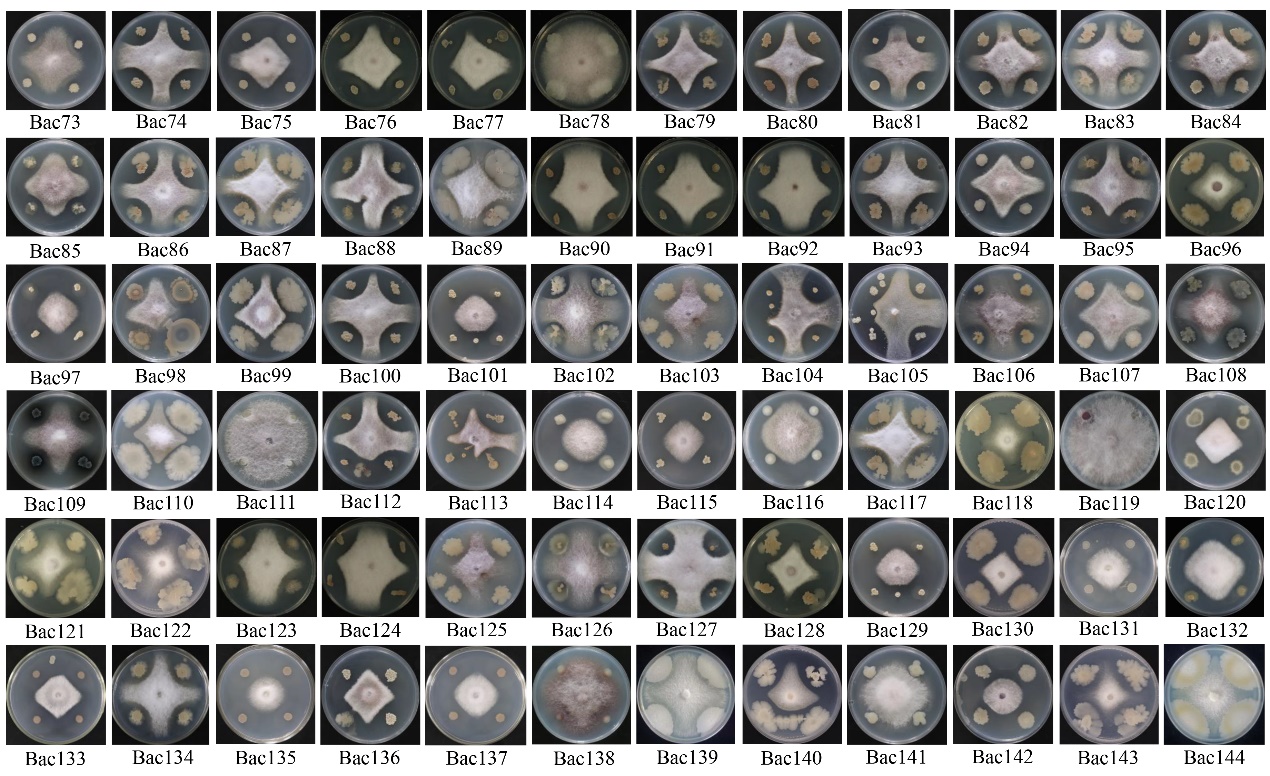


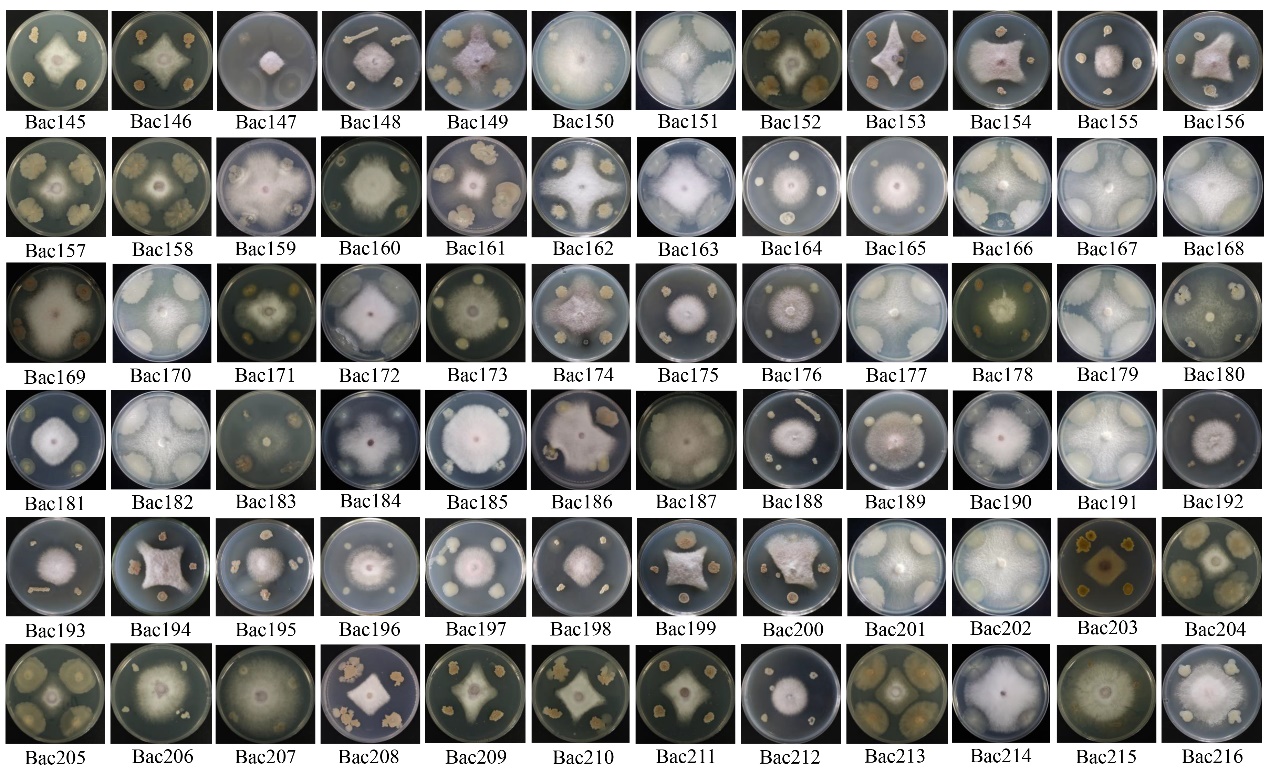


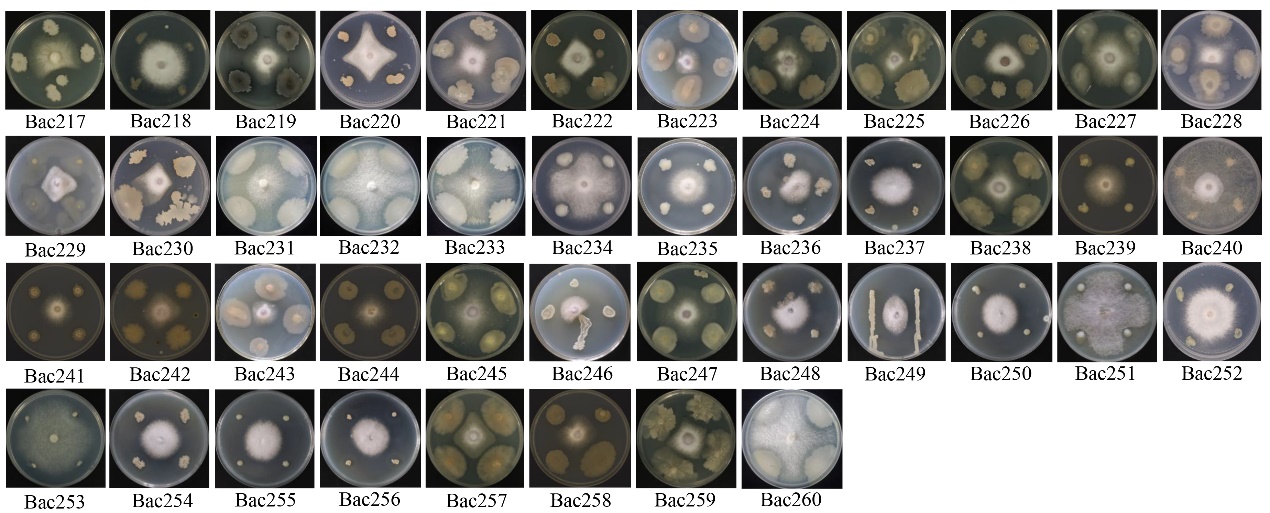


**Supplementary Fig. 12.** **Prevalence of antifungal activity among *Bacillus* isolates from the PST group**. In the PST group, a significant proportion of *Bacillus* isolates, specifically 95.6%, exhibited antifungal activities against *Foc* TR4.


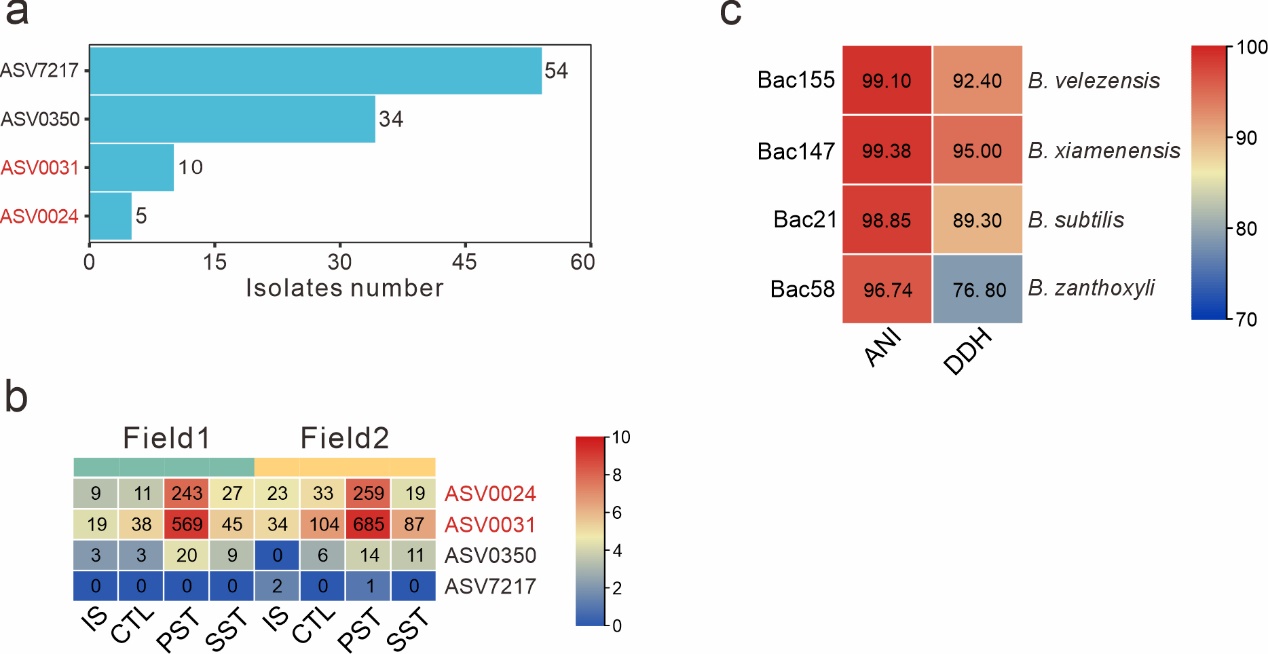


**Supplementary Fig. 13.** **Correlation analysis between isolated *Bacillus* strains and amplicon sequence variants (ASVs).** **(a)** Five ASVs corresponding to *Bacillus* isolates with 100% identity. (**b**) **The abundance of** ASVs **that matched with the isolated *Bacillus* strains. (c) Orthologous average nucleotide identity (**OrthoANI) values were calculated to assess the genetic similarity between the isolates. Additionally, digital DNA-DNA hybridization (dDDH) analysis was performed using the online tools OAT and GGDC v2.1 to compare the genomes of the isolates with closely related reference strains for the construction of SynComs and to understand their phylogenetic relationships. IS: initial soil; CTL: control treatment consisted of plants treated with sterilized water; PST: plants inoculated with strain Sy2-11; SST: soil (without plants) inoculated with strain Sy2-11.


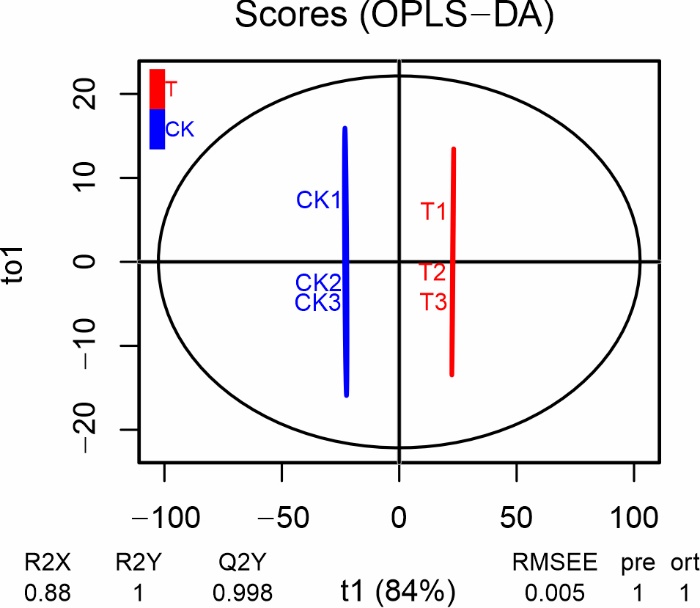


**Supplementary Fig. 14.** **Orthogonal partial least squares-discriminant analysis (OPLS-DA) of root exudates.** This plot displays the OPLS-DA of root exudates comparing the Control (CK1, CK2, and CK3) with the Treatment groups (T1, T2, and T3).


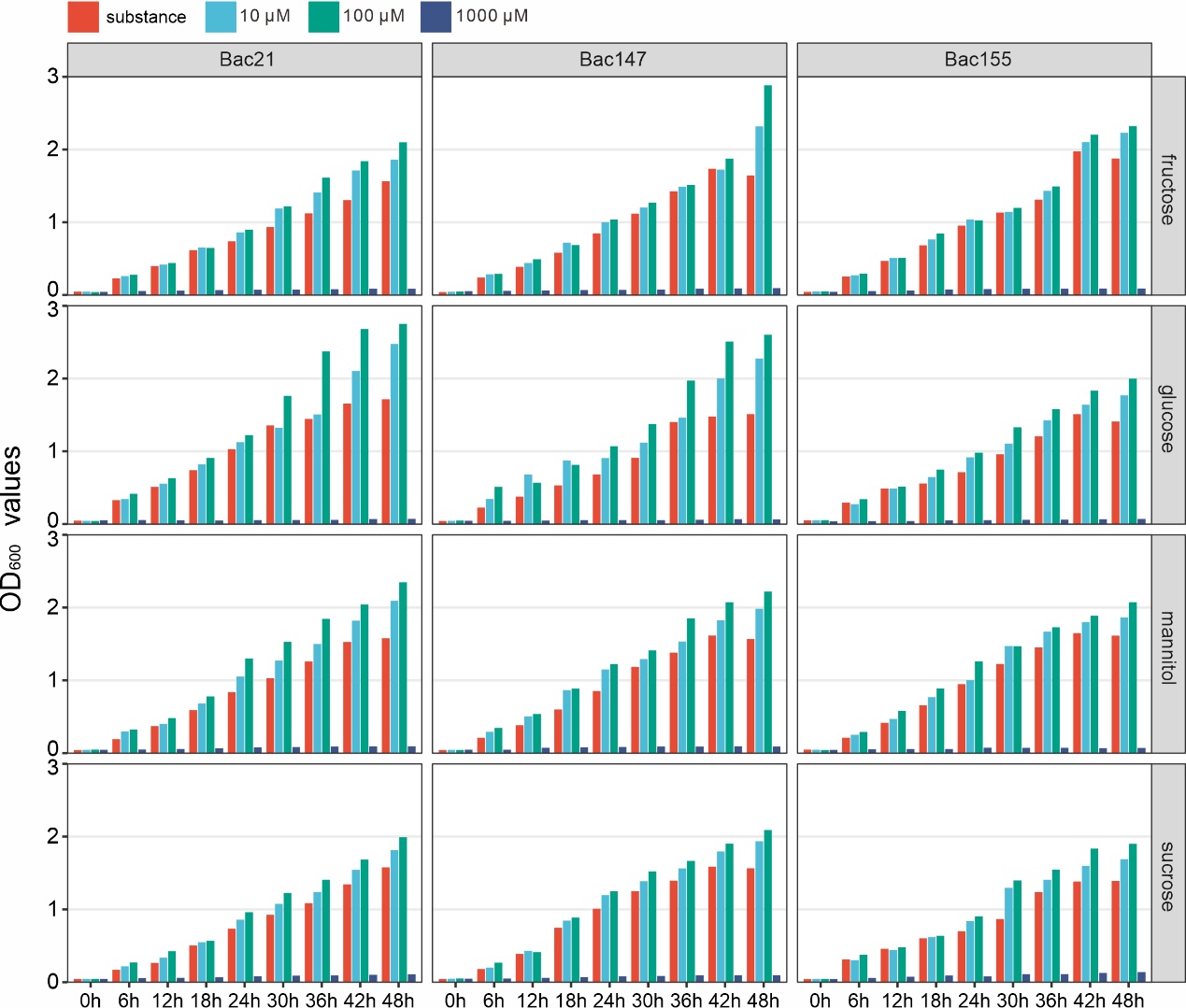


**Supplementary Fig. 15.** **Effects of 10-HCA on the growth of *Bacillus* isolates**. These plots investigate the effects of 10-HCA on the growth of *Bacillus* isolates, specifically Bac155, Bac147, and Bac21, in inorganic salt media. The media were supplemented with various carbon sources, including D-fructose, glucose, D-mannitol, and sucrose, which are indicated in red. Additionally, the media were amended with different concentrations of 10-HCA: 10 μM (light blue), 100 μM (green), and 1000 μM (dark blue), to assess the influence of 10-HCA on bacterial growth under varying conditions.


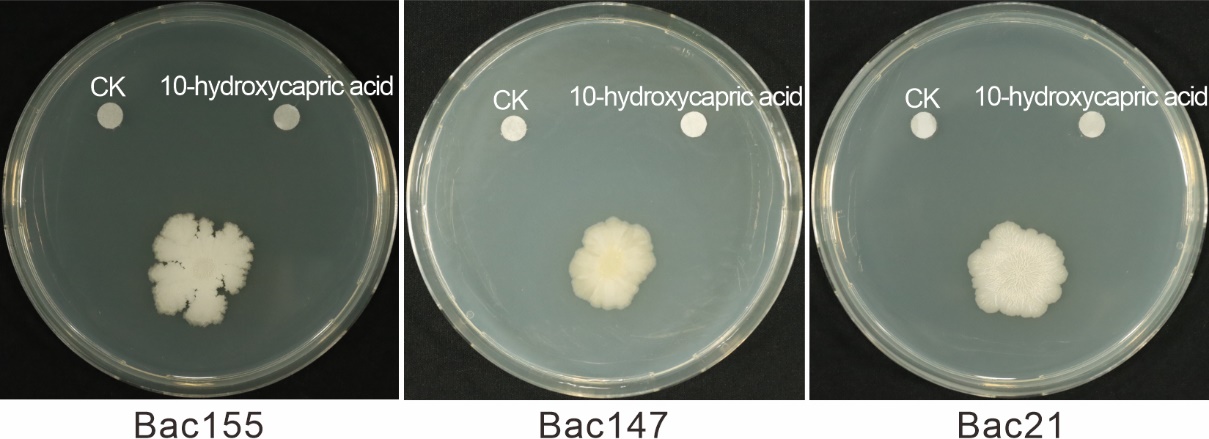


**Supplementary Fig. 16.** **Lack of significant growth of disease-suppressing *Bacillus* strains toward 10-hydroxydecanoic acid.** The images demonstrate that the *Bacillus* strains known for their disease-suppressing properties did not show substantial growth when exposed to 10-hydroxydecanoic acid.


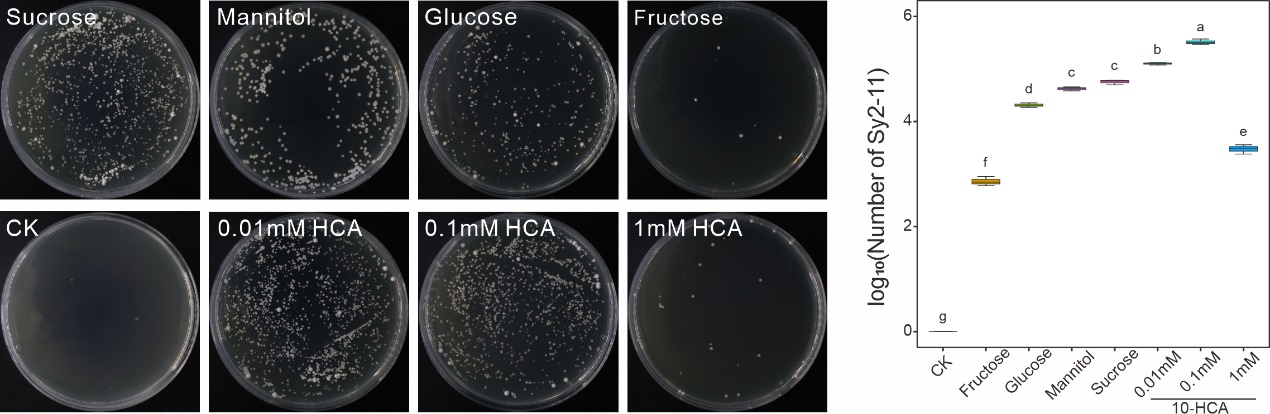


**Supplementary Fig. 17.** **Effects of 10-HCA on the growth of strain Sy2-11.** These plots illustrate the effects of 10-HCA on the growth of strain Sy2-11 in inorganic salt media. The media were supplemented with various carbon sources, including D-fructose, glucose, D-mannitol, and sucrose. Furthermore, the media were amended with 10‑HCA at three concentrations (10 μM, 100 μM, and 1000 μM) to evaluate its influence on the growth of strain Sy2‑11 under these different conditions. Horizontal bars within boxes represent the median. The tops and bottoms of boxes represent 75th and 25th quartiles, respectively. The upper and lower whiskers represent the range of non-outlier data values. Statistical significance was assessed using LSD multiple comparison tests, with different letters indicating significant differences (*P* < 0.05, n=3)


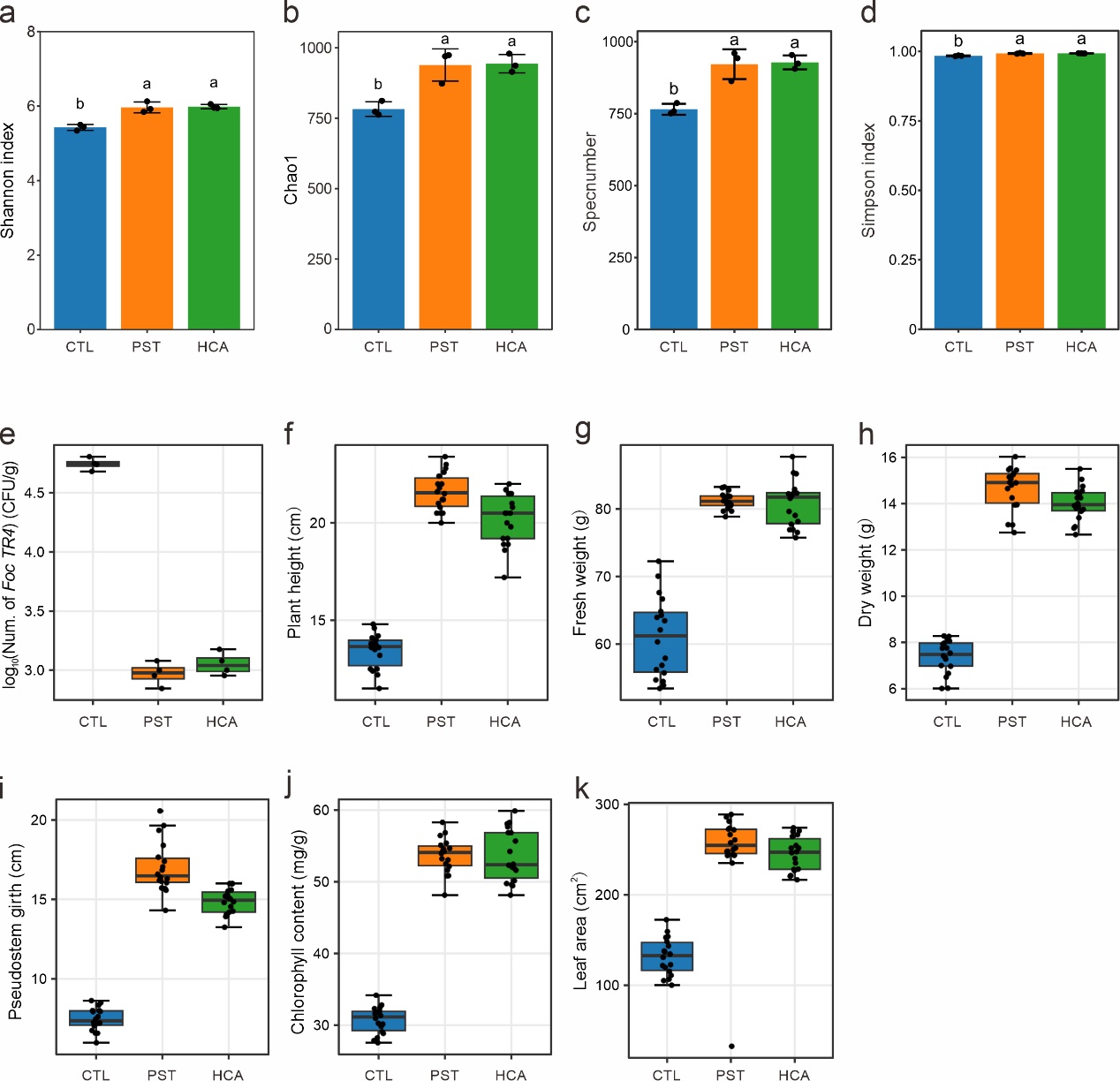


**Supplementary Fig.** **18.** **10-hydroxycapric acid induces microbiome-directed assembly and reduces the incidence of banana wilt disease.** The HCA and the PST groups have similar alpha diversity values, as measured by the following indices: (**a**) Shannon index, (**b**) Chao1, (**c**) Specnumber, and (**d**) Simpson index, both groups show significantly different diversity values compared to the CTL group. (**e**) The *Foc* TR4 content across the CTL, PST, and HCA groups. The phenotypes of banana seedlings across the CTL, PST, and HCA groups are compared for various growth parameters: (**f**) plant height, (**g**) fresh weight, (**h**) dry weight, (**i**) pseudostem girth, (**j**) leaf chlorophyll content, and (**k**) leaf area. CTL: control treatment consisted of plants treated with sterilized water; PST: plants inoculated with strain Sy2-11; HCA: plants inoculated with 10-HCA. In (a)-(d), data are presented as mean values ± SEM. Statistical significance was assessed using LSD multiple comparison tests, with different letters indicating significant differences (*P* < 0.05, n=3). In (e)-(k), horizontal bars within boxes represent the median. The tops and bottoms of boxes represent 75th and 25th quartiles, respectively. The upper and lower whiskers represent the range of non-outlier data values (n=10).


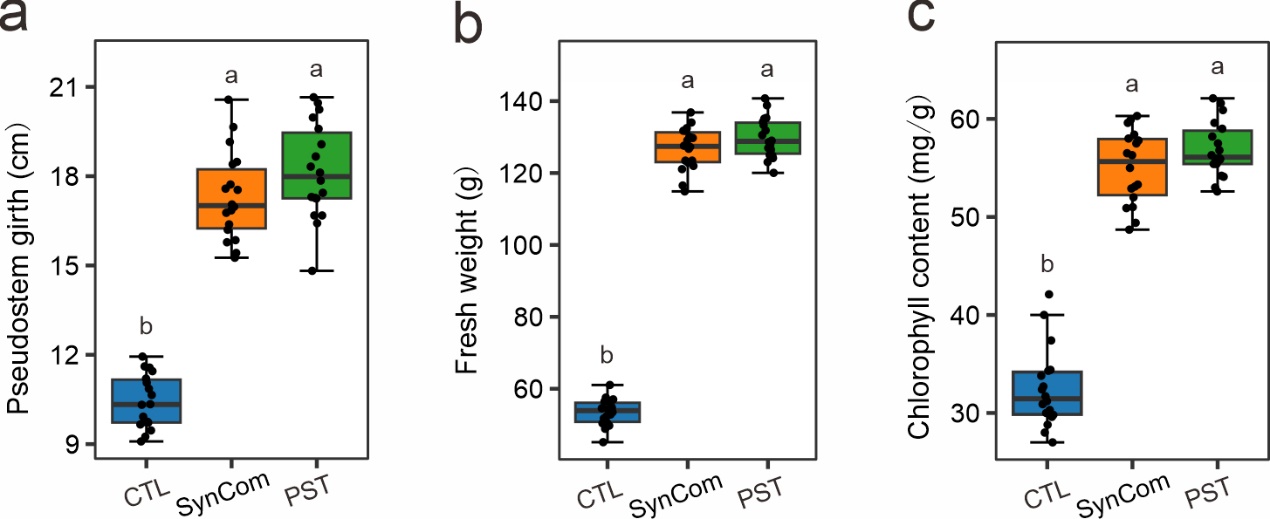


**Supplementary Fig. 19.** **Growth phenotypes of banana seedlings in CTL, SynComs, and PST groups.** The parameters measured include (**a**) pseudostem girth, (**b**) fresh weight, (**c**) leaf chlorophyll content. CTL: control treatment consisted of plants treated with sterilized water; PST: plants inoculated with strain Sy2-11; SynCom: plants inoculated with SynCom. Statistical significance was assessed using LSD multiple comparison tests, with different letters indicating significant differences (*P* < 0.05, n=10). Horizontal bars within boxes represent the median. The tops and bottoms of boxes represent 75th and 25th quartiles, respectively. The upper and lower whiskers represent the range of non-outlier data values.


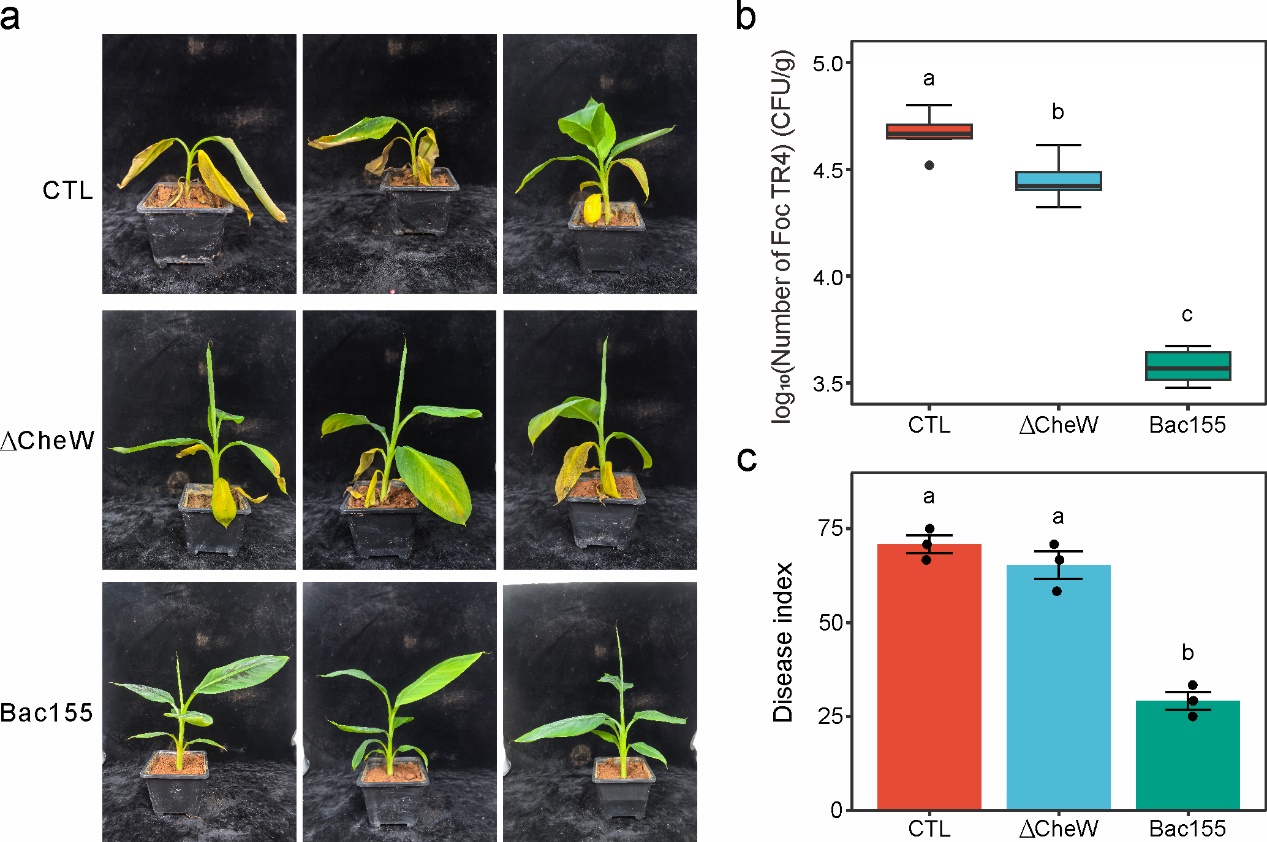


**Supplementary Fig. 20. In-situ validation of the suppression ability of strain Bac155 and its *ΔcheW* mutant against *Foc* TR4 and BFW. (a) phenotypic comparison of banana seedlings following treatment with strain Bac155 and the *ΔcheW* mutant strain. Seedlings in the Bac155 group showed healthy growth, while those in the CTL and *ΔcheW* group exhibited obvious symptoms of Fusarium wilt disease. Images were taken at 35 dpi. (b) Comparison of *Foc* TR4 content, showing a significant reduction in pathogen load in the Bac155 group.** Horizontal bars within boxes represent the median. The tops and bottoms of boxes represent 75th and 25th quartiles, respectively. The upper and lower whiskers represent the range of non-outlier data values (n=6) **(c) Disease index comparison among the CTL, *ΔcheW*, and Bac155 groups.** Data are presented as mean values ± SEM (n=3). **CTL: control treatment consisted of plants treated with sterilized water; *ΔcheW*: plants inoculated with the *ΔcheW* mutant strain; Bac155: plants inoculated with strain Bac155. Statistical significance was assessed using LSD multiple comparison tests, with different letters indicating significant differences (***p* < 0.05**).** Statistical significance was assessed using LSD multiple comparison tests, with different letters indicating significant differences (*P* < 0.05).


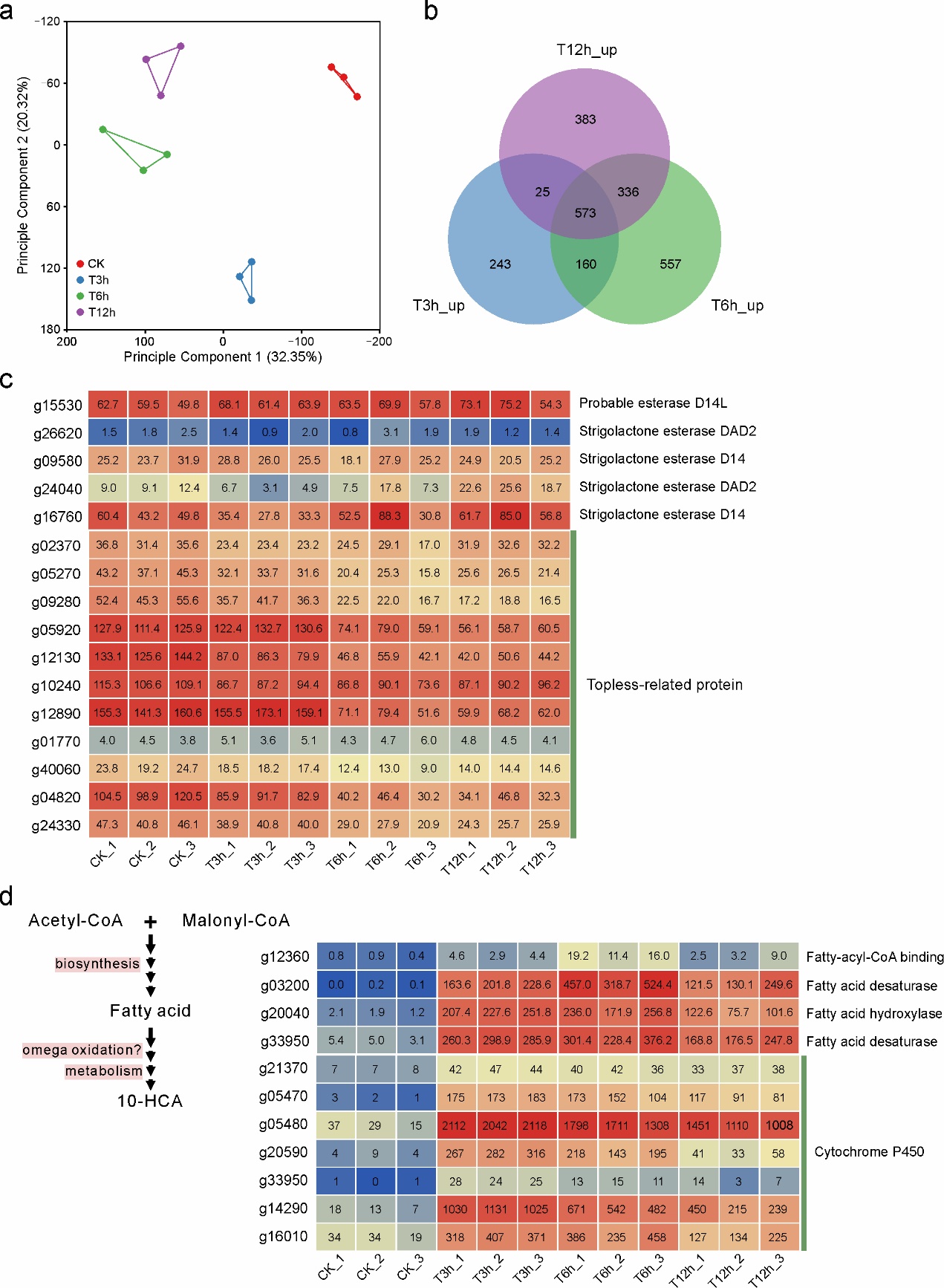


**Supplementary Fig. 21. Identification of genes involved in 10-HCA biosynthesis and regulatory genes for sesquiterpene ledene perception in banana roots.** (a) Principal component analysis (PCA) of transcriptome sequencing demonstrated excellent reproducibility among biological replicates and distinct transcriptional differences between the treatment and control groups. (b) A total of 1001, 1626, and 1317 upregulated genes were identified at 3 h, 6 h, and 12 h, respectively, with 573 genes commonly upregulated at all three time points. (c) 5 homologous genes of the KAI2 gene responsible for (-)-germacrene D perception and 11 homologous genes encoding TOPLESS-like proteins responsible for *β*-caryophyllene perception were identified in banana. (d) 11 enzymes associated with fatty acid metabolism were identified.


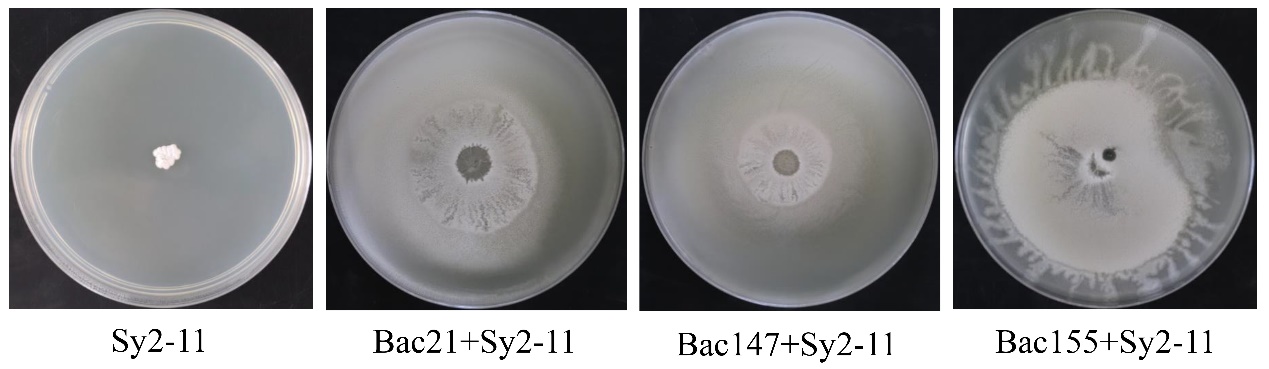


**Supplementary Fig. 22.** ***Bacillus* sp. promoted the spread of spores from strain Sy2-11.**


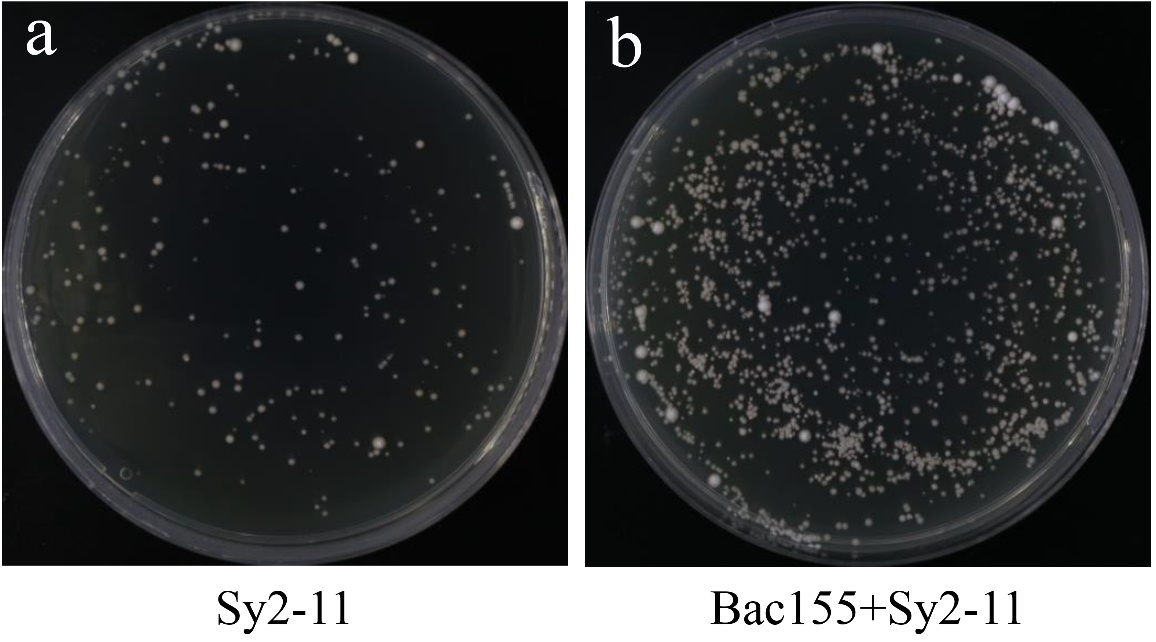


**Supplementary Fig. 23. *In situ* validation of *Bacillus*-promoted strain Sy2-11 colonization on banana roots.**


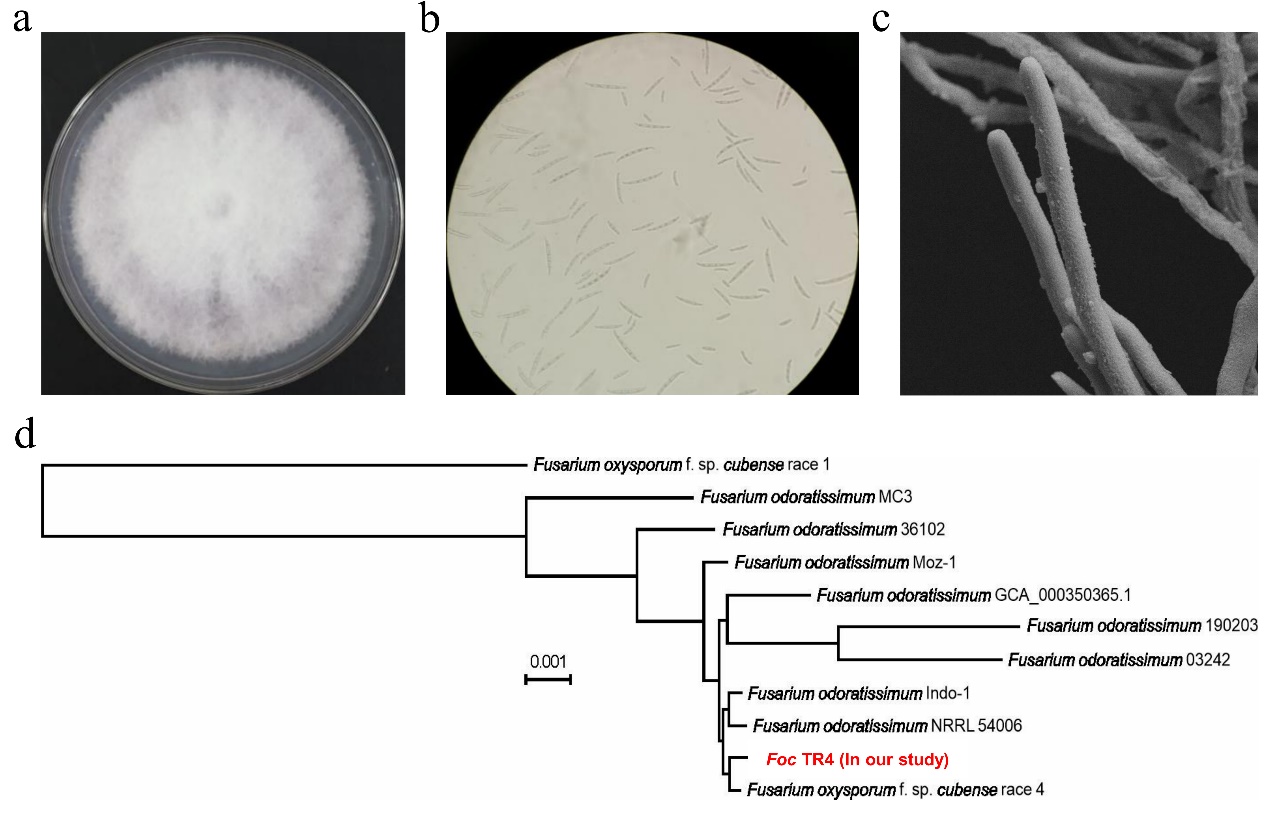


**Supplementary Fig. 24. *Fusarium oxysporum* f. sp. *cubense*** **tropical** **race 4 (*Foc* TR4, in our study).** **(a)** Culture grown on PDA. **(b)** Falcate-shaped macroconidia. **(c) Mycelium. (d) Genome‑based phylogenetic tree.**


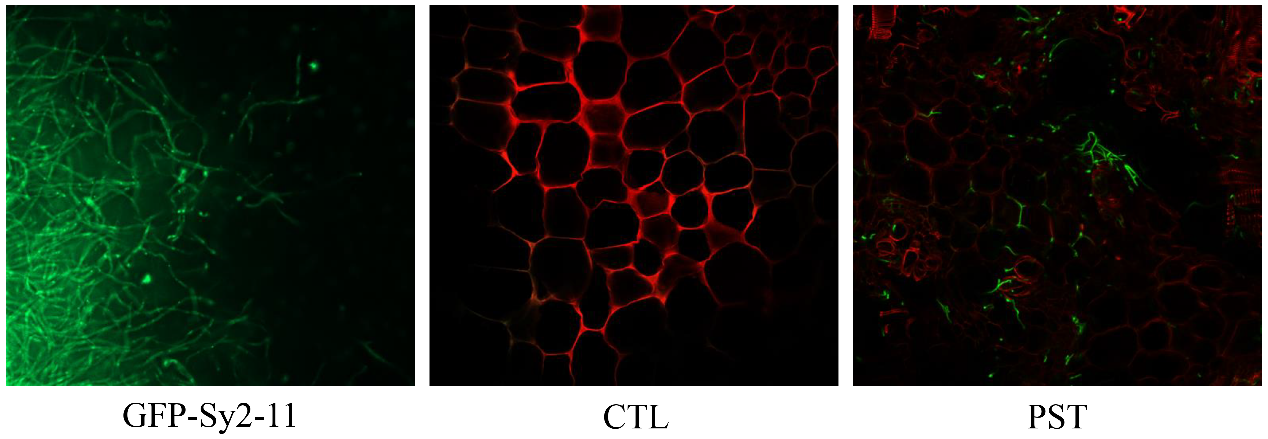


**Supplementary Fig. 25.** **Colonization of banana** **roots by strain Sy2-11**. Roots from uninoculated plants (CTL) and plants inoculated with strain Sy2-11 (PST) were observed using confocal laser scanning microscopy (CLSM) at 3 days post-inoculation (dpi). GFP-Sy2-11: GFP-tagged strain Sy2-11. Green: *Foc* TR4, red: the background color of plants.


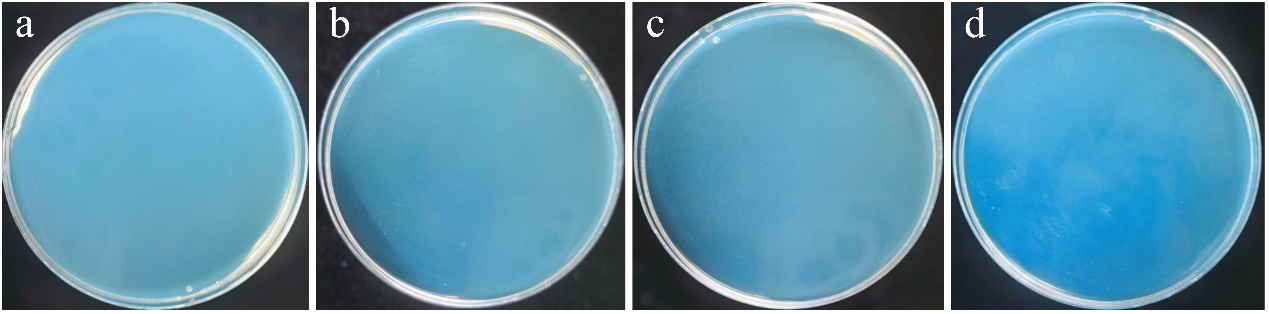


**Supplementary Fig. 26. Sterility assessment of root exudate solutions collected from the split-root system. (a) Control, treated with (b) strain Sy2-11, (c) metabolites, and (d) VOCs.**

**Supplementary Table 1. *P*-values from one-sided t-test assessing chemotactic responses of three *Bacillus* strains to different metabolite treatments** **(n=3).**

| **Num** | **Bac155** | | **Bac147** | | **Bac21** | |
| --- | --- | --- | --- | --- | --- | --- |
|  | **less0.4** | **greater0.6** | **less0.4** | **greater0.6** | **less0.4** | **greater0.6** |
| pos_337 | 0.963 | 1.000 | 0.955 | 0.997 | 0.646 | 1.000 |
| neg_178 | 0.999 | 0.999 | 0.996 | 0.998 | 0.998 | 0.999 |
| pos_205 | 0.997 | 0.999 | 0.998 | 0.999 | 0.991 | 0.997 |
| pos_175 | 1.000 | 0.008 | 0.999 | 0.093 | 0.999 | 0.296 |
| pos_330 | 0.997 | 0.649 | 1.000 | 0.853 | 0.996 | 0.960 |
| neg_106 | 0.975 | 0.981 | 0.999 | 0.996 | 0.994 | 0.992 |
| neg_49 | 0.734 | 0.999 | 0.695 | 0.996 | 0.983 | 1.000 |
| neg_10 | 0.999 | 0.017 | 1.000 | 0.796 | 0.999 | 0.053 |
| pos_231 | 0.998 | 0.369 | 0.999 | 0.999 | 1.000 | 0.995 |
| neg_32 | 1.000 | 0.000 | 1.000 | 0.003 | 1.000 | 0.002 |
| pos_517 | 0.994 | 0.999 | 0.999 | 1.000 | 0.998 | 1.000 |
| neg_173 | 0.998 | 0.997 | 0.999 | 0.999 | 0.998 | 0.998 |
| neg_6 | 0.993 | 0.958 | 0.999 | 0.998 | 1.000 | 0.999 |
| pos_490 | 0.283 | 0.997 | 0.993 | 1.000 | 0.949 | 0.998 |
| pos_464 | 0.992 | 0.999 | 0.994 | 0.999 | 0.994 | 0.999 |
| neg_30 | 0.996 | 0.974 | 0.998 | 0.996 | 0.998 | 0.919 |
| pos_439 | 0.996 | 0.997 | 0.988 | 0.972 | 1.000 | 1.000 |
| pos_315 | 0.968 | 0.992 | 0.999 | 0.999 | 0.998 | 1.000 |
| pos_150 | 0.975 | 0.981 | 0.995 | 0.997 | 0.998 | 0.998 |
| pos_45 | 0.996 | 0.997 | 0.993 | 0.991 | 0.999 | 1.000 |
| neg_62 | 0.998 | 0.988 | 0.981 | 0.999 | 0.997 | 0.997 |
| pos_447 | 1.000 | 0.781 | 0.998 | 0.985 | 0.999 | 0.977 |
| pos_110 | 0.994 | 0.960 | 0.995 | 0.990 | 0.990 | 0.979 |
| pos_183 | 0.174 | 0.999 | 0.994 | 0.788 | 0.004 | 1.000 |
| pos_17 | 0.992 | 0.999 | 0.991 | 0.998 | 0.996 | 0.999 |
| pos_104 | 0.021 | 0.999 | 0.236 | 0.999 | 0.780 | 0.999 |
| neg_146 | 0.961 | 0.987 | 0.994 | 0.997 | 0.974 | 0.999 |
| pos_121 | 0.992 | 0.923 | 1.000 | 0.999 | 0.998 | 0.984 |
| neg_92 | 0.991 | 0.985 | 0.998 | 0.315 | 0.999 | 0.928 |
| pos_333 | 0.961 | 0.995 | 0.986 | 0.997 | 0.966 | 0.998 |
| pos_30 | 0.779 | 0.997 | 0.993 | 1.000 | 0.998 | 0.997 |
| neg_2 | 0.960 | 0.965 | 1.000 | 1.000 | 0.997 | 0.994 |
| neg_24 | 0.835 | 0.986 | 0.994 | 0.998 | 0.987 | 0.997 |
| pos_385 | 0.895 | 0.994 | 0.988 | 0.999 | 0.974 | 0.998 |
| neg_167 | 0.931 | 0.950 | 0.995 | 1.000 | 0.926 | 0.999 |
| pos_303 | 0.992 | 0.998 | 0.994 | 0.999 | 0.972 | 1.000 |
| pos_386 | 0.734 | 0.999 | 0.358 | 1.000 | 0.119 | 0.999 |
| pos_342 | 0.928 | 0.994 | 0.979 | 0.999 | 0.970 | 0.999 |
| pos_521 | 0.996 | 0.993 | 0.993 | 0.997 | 0.999 | 0.999 |
| neg_109 | 0.526 | 0.999 | 0.972 | 0.999 | 0.876 | 0.999 |

**Supplementary Table 2. The physiological and biochemical characteristics of the type strain Sy2-11.**

| **Characteristic** | **Sy2-11** | **Characteristic** | **Sy2-11** |
| --- | --- | --- | --- |
| **Biochemical test** |  | **Major fatty acids (>0.5%)** |  |
| Gelatin liquefaction | + | iso-C_13:0_ | 0.58 |
| Amylolysis | + | iso-C_14:0_ | 9.36 |
| Cellulose degradation | + | C_14:0_ | 1.47 |
| Nitrate reduction | + | iso-C_15:0_ | 10.59 |
| Urease test | + | anteiso-C_15:0_ | 10.61 |
| Tween 20 | + | iso-C_16:0_ H | 3.29 |
| Tween 40 | + | iso-C_16:0_ | 25.4 |
| Tween 80 | + | C_16:0_ | 10.54 |
| H_2_S production | − | anteiso-C_17:1_ ω9c | 2.13 |
| Spore ornamentation | Rugose | iso-C_17:0_ | 2.61 |
| Growth temperature | 10-40°C | anteiso-C_17:0_ | 3.4 |
| Growth pH | 5.0-9.0 | C_17:0_ *cyclo* | 4.95 |
| NaCl % (W/V) | 0-9% | C_17:0_ | 0.76 |
| **Major menaquinones (%)** |  | C_16:1_ 2-OH | 1.33 |
| MK9 (H_2_) | 21.406 | Summed Feature 8 (C_18:1_ ω7c) | 0.7 |
| MK9 (H_4_) | 13.689 | C_17:0_ 3-OH | 0.7 |
| MK9 (H_6_) | 27.646 | anteiso-C_19:0_ | 0.58 |
| MK9 (H_8_) | 37.258 | Summed Feature 3 (C_16:1_ ω7c / C_16:1_ ω6c) | 4.83 |
| **Carbon-source utilization** |  | Summed Feature 8 (C_18:1_ ω6c) | 1.16 |
| L-arabinose | + | Summed Feature 9 (C_17:1_ iso ω9c) | 3.01 |
| Glycerol | + | **Nitrogen-source utilization** |  |
| D-fructose | + | L-arginine | − |
| D-galactose | + | L-threonine | + |
| D-glucose | + | L-proline | + |
| D-mannose | + | Valine | + |
| D-sorbitol | + | L-histidine | + |
| D-trehalose | + | L-isoleucine | + |
| D-xylose | + | L-proline | − |
| Melibiose | + | L-asparagine | + |
| D-mannitol | + | Tyrosine | + |
| Inositol | + | DL-aspartic acid | + |
| Raffinose | + | L-leucine | − |
| L-rhamnose | + | L-glutamic acid | + |
| Soluble starch | + |  |  |
| Sucrose | + |  |  |
| Maltose | + |  |  |

**Supplementary Table 3. List of primers and probe sequences used in this study.**

| **Target** | **Primer or probe** | **Sequence (5’ - 3’)** |
| --- | --- | --- |
| **ITS** | ITS1 | TCCGTAGGTGAACCTGCGG/ |
|  | ITS4 | TCCTCCGCTTATTGATATGC |
| ***Fusarium oxysporum*** | W106F | GCAGTCGTACGTCATCGACC/ |
|  | W106R | CCATGGCAGATGGCGAGTCA |
| ***Foc*4** | *Foc*Sc-1 | CAGGGGATGTATGAGGAGGCTAGGCTA |
|  | *Foc*Sc-2 | GTGACAGCGTCGTCTAGTTCCTTGGAG |
| ***Foc* TR4** | *Foc* TR4-F | CACGTTTAAGGTGCCATGAGAG/ |
|  | *Foc* TR4-R | CGCACGCCAGGACTGCCTCGTGA |
| **16S rRNA** | 338F | ACTCCTACGGGAGGCAGCAG |
|  | 806R | GGACTACHVGGGTWTCTAAT |
| **16S rRNA** | 27F | AGAGTTTTGATCCTGGCTCAG |
|  | 1492R | GGTTACTTGTTAGGACTT |
| ***CheB*** | *CheB -UPF* | CGGTACCCGGGGATCCATTATTGATGTGATGGAG |
|  | *CheB-UPR* | TTGATTCATATCCAATTGATTCACTGTAAAGTCCTCCTCA |
|  | *CheB-DF* | TGAGGAGGACTTTACAGTGAATCAATTGGATATGAATCAA |
|  | *CheB-DR* | CGACGGCCAGTGCCAAGCTTCAAACAGCACATCCAG |
| ***CheC*** | *CheC* -UPF | CGGTACCCGGGGATCCAGCTTCCGCTGACACTT |
|  | CheC-UPR | CGACTGCGGCTTCCGCACGGCGTTGTCTTTTCCCCTTTAAG |
|  | *CheC*-DF | CTTAAAGGGGAAAAGACAACGCCGTGCGGAAGCCGCAGTCG |
|  | *CheC*-DR | GCCAGTGCCAAGCTTCATTCATCGTTGATACTA |
| ***CheD*** | *CheD*-UPF | CGGTACCCGGGGATCCGGAGAGCCTAATGCTTAA |
|  | *CheD*-UPR | CCCCCTACCTCATACCTTTAAAAGCCGCACCCAATGAGAC |
|  | *CheD*-DF | GTCTCATTGGGTGCGGCTTTTAAAGGTATGAGGTAGGGGG |
|  | *CheD*-DR | CGACGGCCAGTGCCAAGCTTGCAAGCTGAGCGATCAGCTC |
| ***CheF*** | *CheF*-UPF | CGGTACCCGGGGATCCGTCATGAACGGAACGGATAC |
|  | *CheF*-UPR | GGGCACCTCCTCGTGTCGTTTTGATTCACACCCTCTCTTT |
|  | *CheF*-DF | AAAGAGAGGGTGTGAATCAAAACGACACGAGGAGGTGCCC |
|  | *CheF*-DR | CGACGGCCAGTGCCAAGCTTCATTGCAGAAGCCGAGTGAC |
| **CheW** | *CheW* -UPF | CGGTACCCGGGGATCCACGGCATTGAATCGCCT |
|  | *CheW*-UPR | CTCATGTTGTCTTTTCCCCTTCGGGCACCTCCTCGTGTCG |
|  | *CheW*-DF | CGACACGAGGAGGTGCCCGAAGGGGAAAAGACAACATGAG |
|  | *CheW*-DR | CGACGGCCAGTGCCAAGCTTAAGCTTTGTCAAATCAGCCAA |

**Supplementary Table 4. Medium formulations employed in this study.**

| **Medium** | **Components** |
| --- | --- |
| Inorganic salt medium | (NH_4_)_2_SO_4_ 2.0 g L^-1^, MgSO_4_·7H_2_O 0.2 g L^-1^, CaCl_2_·2H_2_O 0.01 g L^-1^, FeSO_4_·7H_2_O 0.001 g L^-1^, Na_2_HPO_4_·12H_2_O 1.5 g L^-1^, KH_2_PO_4_ 1.5 g L^-1^ |

**Supplementary** **Methods**

**CLSM observations of banana root colonization by the GFP-Sy2-11 strain**

To test the colonization dynamics of strain Sy2-11 in the banana root, a high-resolution upright laser scanning microscope (LSM910, Zeiss, Germany) equipped with two emission-collecting channels was used to examine the banana root colonization patterns of a modified Sy2-11 strain overexpressing green fluorescent protein (GFP-Sy2-11), as described in ref.^61^. Briefly, the GFP gene was cloned into the pKNT-RP27-GFP vector using a one-step cloning kit (Vazyme Biotech, China), and transformed into the GFP-Sy2-11 mutant. Subsequently, GFP-tagged strain Sy2-11 (GFP-Sy2-11) was applied to the roots of each plant at a concentration of 1×10^6^ CFU/g soil. Seedlings with intact root systems were carefully extracted from the pot at 3, 7, and 14 dpi, and loosely adhering bulk soil was removed by gentle shaking. The roots were cleaned in sterile water and cut into 0.5 cm long sections for CLSM observation. The colonization dynamics of GFP-Sy2-11 in banana roots were monitored using a high-resolution upright laser scanning microscope (LSM910, Zeiss, Germany) equipped with two emission-collecting channels. Excitation/emission wavelengths were set from 488 nm for detection of the GFP signal.

**Colonization dynamics in the banana rhizosphere soil, rhizoplane, and endorhiza**

To determine the ability of strain Sy2-11 to colonize the banana rhizosphere soil, rhizoplane, and endorhiza, an ampicillin-resistant marker of strain Sy2-11 was used to quantify its population under axenic conditions, as described by ref.^61^. Briefly, strain Sy2-11 was applied to the roots of each plant at a concentration of 1×10^6^ CFU/g soil. Banana seedlings with intact root systems were carefully removed from the pot at 3, 7, and 14 dpi, and rhizosphere soil was collected. Roots were then transferred to 50 mL centrifuge tubes containing 25 mL sterile washing solution (0.9% NaCl and 0.02% Silwet L-77) and vortexed twice for 30 s. The rhizosphere suspension was centrifuged at 10,000 × g for 10 min, and the resulting pellet was resuspended in 4.5 mL washing solution. Serial dilutions (10^-1^ to 10^-3^) of the soil suspension and washing solution were prepared and plated on YE medium added with 350 mg/L ampicillin, 50 mg/L streptomycin, 50 mg/L K_2_Cr_2_O_7_, 50 mg/L cycloheximide, and 50 mg/L nystatin. Plates were incubated at 28 °C for 3-5 days. *Streptomyces* colonies were counted, and the concentration was expressed as CFU/g soil. To detect strain Sy2‑11 within root tissues, roots were surface sterilized by sequential immersion in 70% (v/v) ethanol for 5 min, 0.9% (v/v) NaClO for 10 min, 10% (w/v) NaHCO_3_ for 10 min, and sterile distilled water for 5 min. The roots were then washed in 5 ml washing solution, and 0.1 ml of the wash solution was plated on YE medium to confirm the absence of contaminants. Subsequently, the surface-sterilized roots were thoroughly ground in a sterile mortar, suspended in washing solution, and serially diluted. 100 mL of diluent (10^-1^ to 10^-3^) was spread on YE medium supplemented with 350 mg/L ampicillin, 50 mg/L streptomycin, 50 mg/L K_2_Cr_2_O_7_, 50 mg/L cycloheximide, and 50 mg/L nystatin. The *Streptomyces* concentration was determined as described above and expressed as CFU/g root.
