# Supplementary material for: Streptomyces sesquiterpenes elicit 10-HCA secretion and recruit disease-suppressive microbiota to enhance banana Fusarium wilt resistance
Source: Nat Commun. 2026 Jun 3;17:7138. doi: 10.1038/s41467-026-73928-x (PMC13396392; doi:10.1038/s41467-026-73928-x)
Supplement: Supplementary file 2 — Description of Additional Supplementary Files [file 41467_2026_73928_MOESM2_ESM.pdf]

## Description of Additional Supplementary Files

**File Name:** Supplementary Data 1

**Description:** Differentially expressed ASVs between CTL and PST identified using DESeq2 in Field 1.

**File Name:** Supplementary Data 2

**Description:** Summary of *Bacillus* isolates from the PST group and their inhibition rates against Foc TR4 on PDA plates.

**File Name:** Supplementary Data 3

**Description:** A total of 865 and 1029 DEGs were identified at 0.5h and 1h, respectively.

**File Name:** Supplementary Data 4

**Description:** Identification of 198 candidate metabolites through low-value filtering and differential metabolite analysis.
