# Supplementary material for: Streptomyces sesquiterpenes elicit 10-HCA secretion and recruit disease-suppressive microbiota to enhance banana Fusarium wilt resistance
Source: Nat Commun. 2026 Jun 3;17:7138. doi: 10.1038/s41467-026-73928-x (PMC13396392; doi:10.1038/s41467-026-73928-x)
Supplement: Supplementary file 5 — Transparent Peer Review file [file 41467_2026_73928_MOESM5_ESM.pdf]

nature portfolio

Peer Review File

Streptomyces sesquiterpenes elicit 10-HCA secretion and recruit disease-suppressive microbiota to enhance banana Fusarium wilt resistance

Corresponding Author: Professor Jianghui Xie

This file contains all reviewer reports in order by version, followed by all author rebuttals in order by version.

Version 1:

Reviewer comments:

Reviewer #1

(Remarks to the Author)
The authors have conducted extensive work to demonstrate that Streptomyces strain 2-11 suppresses banana Fusarium wilt through producing VOCs that induce the plant to synthesize 10-hydroxycapric acid (10-HCA), thereby recruiting beneficial Bacillus species. The study presents a logically coherent mechanism, supported by substantial data, and reveals an indirect biocontrol pathway mediated by microbial VOCs. However, the manuscript would be strengthened by addressing the lack of key data specifically characterizing the novel strain S. yongxingensis 2-11 itself, and by ensuring that all interpretations remain strictly within the bounds of the presented evidence. Overall this is a well-executed comprehensive study with a clear narrative and strong experimental design. However, there are areas where this work could be improved, expanded, or clarified. The weaknesses stemed largely from the study's depth-first approach, which leaves certain foundational questions unanswered.

Detailed comments.
1.This study does not explain how banana root detect Streptomyces VOCs or through which plant signaling pathways that are activated. The molecular steps between sesquiterpene detection and 10-HCA biosynthesis remain unknown limiting mechanistic understanding and predictive application.
2.Neglected Community Complexity: Bacillus is clearly important, while the functional roles of other enriched taxa are ignored. The synthetic community included only Bacillus, leaving open question that whether broader community interactions are necessary for full disease suppression in natural soils.
3.The suggested “hitchhiking” of Streptomyces on motile Bacillus is not sufficietnly supported. Only preliminary mycelial growth data is provied, without direct proof of enhanced spore dispersal or colonization in plant.
4.Lack of in situ validation for 10-HCA’s role: the strong in vitro chemotaxis data does not automatically confirm that 10-HCA is the primary driver of Bacillus enrichment in the rhizosphere. The cheW mutant was not tested in soil leaving ecological relevance uncertain.
5.The framing of the research objectives in Lines 76-90 is somewhat unconventional. Presenting the aims as questions that anticipate the key findings can be confusing for the reader. It would be beneficial to reframe these as clear and direct statements of the study's goals.
6.It is important to describe the origin and detailed characteristics of the novel strain S. yongxingensis sp. nov. 2-11 (e.g., source, isolation details, and key taxonomic traits) to establish its context and validity.
7.Nomenclaturely, referring to NCBI taxonomic notes, “S. yongxingensis” has been effectively published but not validly published under the rules of the International Code of Nomenclature of Prokaryotes (Bacteriological Code). Therefore, applying the name S. yongxingensis is not appropriate in literature as scientifically we don’t know what species you are talking about. Suggestion is to either validly publish this species, or if not possible, cite it as “Streptomyces sp. （JCMxxx）”
8.Similarly, banana Fusarium wilt (BFW) has been widely known as a polyphyletic assembledge, including many different species of Fusarium. Which species are you referring to in this experiment? Please refer to recent literature to identify your causal agent properly and provide its a correct name and full diagnosis characters.
(https://doi.org/10.1016/j.simyco.2018.06.003; https://doi.org/10.1111/ppa.13863;
https://doi.org/10.1016/j.simyco.2021.100116)
9.The conclusion stated in Lines 143-144 appears to lack direct support from the data presented in the manuscript. The authors should either provide the specific data that substantiate this claim or temper the conclusion to align more closely

with the actual findings.
10.Line 148: The term "core microbiota" requires a precise operational definition. It would strengthen the manuscript to state the specific parameters used for its identification, such as whether it is based on a presence/absence threshold (e.g., taxa present in >XX% of all samples) and if relative abundance was considered.
11.please clarify the results from the amplicon sequencing data regarding the relative abundance of Streptomyces following inoculation. Was a significant enrichment detected? If not, please discuss potential reasons.
12.Line 159: The conclusion appears overly absolute. Have the authors ruled out the possibility that the enrichment of Bacillus is simply a consequence of its intrinsic stress tolerance? Specifically, after Streptomyces inoculation, the bacterium produces antimicrobial metabolites that inhibit most members of the microbiota, whereas Bacillus owing to its robust resistance survives and becomes enriched, rather than being actively recruited by the plant. At least here, this conclusion is a bit beyond the scope.
13.Figure 3b: I recommend an alternative visualization, for example, a box plot that can display the median or average, along with jitter; the current format makes it impossible to discern which group is significantly higher.
14.Lines 176-179: The statement cannot be visually corroborated in Figure 3a; the depicted data do not provide an intuitive support for the described result.
15.For Bacillus, the 16S rRNA gene generally lacks sufficient resolution for reliable species-level discrimination; housekeeping loci such as gyrB and rpoB provide far greater phylogenetic granularity and should be employed to unambiguously assign isolates to species.
16.The mechanistic basis by which Streptomyces-derived volatile compounds elicit plant 10-HCA biosynthesis in the host plant remains unresolved. This point can be supplemented in the discussion or by adding additional plant transcriptome data.
17.Lines 590-591: Why was a Pearson correlation used to construct the co-occurrence network instead of Spearman? Microbiome count data are typically non-normally distributed and highly skewed, making rank-based (Spearman) methods more appropriate.
18.Line244 has an additional "."
19.The capitalization or italicization of p/P in the entire text should be consistent.
20.Line 303, Bacillus should be in italics.

Reviewer #2

(Remarks to the Author)
This study presents a compelling and novel mechanism of microbiome-mediated disease suppression. Authors investigated a novel tripartite interaction between Streptomyces yongxingensis Sy2-11, banana roots, and Bacillus spp. in suppressing banana Fusarium wilt (BFW). The authors demonstrate that sesquiterpenes (aristolene and ledene) released by Sy2-11 elicit banana roots to secrete 10-hydroxycapric acid (10-HCA), which selectively enriches Bacillus spp. with antifungal activity. This microbial recruitment leads to suppression of Fusarium oxysporum f. sp. cubense tropical race 4 (Foc TR4). The study integrates metabolomics, transcriptomics, chemotaxis assays, and synthetic community (SynCom) validation to support a mechanistic model of microbiome-mediated disease resistance.
The finding that Streptomyces-derived sesquiterpenes act as elicitors of plant root metabolites that recruit beneficial microbes is novel and represents a significant conceptual advance in plant-microbiome interactions. It shifts the paradigm from direct antagonism to microbe-plant-microbe signaling. However, the following criticisms still need to be addressed.

Major Comments:
1. It is relevant to compare the proposed mode of action relative to other known “cry for help” mechanisms (e.g., flavonoid-mediated recruitment). A more explicit comparison with existing systems is necessary to be discussed at least.
2. It is shown that 10-HCA is both necessary and sufficient to recruit Bacillus spp.. I wonder how aristolene and ledene activate 10-HCA biosynthesis in banana. Are these volatiles perceived by root cells directly, or via microbial intermediates? Is there a conserved plant signaling pathway?
3. With ecological relevance, authors should discuss whether the observed effects are likely to persist in natural soils with high microbial diversity, spatial heterogeneity, and environmental fluctuation. A field trial or soil microcosm experiment with native microbial background would strengthen the translational relevance.

Minor Comments
1. Statistical analysis needs to be further clarified. Several figures (e.g., Fig. 2c, 4d, 6b) lack exact p-values. Multiplicity corrections (e.g., FDR) are not applied to metabolomics or transcriptomics data. Replicate numbers for RNA-seq and metabolomics are not clearly stated.
2. Beside the sequencing data, metabolomics and transcriptomics raw data should be made publicly available prior to publication as well.

Reviewer #3

(Remarks to the Author)
The submitted manuscript described a mechanism of how Streptomyces yongxingensis sp. nov. 2-1 (Sy2-11) enhanced pathogen resistance to banana Fusarium wilt (BFW). This study used a variety of omic techniques demonstrating that Sy2-11 produced VOCs including sesquiterpenes aristolene and ledene. These VOCs led to altered banana root exudation with a markable increase in 10-hydroxycapric acid (10-HCA). 10-HCA exudation served as a chemoattractant and a nutrient source for Bacillus strain, subsequently shifting the community composition and antagonizing the Fusarium pathogen. In addition to the mechanistic insight, it also provides evidence of translational potential. Overall, the manuscript is well-written

and I enjoy reading this manuscript thoroughly. I have a few suggestions to improve this manuscript.

Major points

1. Where is Sy2-11 is isolated from, soil, banana rhizosphere or BFW disease suppressive soil? Have you included the strain information in any public database? Please make sure this information is available.
2. Ln 132, the authors stated that ‘these results demonstrate that the microbiota mediated by strain Sy2-11 establishes robust disease resistance’. However, no microbiota data was shown at this point so I suggest removing or revising this statement.
3. Based on Fig 2b, no enrichment of Streptomyces strains in the PST treatment was apparent. Is that an indication of poor survival/colonization with banana? Please generate a reporter strain of Sy2-11. If not, single strain inoculation followed by gentle washing step to reveal Sy2-11 colonization pattern will be very helpful.
4. Regarding the ability of Sy2-11 to produce sesquiterpenes with subsequent plant-dependent biocontrol activity, is that a more general property of Streptomyces or specific to Sy2-11. I noticed that the inoculum is relatively high i.e. × 10^7 CFU/g soil. One can argue that such biocontrol property is unlikely to occur in nature due to low concentration of a single Streptomyces sp.. Please clarify.
5. Related to point 4, there are numerous reports of Bacillus-mediated disease protection. I wonder whether this strategy involving Sy2-11 is more robust or long-lasting than direct inoculation with a disease suppressive Bacillus consortium (as shown in Fig. 7). To strengthen this point, a comparison using different concentrations of Sy2-11 (and other Streptomyces strains as requested in point 4 above) with subsequent quantitation of disease severity will be helpful. This is essential as the proposed model stated some sort of plant root colonization by Streptomyces is needed, but it is not demonstrated.
6. Is 10-HCA a nutrient source of Sy2-11. If so, it may provide biological insight for the induction pattern.
7. How about other enriched metabolites e.g. Docosapentaenoic acid and 8,11 eicosadiynoic acid. Why are they not selected for further analyses? Some discussion will be helpful.
8. Is the SynCom experiment shown in Fig. 7 performed under non-sterile condition? To investigate the effect of Sy2-11 on disease resistance through a recruitment of Bacillus spp., I would suggest including Sy2-11 treated germ-free plants in the presence of a Bacillus-free communities. If that is not feasible, please use sterile soil followed by Foc infection. I would expect that the protective effect by Sy2-11 will be lost or weaken due to the absence of antagonistic Bacillus strains. This is recommended as another S. yongxingensis strain e.g. JCM 34965, was previously shown to produce the antifungal compound niphimycin C. Although the authors have a no-plant control i.e. SST throughout the manuscript, it does not rule out a possibility that an antifungal compound will be produced only in the presence of plants, thus contributing to the observed phenotype.

Minor points

9. Some of the figure legends are lacking in details. I suggest reworking to make them more self-explanatory.
• Fig 2g is unclear to me and many labels are repetitive. I am not sure how to interpret a LDA value of 4.78, is that relevant?
• I am not sure what kind of information is conveyed in Fig 2f with no indication of what IncMSE represents.
• Same for Fig. 3e, information is not sufficiently provided for me to understand the dots. If the authors are using the same color scheme as in Fig 3a, please be specific. Consider highlighting some non-Bacillus strains that were shown to be highly enriched in the PST treatment.
• For Fig. 6e and f. What are the dots, what the colors represent, how can we understand the scale (0-100). It is not clear to me how this plot gives rise to a conclusion that microbial compositions between the strain Sy2-11and 10-HCA treatment groups are similar. I do not see particularly strong enrichment of dots along the axes. Please represent the data in another format that is more intuitive.
• Fig 6f appears redundant.
10. The materials and methods could benefit from some clarification.
• No information provided regarding the culturing condition of Sy2-11.
• What kind of hydroponic system and what media are used in the cultivation.
11. Have you checked for plant root colonization after three days of spore inoculation [Ln621] for the collection of metabolites?
12. Have you checked for potential contamination between the left and right chamber of the split root system? How do you make sure the metabolites are coming from plants or from microbes too?

Version 2:

Reviewer comments:

Reviewer #1

(Remarks to the Author)
The manuscript has been improved significantly. However, several issues remain to be addressed, including inconsistencies between the data and their descriptions, overstatements that extend beyond the evidence presented, and a number of minor points.

-1. Inconsistencies between data and description: Lines 167-177. The description of the co-occurrence network results is not fully supported by the data presented in the figures.
First, regarding the experimental design for assessing the effect of Sy2-11 inoculation: To evaluate its impact on the

rhizosphere microbiome, a comparison between the PST and CTL groups is necessary. To evaluate its impact on the bulk soil microbiome, a comparison between the SST and IS groups would be required.
Second, the authors state: "the average number of edges per node was similar between the strain Sy2-11 treated and SST groups, both of which were lower than the CTL group (Fig. 3d). "However, based on Fig. 3d, it is difficult to visually ascertain whether the PST (presumably Sy2-11 treated) group is more similar to the SST group or to the CTL group. Statistical significance markers should be clearly presented to support this claim.
Subsequently, the authors conclude that "pathogen pressure disrupts microbiome structure, reducing network complexity and stability." The provided data do not sufficiently support this conclusion. Contrary to the authors' claim that Sy2-11 inoculation "enhances network complexity and stability," the data in Fig. 3d suggest that inoculation may have reduced network complexity (as indicated by the lower average edges per node) compared to the healthy CTL group. Furthermore, claims of enhanced stability require support from dedicated stability metrics, such as Robustness, Vulnerability, Fragility, Compositional stability, the ratio of negative to positive correlations, or cohesion, rather than simple node and edge counts. A more rigorous assessment of stability would ideally involve time-series data.
Finally, the authors claim: "The co-occurrence networks further indicated higher proportions of positive edges and modularity in the strain Sy2-11 treated group networks compared to the CTL and SST groups (Fig. 3a)." This cannot be deduced from the network visualization in Fig. 3a alone. Conversely, the data presented in Fig. 3c suggest that the SST group may have a higher proportion of positive correlations. Quantitative results for the proportion of positive edges and modularity index should be provided in a separate panel to substantiate this claim.

-2. Overstatements / Claims extending beyond the presented evidence.
Lines 27-32 (Abstract): Certain claims in the abstract may extend beyond the scope strongly supported by the data. The description of the mechanism "whereby Streptomyces, acting as a novel and beneficial elicitor, releases sesquiterpene signals to trigger 10-HCA secretion in banana plants" is presented as a new finding. However, stating that this constitutes a "novel strategy" might be an overstatement, as the general strategy of using microbial inoculation to indirectly manipulate the rhizosphere microbiome for disease suppression has been reported previously. It is recommended to temper this claim, for instance, by describing it as a "potential" or "promising" novel strategy.
A similar adjustment is suggested for the statement in Lines 468-469.
Line 561: The phrase "microbiome-oriented biological breeding" is puzzling in this context, as the present study does not involve work related to plant genotype or breeding programs. Its inclusion here is unclear and potentially misleading.

-3. Minor points
To enhance readability, it is suggested to add a brief introductory description of strain Sy2-11 at the beginning of the Results section (around Line 94).
Regarding formatting:
In Figure 2, the "P" in P-values should be italicized.
All microbial genus names in the text and figures should be formatted in italics.
Data specification check:
Line 411: Please verify and specify whether the values reported are log10FC or log2FC.
References require the following corrections:
Line 931: "N. Phytol." should be corrected to "New Phytol."
Line 966: "N. Phytol." should be corrected to "New Phytol."
Line 970: "Arabidopsis thaliana" should be italicized.

Reviewer #2

(Remarks to the Author)
The authors conducted series of experiments and have nicely addressed all comments in the revised manuscript. The discussion section is overly lengthy. Discussions can be more focused and concise. Please revise it accordingly.

Reviewer #3

(Remarks to the Author)
The authors have addressed most of my concerns/suggestions in the revision. However, the manuscript still required additional clarification, specifically on the colonization competence of Sy2-11 strain.
If I understood correctly, Sy2-11 was isolated from marine soft coral collected from 15-20m depth of the ocean. It is surprising that this marine strain can colonize and survive on banana root. The authors have performed multiple experiments but they are not entirely conclusive.
1. The use of 16S-based sequencing failed to distinguish Sy2-11 from strains sharing the same partial 16S sequences as reflected in the detection of the same ASV in non-inoculated control.
2. What are those thread-like structures represent in Supp Fig 2. Are you suggesting that those are live bacteria? What is the time point for observation (i.e. how many days after inoculation)? If only early time point is taken, it may not reflect true colonization, see below.
3. Fig R3 needed to be strengthened. First, information related to the input inoculum, method of inoculation, time of incubation and the washing condition were missing. Second, the time of co-incubation should be long enough to match the experimental design to have a protective effect. Third, if the experiment was performed using high input and short time of incubation, one cannot exclude the possibility that the strains were thriving on residual nutrients without persistent colonization. For instance, if you repeat the experiment using dead plant materials, maybe you can see the same pattern of colonization.

4. As stated before, direct plant root colonization was not demonstrated. Provided that Streptomyces strains survived under harsh condition (for example upon changes from marine to soil), it is possible that Sy2-11 were germinating as a survival strategy and produced metabolites (including antibiotics) that shaped microbial composition. SST is not a perfect control as explained before. Without a 10-HCA biosynthesis mutant, one cannot rule out this possibility. Regardless, I do not think it will affect the major conclusions but the authors should address these clearly in the result section based on evidence rather than speculation. The model has to be revised accordingly.
5. Based on reference 61, Sy2-11 was growing on M1, M2, M3, and M4 agar media. Please specify the composition of the media and clarify whether this medium carried high amount of salt or specific nutrients to mimic the marine environment.
6. New data should be integrated as part of the manuscript. Specifically, Fig R4 related to the dosage dependent effect of Sy2-11 on banana Fusarium wilt disease severity should be included as a main figure. Note that the use of strain 5-6 and H7 with comparable input (10^6 cfu/g) also reduced disease incidence, suggesting that inoculation of Streptomyces strains with high concentration have some general protective effect, supporting the suspicion that 10-HCA independent mechanism contributed significantly to protection. Please discuss accordingly. Same for the data related to the variations in protective effect in the presence or absence of indigenous microbiota.

Version 3:

Reviewer comments:

Reviewer #1

(Remarks to the Author)
My comments have been considered and satisfactorily addressed. I have no other comments.

Reviewer #3

(Remarks to the Author)
The authors have addressed all my concerns especially on the colonization competence of Sy2-11 on banana. The model is strengthened and well-supported. I still find some textual edits needed but I believe it can be addressed during the proofing process without another round of revision.

In 118: trial instead of trail
In 150: if these streptomyces strains were not sequenced, I cannot tell the whether these strains are similar to Sy2-11. It is better to remove the word 'homologous' if the authors are not sure.
In 411-412. If the authors insist that these data cannot be included in the manuscript. Please remove the statement 'Our study showed that the direct antagonistic effect of strain Sy2-11 against Foc TR4 was completely lost in sterilized soil'.

Open Access This Peer Review File is licensed under a Creative Commons Attribution 4.0 International License, which permits use, sharing, adaptation, distribution and reproduction in any medium or format, as long as you give appropriate credit to the original author(s) and the source, provide a link to the Creative Commons license, and indicate if changes were

made.
In cases where reviewers are anonymous, credit should be given to 'Anonymous Referee' and the source.
The images or other third party material in this Peer Review File are included in the article’s Creative Commons license, unless indicated otherwise in a credit line to the material. If material is not included in the article’s Creative Commons license and your intended use is not permitted by statutory regulation or exceeds the permitted use, you will need to obtain permission directly from the copyright holder.
To view a copy of this license, visit https://creativecommons.org/licenses/by/4.0/

Feb-26-2026

Dear Reviewers:

We highly appreciate your favorable assessment of our work and are happy to submit a revised version of our manuscript. According to Reviewers' suggestions, we provide a point-by-point explanation of the changes and our response to your comments. The amendments are highlighted in red in the manuscript.

Response to Reviewer #1:

Point 1: This study does not explain how banana root detect Streptomyces VOCs or through which plant signaling pathways that are activated. The molecular steps between sesquiterpene detection and 10-HCA biosynthesis remain unknown limiting mechanistic understanding and predictive application.

Response to comment: We greatly appreciate your careful review of our manuscript and the valuable comments provided. To reveal the downstream signal pathways activated by banana roots sensing *Streptomyces* VOCs and the molecular mechanism of 10-HCA biosynthesis, firstly, we employed a split-root system to treat banana roots with ledene, and transcriptome analysis was performed at 0, 3, 6, and 12 h post-treatment. Principal component analysis (PCA) revealed both excellent reproducibility among biological replicates and significant transcriptional differences between the treatment and control groups (**Supplementary Fig. 18a**). Differentially expressed genes (DEGs) were identified via DESeq2. A total of 1001, 1626, and 1317 up-regulated genes were detected at 3 h, 6 h, and 12 h, respectively, among which 573 genes were commonly up-regulated across the three time points (**Supplementary Fig. 18b**).

Secondly, VOCs, sesquiterpenes are one of the key mediators during plant-plant communication, within-plant self-signaling, and plant-microbe interactions. However, their recognition receptors and downstream signal transduction pathways remain poorly understood. Previous studies have demonstrated that PhKAI2ia, a karrikin-insensitive receptor in Petunia, can stereospecifically perceive the (-)-germacrene D signal, trigger the KAI2-mediated signal cascade, and thereby

modulate plant fitness (Stirling et al., 2024). Similarly, we identified five homologous genes of the KAI2 gene that perceives (-)-germacrene D in banana, whereas only the g24040 gene was significantly enriched at 12 h ($|log_2FC| > 1$ and $P < 0.05$, **Supplementary Fig. 18c**). Another study has reported that TOPLESS-like proteins (TPLs), which are involved in VOC sensing in tobacco, act as transcriptional co-suppressors with *β*-caryophyllene-binding activity (Nagashima et al., 2019). Similarly, we identified 11 homologous genes encoding TOPLESS-like proteins in banana, among which g12130, g09280, and g04820 were significantly downregulated at both T6h and T12h ($|log_2FC| < -1$ and $P < 0.05$, Fig. **Supplementary Fig. 18c**). This result indicates that the sesquiterpene ledene modulates the expression of these receptor genes, implying that this VOC is likely perceived by the banana root system. In summary, several important questions remain to be addressed. First, the recognition pathways and receptors for the sesquiterpenes aristolene and ledene in banana remain unclear. Second, the downstream signal transduction pathways in bananas activated by these two sesquiterpenes are still unknown. Therefore, elucidating their recognition mechanisms and pathways constitutes an intriguing topic for future research. However, the main focus and objective of the present study were to clarify the microecological mechanisms underlying sesquiterpene-mediated disease resistance in banana induced by *Streptomyces*. Studies on plant receptors and signal transduction pathways will form the subject of a separate project, which we aim to pursue in future research.

Thirdly, the biosynthetic pathway of 10-HCA remains elusive, yet numerous researchers have proposed that its biosynthesis may originate from fatty acid metabolism. The Up-regulated DEGs were annotated using Gene ontology (GO) and Kyoto Encyclopedia of Genes and Genomes (KEGG), respectively. Four enzymes associated with fatty acid metabolism were identified, including fatty acid acyl-CoA binding enzyme (g12360), fatty acid desaturase (g03200/g33950) and fatty acid hydroxylase (g20040), all of which were significantly up-regulated in the treatment groups at 3 h, 6 h and 12 h (**Supplementary Fig. 18d**). In addition, seven cytochrome

P450 genes potentially related to fatty acid metabolism were identified. Among them, g05480 showed the highest fold change, followed by g14290; both genes were significantly up-regulated at 3 h, 6 h, and 12 h. Elucidating the biosynthetic pathway of 10-HCA in plants demands a more stringent experimental design, in conjunction with the functional characterization of key genes via molecular biological strategies. The present study aimed to investigate how 10-HCA functions in the regulation of rhizosphere microecology and the establishment of disease resistance. The biosynthetic pathway of 10-HCA was not the focus of this work, but it will be one of the key priority directions for our follow-up research.

Moreover, we have supplemented and further discussed this content in the Discussion section to improve the structure and completeness of the manuscript. For details, please refer to the revised manuscript, lines 470-495 (Discussion) and lines 764-781 (Methods).

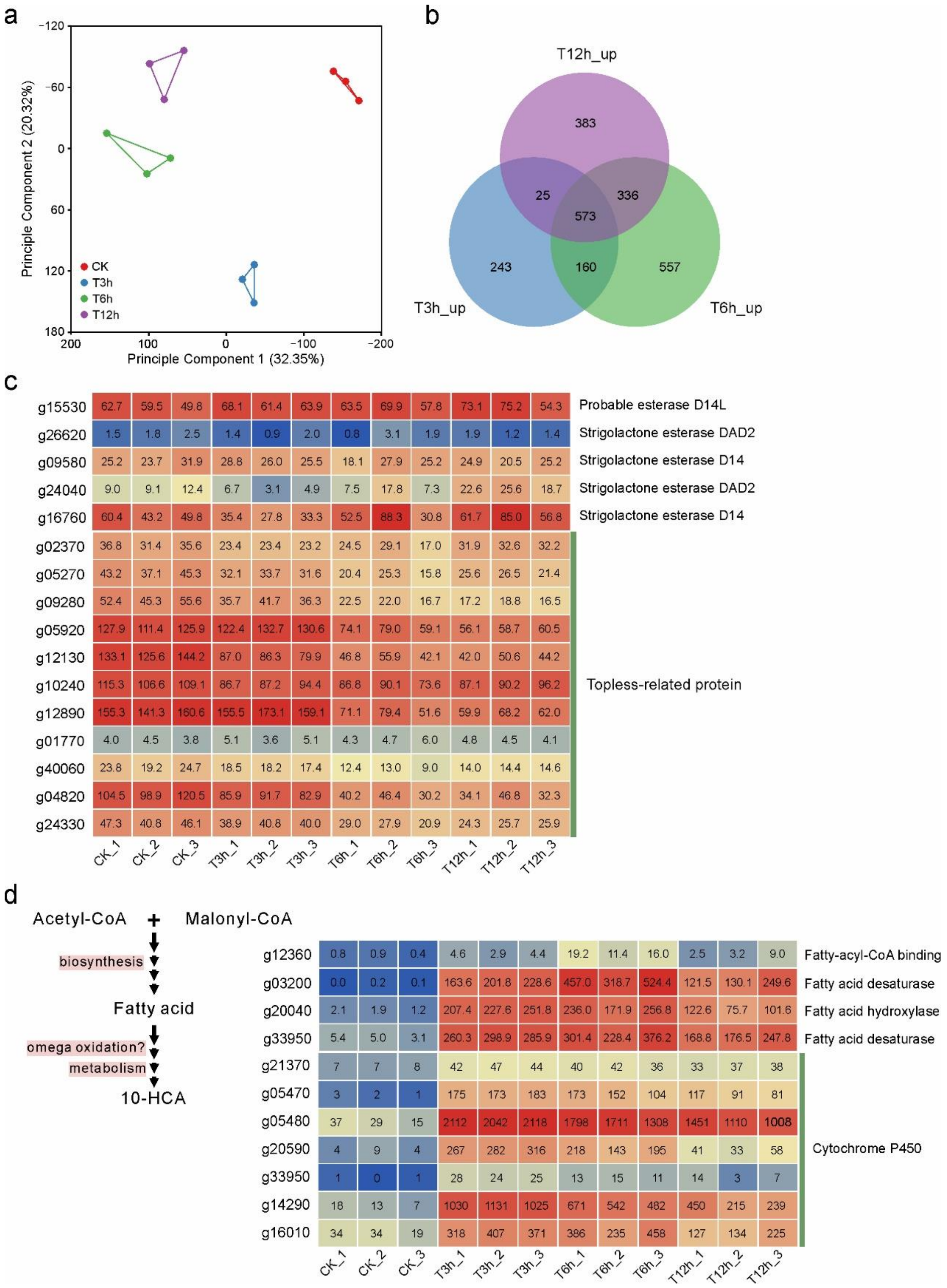

Supplementary Fig. 18. Identification of genes involved in 10-HCA biosynthesis and regulatory genes for sesquiterpene ledene perception in banana roots. (a) Principal component analysis (PCA) of transcriptome sequencing demonstrated excellent reproducibility among biological replicates and distinct transcriptional differences between the treatment and control groups. (b) A total of 1001, 1626, and 1317 upregulated genes were identified at 3 h, 6 h, and 12 h, respectively, with 573 genes commonly upregulated at all three time points. (c) 5 homologous genes of the KAI2 gene responsible for (-)-germacrene D perception and 11 homologous genes

encoding TOPLESS-like proteins responsible for *β*-caryophyllene perception were identified in banana. (d) 11 enzymes associated with fatty acid metabolism were identified.

Point 2: Neglected Community Complexity: Bacillus is clearly important, while the functional roles of other enriched taxa are ignored. The synthetic community included only Bacillus, leaving open question that whether broader community interactions are necessary for full disease suppression in natural soils.

Response to comment: Thank you for your valuable comments. We agree that the microbial community is indeed very complex. How other enriched taxa function in the testing system is yet unknown. In this study, we observed that *Bacillus* exhibited the highest relative abundance in the PST group. We therefore identified *Bacillus* as the key microbe conferring disease resistance. This study aimed to verify this conclusion, rather than investigate interactions between other high-abundance microbial groups. Synergistic interactions may exist between other microbial taxa and *Bacillus*, and this aspect is surely interesting, which will be the focus of further independent and in-depth research in our follow-up studies.

Point 3: The suggested “hitchhiking” of Streptomyces on motile *Bacillus* is not sufficietnly supported. Only preliminary mycelial growth data is provided, without direct proof of enhanced spore dispersal or colonization in the plant.

Response to comment: Thank you for your insightful comments. To further clarify our findings, we have provided more evidences including *in situ* validation of rhizosphere interactions between strain Sy2-11 and *Bacillus* isolates, which demonstrates that *Bacillus* isolates promote the colonization of strain Sy2-11 on banana roots. These data will support our conclusion on “hitchhiking” of *Streptomyces* on motile *Bacillus*. We have substantially revised the text relevant to this question, refer to lines 536-541.

Point 4: Lack of in situ validation for 10-HCA’s role: the strong in vitro chemotaxis data do not automatically confirm that 10-HCA is the primary driver of *Bacillus* enrichment in the rhizosphere. The cheW mutant was not tested in soil leaving ecological relevance uncertain.

Response to comment: Thank you for raising this important point regarding an in-situ validation of 10-HCA and the *cheW* mutant. To verify the effect of 10-HCA on BFW incidence and the rhizosphere microbial community, we performed an *in-situ*

validation of the role of 10-HCA through a mesoscale experiment in a greenhouse. The experimental design included three treatments: (1) CTL: control treatment consisted of plants treated with sterilized water; (2) PST: plants inoculated with strain Sy2-11; (3) HCA: plants inoculated with 10-HCA. Results showed that after challenging the seedlings with Foc TR4, seedlings in both the strain Sy2-11 and 10-HCA treatments remained healthy, in contrast to the control (Fig. 6a-b; Supplementary Table 11-12). Comparative analysis revealed that *Bacillus* was the most abundant genus with the most significant fold change in the 10-HCA group (Fig. 6f), suggesting that the mechanism of suppressing wilt disease is likely consistent with the way strain Sy2-11 recruits *Bacillus* spp. to suppress Foc TR4, resulting in robust growth of banana plants. Results are in lines 268-297 of the revised version.

To further verify the role of *CheW* in the *Bacillus* spp. chemotaxis and disease-suppressive, we performed an *in-situu* validation of strain Bac155 and the *ΔcheW* mutant strain via a compartmentalized rhizobox system. Analysis of soil samples revealed that the T*ΔcheW* group still exhibited a significantly higher *Foc* TR4 abundance (2.87×10^4 CFU/g soil) compared to the CTL group. In contrast, the *Foc* TR4 abundance in the rhizosphere of TBac155 group was significantly reduced (3.82×10^3 CFU/g soil, $P < 0.05$, Duncan′s test). The results further showed that the disease indices of the CTL group and T*ΔcheW* group were 70.83% and 65.28%, respectively, while that of the TBac155 group was only 29.17%. This indicates that strain Bac155 migrated towards and colonized the rhizosphere where 10-HCA was applied, thereby effectively reducing the incidence of *Foc* TR4. Experimental methods are presented in lines 872-887, and the results are presented in lines 351-361 of the revised version.

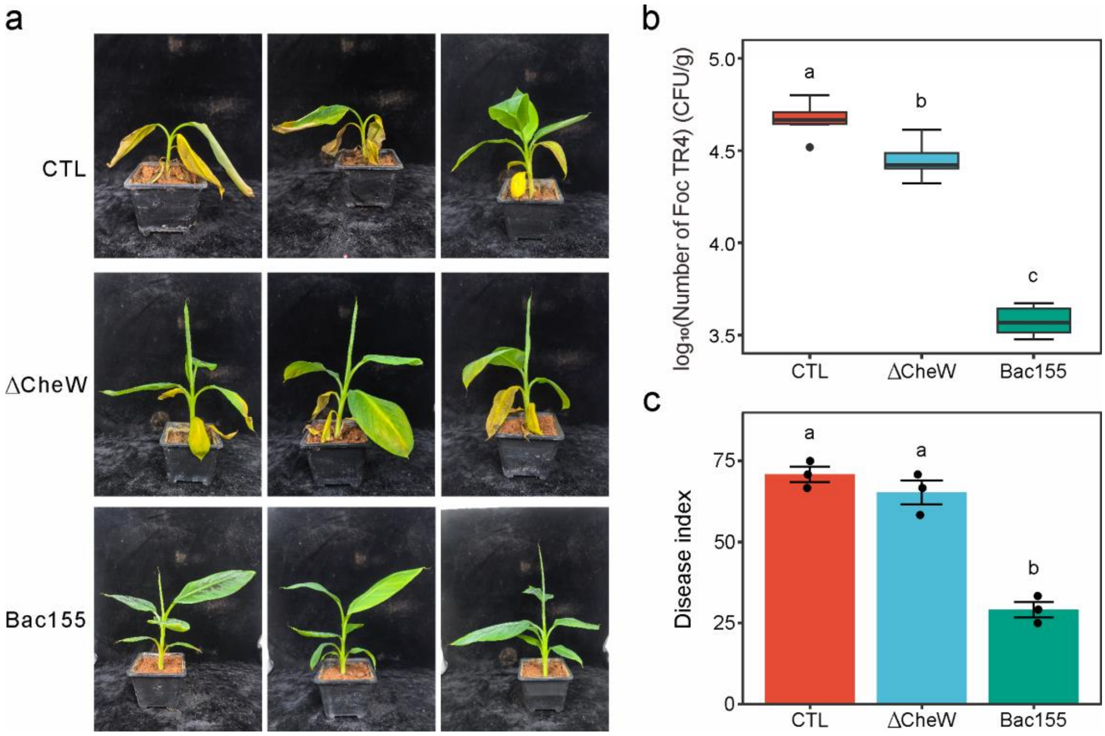

Supplementary Fig. 17. In-situ validation of the suppression ability of strain Bac155 and its *ΔcheW* mutant against *Foc* TR4 and BFW. (**a**) phenotypic comparison of banana seedlings following treatment with strain Bac155 and the *ΔcheW* mutant strain. Seedlings in the Bac155 group showed healthy growth, while those in the CTL and *ΔcheW* group exhibited obvious symptoms of Fusarium wilt disease. Images were taken at 35 dpi. (**b**) Comparison of *Foc* TR4 content, showing a significant reduction in pathogen load in the Bac155 group. (**c**) Disease index comparison among the CTL, *ΔcheW*, and Bac155 groups. CTL: control treatment consisted of plants treated with sterilized water; *ΔcheW*: plants inoculated with the *ΔcheW* mutant strain; Bac155: plants inoculated with strain Bac155. Statistical significance was assessed using LSD multiple comparison tests, with different letters indicating significant differences ($P < 0.05$).

Point 5: The framing of the research objectives in Lines 76-90 is somewhat unconventional. Presenting the aims as questions that anticipate the key findings can be confusing for the reader. It would be beneficial to reframe these as clear and direct statements of the study's goals.

Response to comment: Thank you for this insightful suggestion. We fully agree with your opinion. We have revised this section to clarify our research objectives more explicitly. For details, please refer to the revised manuscript at lines 77-79.

Point 6: It is important to describe the origin and detailed characteristics of the novel strain *S. yongxingensis* sp. nov. 2-11 (e.g., source, isolation details, and key taxonomic traits) to establish its context and validity.

Response to comment: Thank you for your careful review and constructive comments on our manuscript. We have supplemented detailed information on the

novel strain *S. yongxingensis* sp. nov. 2-11 in the revised manuscript, including its source, isolation details, cultural, physiological, and biochemical characteristics, as well as genomic data. Please refer to lines 564-577 in the revised manuscript.

Point 7: Nomenclaturely, referring to NCBI taxonomic notes, "S. yongxingensis" has been effectively published but not validly published under the rules of the International Code of Nomenclature of Prokaryotes (Bacteriological Code). Therefore, applying the name S. yongxingensis is not appropriate in literature as scientifically we don't know what species you are talking about. Suggestion is to either validly publish this species, or if not possible, cite it as "Streptomyces sp. （JCMxxx）".

Response to comment: Thank you for your valuable suggestion. In accordance with the International Code of Nomenclature of Prokaryotes (Bacteriological Code), we have deposited the strain in the Guangdong Microbial Culture Collection Center (GDMCC, China) and the Japan Collection of Microorganisms (JCM, Japan). We have obtained the certificate of deposit with accession numbers GDMCC 4.213 and JCM 34965. Upon publication of this article, we will promptly submit the published manuscript to the depositories for formal registration.

Point 8: Similarly, banana Fusarium wilt (BFW) has been widely known as a polyphyletic assembledge, including many different species of Fusarium. Which species are you referring to in this experiment? Please refer to recent literature to identify your causal agent properly and provide its a correct name and full diagnosis characters. (https://doi.org/10.1016/j.simyco.2018.06.003; https://doi.org/10.1111/ppa.13863; https://doi.org/10.1016/j.simyco.2021.100116).

Response to comment: Thank you for your insightful and professional comments. Following the references you recommended, we have added information on the cultural characteristics, morphological features, and genomic phylogenetic tree of the strain in the revised manuscript. The *Foc* TR4 strain was identified and classified as belonging to the same taxon as the *Foc* TR4 strain No.58 reported in Yun et al. (2019, doi: 10.1094/MPMI-03-19-0063-A; Supplementary Fig. 21). Please refer to lines 611-618 in the revised manuscript.

Point 9: The conclusion stated in Lines 143-144 appears to lack direct support from the data presented in the manuscript. The authors should either provide the specific data that substantiate this claim or temper the conclusion to align more closely with

the actual findings.

Response to comment: We thank the reviewer for pointing this out. We have revised the relevant sentences in the revised manuscript to minimize confusion for readers. Refer to lines 140-142 in the revised version, such as: Overall, inoculation of strain Sy2-11 to banana significantly affected the composition of the rhizosphere bacterial community.

Point 10: Line 148: The term "core microbiota" requires a precise operational definition. It would strengthen the manuscript to state the specific parameters used for its identification, such as whether it is based on a presence/absence threshold (e.g., taxa present in >XX% of all samples) and if relative abundance was considered.

Response to comment: Thank you for your careful review and constructive comments on our manuscript. In this study, core microbiota were defined as those detected in more than 70% of samples, with relative abundance ranked in the top 1%. Across all groups, *Bacillus* ranked second in relative abundance, while in the PST group, it ranked the first. These parameters suggest that *Bacillus* is likely one of the core microorganisms associated with disease resistance. The relevant parameters have been added to the manuscript as suggested by the reviewer. please refer to lines 147-148 in the revised manuscript

Point 11: please clarify the results from the amplicon sequencing data regarding the relative abundance of Streptomyces following inoculation. Was a significant enrichment detected? If not, please discuss potential reasons.

Response to comment: We greatly appreciate the reviewer’s valuable comments. Following your suggestion, we reanalyzed the original data. *Streptomyces* was highly enriched in the PST group, and the mean relative abundance of the genus *Streptomyces* in the PST group was significantly higher than that in the other groups. Furthermore, in the co-occurrence network presented in Figure 3a, *Streptomyces* from the PST group occupied a central position in the network structure. The relative abundance of its ASVs was also significantly higher than in the CTL and SST groups, and all edges linked to its node represented positive regulatory interactions.

Point 12: Line 159: The conclusion appears overly absolute. Have the authors ruled

out the possibility that the enrichment of Bacillus is simply a consequence of its intrinsic stress tolerance? Specifically, after Streptomyces inoculation, the bacterium produces antimicrobial metabolites that inhibit most members of the microbiota, whereas Bacillus owing to its robust resistance survives and becomes enriched, rather than being actively recruited by the plant. At least here, this conclusion is a bit beyond the scope.

Response to comment: Thank you for your thorough review of our manuscript and for your valuable feedback. First, based on the results presented in Figure 2c, Figure 3e, and Supplementary Fig. 10b, *Bacillus* was indeed significantly enriched in the PST group, with a far greater number of corresponding ASVs than in the CTL and SST groups.

Second, 95.6% of the *Bacillus* isolates we obtained exhibited antifungal activity, indicating that these functional *Bacillus* strains were selectively recruited by the banana rhizosphere, rather than merely surviving and becoming enriched simply due to their strong drug resistance.

Third, the results shown in Figure 4 and Figure 6 demonstrated that *Bacillus* growth could be rapidly promoted by HCA as a carbon source both on plates and in soil, suggesting that the enrichment of *Bacillus* is more likely an active process rather than passive selection.

We acknowledge that the description of this conclusion was inaccurate in the original manuscript, and we have revised the conclusion in the revised version, refer to line 158-161.

Point 13: Figure 3b: I recommend an alternative visualization, for example, a box plot that can display the median or average, along with jitter; the current format makes it impossible to discern which group is significantly higher.

Response to comment: Thank you for your careful review of our article and for providing valuable feedback. For Fig. 3b, we have revised it to a combination of a violin plot and a box plot. The box plot allows for comparison of means and quartiles, while the violin plot enables comparison of data density distribution. This provides a more intuitive and direct visual presentation of the data.

Point 14: Lines 176-179: The statement cannot be visually corroborated in Figure 3a; the depicted data do not provide an intuitive support for the described result.

Response to comment: We thank the reviewer for pointing out the errors in our manuscript and offer our sincere apologies for this oversight. We have removed this section from the revised manuscript.

Point 15: For Bacillus, the 16S rRNA gene generally lacks sufficient resolution for reliable species-level discrimination; housekeeping loci such as gyrB and rpoB provide far greater phylogenetic granularity and should be employed to unambiguously assign isolates to species.

Response to comment: We greatly appreciate your careful review of our manuscript and the valuable comments provided. We sequenced the 16S rRNA genes of 260 *Bacillus* isolates and matched them against the core ASVs. The isolate sequences were assigned to the ASVs based on 100% sequence similarity. A total of 103 isolates were aligned to four ASVs (ASV0024, ASV0031, ASV0350, and ASV7217). Therefore, we performed whole-genome sequencing on the 103 aligned isolates to achieve accurate identification at the species level. Taxonomic identification of the strains comprising the synthetic microbial community (SynCom), based on average nucleotide identity (ANI) and DNA–DNA hybridization (DDH) values, is provided in the Supplementary Materials. We have also revised this section in the manuscript.

Point 16: The mechanistic basis by which Streptomyces-derived volatile compounds elicit plant 10-HCA biosynthesis in the host plant remains unresolved. This point can be supplemented in the discussion or by adding additional plant transcriptome data.

Response to comment: Thanks a lot and we think this is an excellent suggestion. Similar to comment 1 above, in accordance with your suggestions, we employed a split-root system to treat banana roots with ledene, and collected young root samples at 0, 3, 6, and 12 h post-treatment for subsequent transcriptome sequencing and analysis. Principal component analysis (PCA) revealed both excellent reproducibility among biological replicates and significant transcriptional differences between the treatment and control groups (**Supplementary Fig. 18a**). Differentially expressed genes (DEGs) were identified via DESeq2. A total of 1001, 1626, and 1317 up-regulated genes were detected at 3 h, 6 h, and 12 h, respectively, among which 573

genes were commonly up-regulated across the three time points (**Supplementary Fig. 18b**).

The biosynthetic pathway of 10-HCA remains elusive, yet numerous researchers have proposed that its biosynthesis may originate from fatty acid metabolism. The Up-regulated DEGs were annotated using Gene ontology (GO) and Kyoto Encyclopedia of Genes and Genomes (KEGG), respectively. Four enzymes associated with fatty acid metabolism were identified, including fatty acid acyl-CoA binding enzyme (g12360), fatty acid desaturase (g03200/g33950) and fatty acid hydroxylase (g20040), all of which were significantly up-regulated in the treatment groups at 3 h, 6 h and 12 h (**Supplementary Fig. 18d**). In addition, seven cytochrome P450 genes potentially related to fatty acid metabolism were identified. Among them, g05480 showed the highest fold change, followed by g14290; both genes were significantly up-regulated at 3 h, 6 h, and 12 h. Elucidating the biosynthetic pathway of 10-HCA in plants demands a more stringent experimental design, in conjunction with the functional characterization of key genes via molecular biological strategies. The present study aimed to investigate how 10-HCA functions in the regulation of rhizosphere microecology and the establishment of disease resistance. The biosynthetic pathway of 10-HCA was not the focus of this work, but it will be one of the key priority directions for our follow-up research.

In addition, following the reviewer's suggestions, we have supplemented and expanded the relevant content in the Discussion section to further improve the structure and completeness of the manuscript. For details, please refer to the revised manuscript, lines 470-495 (Discussion) and lines 764-781 (Methods).

Point 17: Lines 590-591: Why was a Pearson correlation used to construct the co-occurrence network instead of Spearman? Microbiome count data are typically non-normally distributed and highly skewed, making rank-based (Spearman) methods more appropriate.

Response to comment: We greatly appreciate the reviewer's professional and rigorous comment on the correlation method for co-occurrence network construction. Following the reviewer's suggestions, we re-analyzed and constructed the

co-occurrence network based on the Spearman correlation coefficient, and calculated the network-related properties using the igraph package. Accordingly, we have comprehensively revised Figure 3 and updated it in the revised manuscript, and also made corresponding modifications in the results section (Lines 180-183). The results show that the network constructed using the Spearman correlation coefficient exhibits only minor changes compared with the previous version, while the overall results remain largely consistent. We sincerely appreciate the reviewer's highly professional comments once again.

Point 18: Line244 has an additional ".".
Response to comment: Thank you for pointing out the error. The authors are very sorry for this mistake and have corrected it.

Point 19: The capitalization or italicization of p/P in the entire text should be consistent.
Response to comment: Thank you for your meticulous review of our formatting. We are very sorry for this mistake, and corrections have been made accordingly in the revised manuscript.

Point 20: Line 303, Bacillus should be in italics.
Response to comment: Thank you for your meticulous review of our formatting. We have corrected it in our revised version. Refer to line 301 in the revised version.

Response to Reviewer #2

Major Comments:

Point 1: It is relevant to compare the proposed mode of action relative to other known "cry for help" mechanisms (e.g., flavonoid-mediated recruitment). A more explicit comparison with existing systems is necessary to be discussed at least.

Response to comment: We thank the reviewer for the valuable suggestions. We have added a comparison of the "cry for help" mechanisms in the discussion section to highlight the differences between our mechanism and those previously reported. For details, please refer to lines 432-444.

Point 2: It is shown that 10-HCA is both necessary and sufficient to recruit Bacillus spp.. I wonder how aristolene and ledene activate 10-HCA biosynthesis in banana. Are these volatiles perceived by root cells directly, or via microbial intermediates? Is there a conserved plant signaling pathway?.

Response to comment: We appreciate the reviewers' valuable comments. The reviewers have incisively raised a pertinent and excellent scientific question. First, in this study, we used virus-free tissue-cultured banana plantlets, and all operations and cultivation were performed under axenic conditions. We therefore conclude that these sesquiterpene VOCs are directly perceived by root cells.

Second, we employed a split-root system to treat banana roots with ledene, and collected young root samples at 0, 3, 6, and 12 h post-treatment for subsequent transcriptome sequencing and analysis. Principal component analysis (PCA) revealed both excellent reproducibility among biological replicates and significant transcriptional differences between the treatment and control groups (**Supplementary Fig. 18a**). Differentially expressed genes (DEGs) were identified via DESeq2. A total of 1001, 1626, and 1317 up-regulated genes were detected at 3 h, 6 h, and 12 h, respectively, among which 573 genes were commonly up-regulated across the three time points (**Supplementary Fig. 18b**).

However, the biosynthetic pathway of 10-HCA remains elusive, yet numerous researchers have proposed that its biosynthesis may originate from fatty acid metabolism. The Up-regulated DEGs were annotated using Gene ontology (GO) and Kyoto Encyclopedia of Genes and Genomes (KEGG), respectively. Four enzymes

associated with fatty acid metabolism were identified, including fatty acid acyl-CoA binding enzyme (g12360), fatty acid desaturase (g03200/g33950) and fatty acid hydroxylase (g20040), all of which were significantly up-regulated in the treatment groups at 3 h, 6 h and 12 h (**Supplementary Fig. 18d**). In addition, seven cytochrome P450 genes potentially related to fatty acid metabolism were identified. Among them, g05480 showed the highest fold change, followed by g14290; both genes were significantly up-regulated at 3 h, 6 h, and 12 h. Elucidating the biosynthetic pathway of 10-HCA in plants demands a more stringent experimental design, in conjunction with the functional characterization of key genes via molecular biological strategies. The present study aimed to investigate how 10-HCA functions in the regulation of rhizosphere microecology and the establishment of disease resistance. The biosynthetic pathway of 10-HCA was not the focus of this work, but it will be one of the key priority directions for our follow-up research.

Third, VOCs, sesquiterpenes are one of the key mediators during plant-plant communication, within-plant self-signaling, and plant-microbe interactions. However, their recognition receptors and downstream signal transduction pathways remain poorly understood. Previous studies have demonstrated that PhKAI2ia, a karrikin-insensitive receptor in Petunia, can stereospecifically perceive the (-)-germacrene D signal, trigger the KAI2-mediated signal cascade, and thereby modulate plant fitness (Stirling et al., 2024). Similarly, we identified five homologous genes of the KAI2 gene that perceives (-)-germacrene D in banana, whereas only the g24040 gene was significantly enriched at 12 h ($|\log_2 FC| > 1$ and $P < 0.05$, **Supplementary Fig. 18c**). Another study has reported that TOPLESS-like proteins (TPLs), which are involved in VOC sensing in tobacco, act as transcriptional co-suppressors with *β*-caryophyllene-binding activity (Nagashima et al., 2019). Similarly, we identified 11 homologous genes encoding TOPLESS-like proteins in banana, among which g12130, g09280, and g04820 were significantly downregulated at both T6h and T12h ($|\log_2 FC| < -1$ and $P < 0.05$, Fig. **Supplementary Fig. 18c**). This result indicates that the sesquiterpene ledene modulates the expression of these receptor genes, implying that this VOC is likely perceived by the banana root system.

In summary, several important questions remain to be addressed. First, the recognition pathways and receptors for the sesquiterpenes aristolene and ledene in banana remain unclear. Second, the downstream signal transduction pathways in bananas activated by these two sesquiterpenes are still unknown. Therefore, elucidating their recognition mechanisms and pathways constitutes an intriguing topic for future research. However, the main focus and objective of the present study were to clarify the microecological mechanisms underlying sesquiterpene-mediated disease resistance in banana induced by *Streptomyces*. Studies on plant receptors and signal transduction pathways will form the subject of a separate project, which we aim to pursue in future research.

In addition, following the reviewer's suggestions, we have supplemented and expanded the relevant content in the Discussion section to further improve the structure and completeness of the manuscript. For details, please refer to the revised manuscript, lines 470-495 (Discussion) and lines 764-781 (Methods).

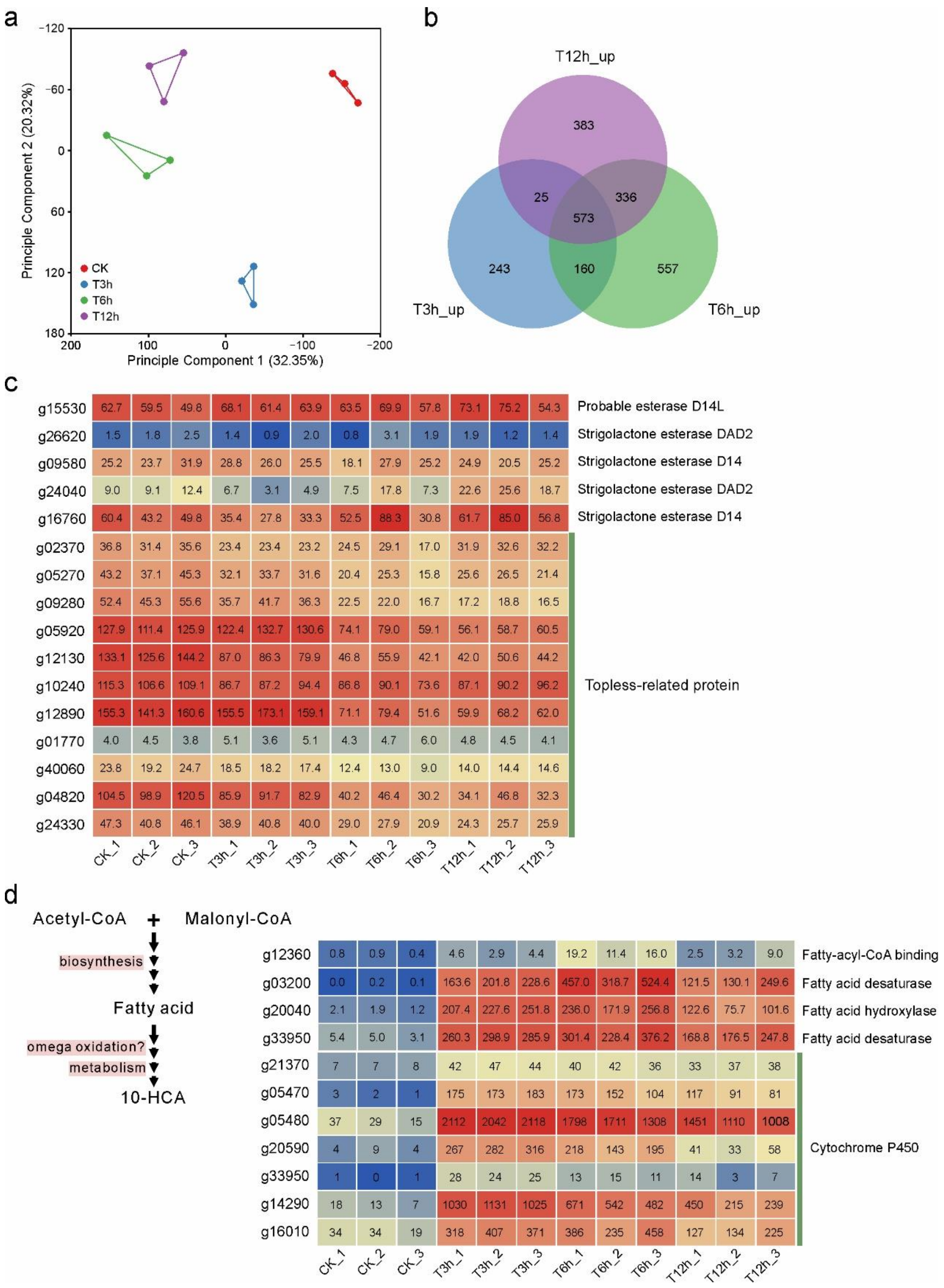

Supplementary Fig. 18. Identification of genes involved in 10-HCA biosynthesis and regulatory genes for sesquiterpene ledene perception in banana roots. (a) Principal component analysis (PCA) of transcriptome sequencing demonstrated excellent reproducibility among biological replicates and distinct transcriptional differences between the treatment and control groups. (b) A total of 1001, 1626, and 1317 upregulated genes were identified at 3 h, 6 h, and 12 h, respectively, with 573 genes commonly upregulated at all three time points. (c) 5 homologous genes of the KAI2 gene responsible for (-)-germacrene D perception and 11 homologous genes encoding TOPLESS-like proteins responsible for β-caryophyllene perception were identified in banana. (d) 11 enzymes associated with fatty acid metabolism were identified.

Point 3: With ecological relevance, authors should discuss whether the observed effects are likely to persist in natural soils with high microbial diversity, spatial heterogeneity, and environmental fluctuation. A field trial or soil microcosm experiment with native microbial background would strengthen the translational relevance.

Response to comment: We appreciate the reviewer's suggestions. In accordance with your advice, we collected soils from three different habitats, including non-agricultural soil, conventional field soil, and paddy soil. These soils were treated using the method described in this study, and the microbiome was analyzed while the disease control efficacy was evaluated. The results showed that after treatment with strain Sy2-11, *Bacillus* was significantly enriched in all three soil treatment groups. The disease indices of non-agricultural soil, conventional field soil, and paddy soil were 22.22%, 18.06%, and 19.44%, respectively, representing reductions of 51.39%, 52.78%, and 50.00% compared with the control group. In future work, we will also formulate strain Sy2-11 into a bacterial agent for field application and conduct further research.

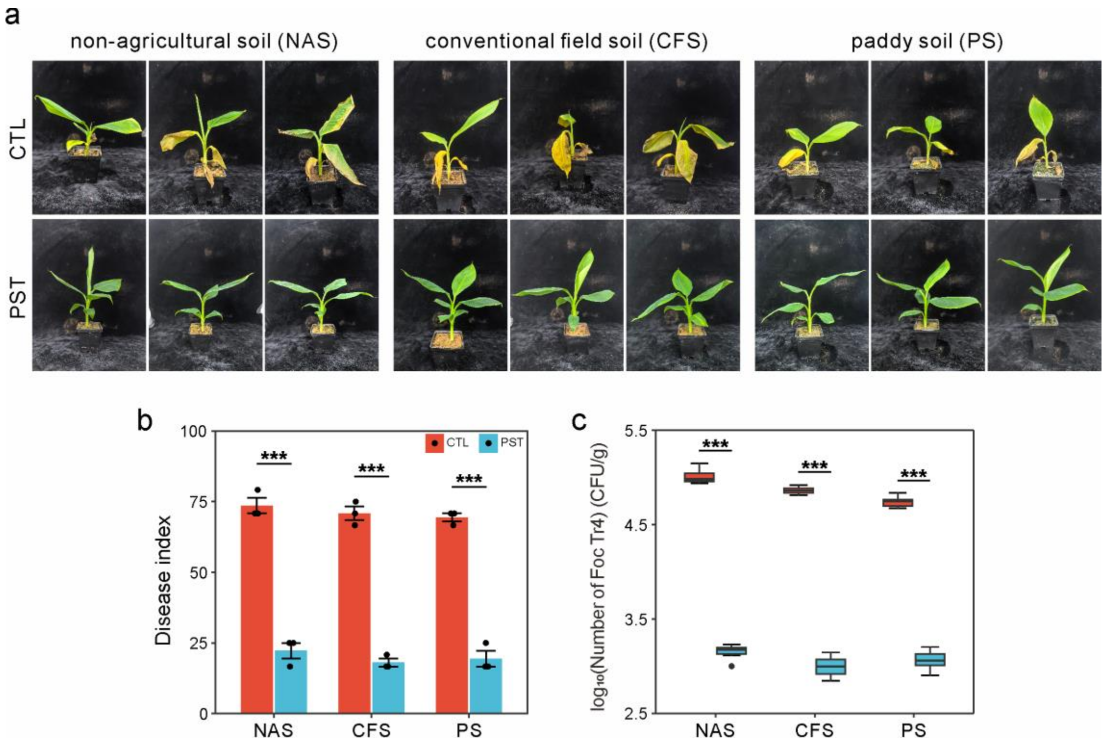

Fig. R1. Strain Sy2-11 reduces the incidence of banana *Fusarium* wilt (BFW) in non-agricultural soil, conventional soil, and paddy soil, respectively. (a) phenotypic comparison of banana seedlings following treatment with strain Sy2-11. Images were taken at 35 dpi. (d) Comparison of *Foc* TR4 content, showing a significant reduction in pathogen load in the

PST group. (b) Disease index comparison among the CTL and PST groups. CTL: control treatment consisted of plants treated with sterilized water; PST: plants inoculated with strain Sy2-11. Statistical significance was assessed using LSD multiple comparison tests, with different letters indicating significant differences ($P < 0.05$).

Minor Comments:

Point 1: Statistical analysis needs to be further clarified. Several figures (e.g., Fig. 2c, 4d, 6b) lack exact p-values. Multiplicity corrections (e.g., FDR) are not applied to metabolomics or transcriptomics data. Replicate numbers for RNA-seq and metabolomics are not clearly stated.

Response to comment: We appreciate your valuable suggestions for improving the quality of the manuscript. For Fig. 2c, a new supplementary table (Supplementary Table 1) has been added in this study, which documents the detailed identification results generated by DESeq2, including the exact *P*-values. For Fig. 4d, another supplementary table (Supplementary Table 5) has been newly included, which records the chemotaxis index It of the three *Bacillus* strains for the top 40 metabolites, as well as whether the It values are significantly less than 0.4 or significantly greater than 0.6. In addition, asterisks (*) have been added to the legend of Fig. 4d to indicate statistical significance at $P < 0.05$: red asterisks denote values significantly greater than 0.6, while blue asterisks represent values significantly lower than 0.4. Furthermore, statistical tests have been performed for Figs. 4g, 5d, 6c, and 8d, with the exact *P* values or significance markers for multiple testing annotated on the respective figures. Meanwhile, the legends of multiple figures have been supplemented with more detailed descriptive information.

Point 2: Beside the sequencing data, metabolomics and transcriptomics raw data should be made publicly available prior to publication as well.

Response to comment: We appreciate your valuable suggestions for improving the quality of the manuscript. We have uploaded the transcriptome data of *Bacillus* and bananas to the project with the accession number PRJNA1203960. The metabolomics data have been included in the Source Data. We have included detailed descriptions in the Data Availability section of the manuscript.

Response to Reviewer #3

Point 1: Where is Sy2-11 is isolated from, soil, banana rhizosphere or BFW disease suppressive soil? Have you included the strain information in any public database? Please make sure this information is available.

Response to comment: Thanks for the Reviewer's valuable suggestion. Following your advice, we have supplemented the revised manuscript with detailed information on the novel strain *S. yongxingensis* sp. nov. 2-11, including its source, isolation details, cultural, physiological, and biochemical characteristics, as well as genomic data.

In accordance with the International Code of Nomenclature of Prokaryotes (Bacteriological Code), the strain has been deposited and maintained at the Guangdong Microbial Culture Collection Center (GDMCC, China) and the Japan Collection of Microorganisms (JCM, Japan). We have obtained the certificate of deposit with accession numbers GDMCC 4.213 and JCM 34965. Upon publication of this article, we will promptly submit the published manuscript to the depositories for formal registration. Please refer to lines 564-577 in the revised manuscript for details.

Point 2: Ln 132, the authors stated that 'these results demonstrate that the microbiota mediated by strain Sy2-11 establishes robust disease resistance'. However, no microbiota data was shown at this point so I suggest removing or revising this statement.

Response to comment: We thank the reviewer for pointing this out. We have removed this sentence in the revised manuscript.

Point 3: Based on Fig 2b, no enrichment of Streptomyces strains in the PST treatment was apparent. Is that an indication of poor survival/colonization with banana? Please generate a reporter strain of Sy2-11. If not, single strain inoculation followed by gentle washing step to reveal Sy2-11 colonization pattern will be very helpful.

Response to comment: Thank you for your valuable comments. *Streptomyces* ranked 65th in abundance in the microbiome. We aligned the sequence of strain Sy2-11 with ASVs. The ASVs matching strain Sy2-11 (identity > 99%) ranked 123rd and 256th in abundance in Field 1 and Field 2, respectively. These data indicate that the relative abundance of Sy2-11 in the rhizosphere soil microbial community is low. Although

the abundance of ASVs corresponding to strain Sy2-11 appeared higher in Field 1, no significant differences were found among groups by LSD multiple comparison test; similarly, no significant differences were observed in Field 2 (Fig. R2).

Strain Sy2-11 was isolated from a marine soft coral and thus should not be present in banana rhizosphere soil. However, reads of these ASVs were still detected in the uninoculated IS and CTL groups. This suggests that strain Sy2-11 cannot be distinguished from native *Streptomyces* in banana orchard soil based solely on 400-bp reads from amplicon sequencing. Strain Sy2-11 may not maintain high abundance in rhizosphere soil. Nevertheless, our banana root colonization assay confirmed that this strain can effectively colonize the surface of banana roots (Supplementary Fig. 2). Following the reviewer's suggestions, we performed gentle washing after single-strain inoculation and carried out plate detection, which also verified the presence of strain Sy2-11 (Fig. R3).

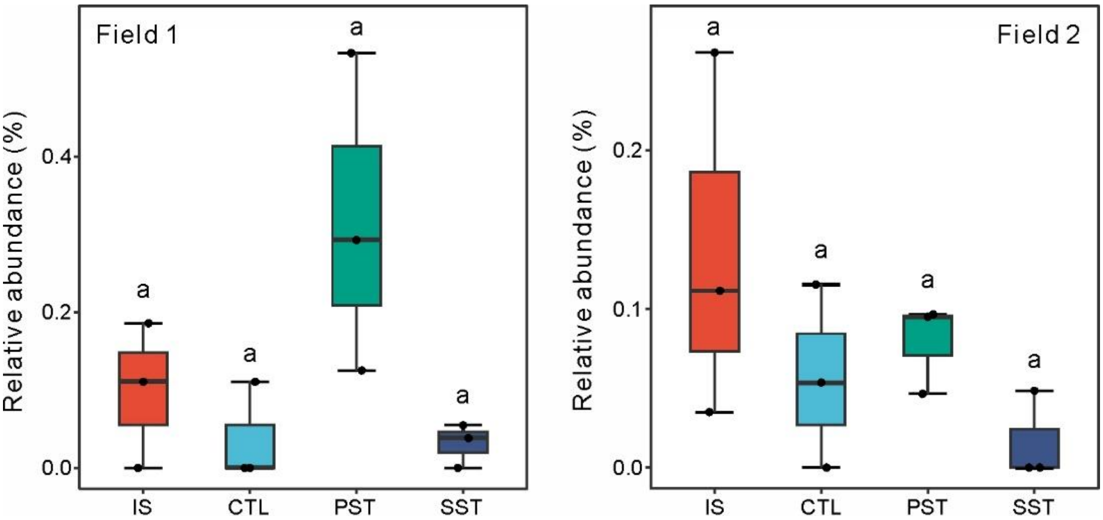

Fig. R2. The abundance of ASVs that matched strain Sy2-11.

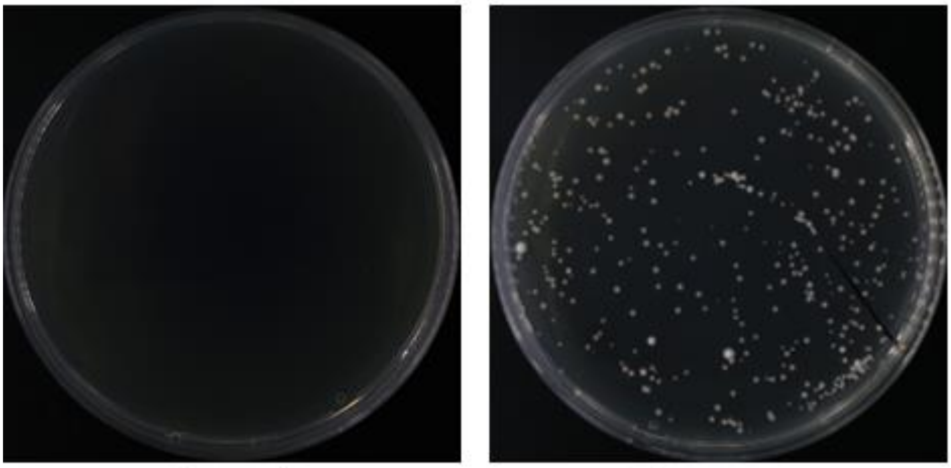

Fig. R3. Colonization of Strain Sy2-11 on banana roots. Control: without strain Sy2-11; Treatment: inoculated strain Sy2-11.

Point 4: Regarding the ability of Sy2-11 to produce sesquiterpenes with subsequent plant-dependent biocontrol activity, is that a more general property of Streptomyces or specific to Sy2-11. I noticed that the inoculum is relatively high i.e. × 10^7 CFU/g soil. One can argue that such biocontrol property is unlikely to occur in nature due to low concentration of a single Streptomyces sp.. Please clarify..

Response to comment: We appreciate the reviewer's comment. Strain Sy2-11 is a novel *Streptomyces* species isolated in our study. Under the same culture conditions used in our previous experiments, sesquiterpenes (aristolene and ledene) were not detected in the VOCs produced by other *Streptomyces* strains. The ability of strain Sy2-11 to produce sesquiterpenes and thereby induce 10-HCA secretion in banana plants is a unique characteristic of this strain.

First of all, we thank the reviewer for pointing this out. we sincerely apologize for the writing errors in the manuscript and have corrected them accordingly. Regarding the inoculation dosage, we determined the inoculation level of $\times 10^6$ CFU/g soil based on several previously published relevant studies (Wei et al., 2020, doi: 10.3389/fmicb.2020.01712; Li et al., 2021, https://doi.org/10.1016/j.biocontrol.2020.104524; Yun et al., 2022, https://doi.org/10.1094/PHYTO-11-21-0464-R; Zhang et al., 2021, doi: 10.3389/fmicb.2021.763038). The inoculation concentration of *Streptomyces* employed in this study was identical to that of the pathogen *Foc* TR4.

Next, to clarify this issue more explicitly, we performed a new experiment in

accordance with the reviewer's comments to re-evaluate the inoculation dosage of strain Sy2-11 and its colonization status. We established six experimental groups: (1) control treatment with sterilized water, and inoculated with strain Sy2-11 at a concentration of (2) 1×10^7 CFU/g soil, (3) 1×10^6 CFU/g soil, (4) 1×10^5 CFU/g soil, (5) 1×10^4 CFU/g soil, (6) 1×10^3 CFU/g soil. The results showed that when inoculated at 10^7 , 10^6, and 10^5 CFU/g soil, the colonization counts of strain Sy2-11 in soil were 3.30×10^3, 2.77×10^3, and 1.83×10^3 CFU/g soil, respectively. In contrast, the colonization count of strain Sy2-11 in soil was only 367 CFU/g soil when inoculated at 10^3 CFU/g soil.

Point 5: Related to point 4, there are numerous reports of Bacillus-mediated disease protection. I wonder whether this strategy involving Sy2-11 is more robust or long-lasting than direct inoculation with a disease suppressive Bacillus consortium (as shown in Fig. 7). To strengthen this point, a comparison using different concentrations of Sy2-11 (and other Streptomyces strains as requested in point 4 above) with subsequent quantitation of disease severity will be helpful. This is essential as the proposed model stated some sort of plant root colonization by Streptomyces is needed, but it is not demonstrated.

Response to comment: Thanks for the Reviewer's comments. First, our experimental results indicate that the strategy involving strain Sy2-11 is more robust and persistent than direct inoculation with the disease-suppressive *Bacillus* consortium. Second, following the reviewer's suggestion, we employed a range of concentrations of strain Sy2-11 (10^3 ~10^7 CFU g^{-1} soil) and selected two additional *Streptomyces* strains (5-6 and H7) from our laboratory culture collection that were isolated from the same source as Sy2-11. We performed a new experiment to evaluate the effect of the inoculum dosage of strain Sy2-11 on the disease index of banana *Fusarium* wilt. We established eight mental groups: (1) control treatment with sterilized water (CTL), (2) inoculated with *Streptomyces* sp. 5-6 at a concentration of 1×10^6 CFU/g soil (Str. 5-6), (3) inoculated with *Streptomyces* sp. H7 at a concentration of 1×10^6 CFU/g soil (Str. H7), and inoculated with strain Sy2-11 at a concentration of (4) 1×10^7 CFU/g soil, (5) 1×10^6 CFU/g soil, (6) 1×10^5 CFU/g soil, (7) 1×10^4 CFU/g soil, (8) 1×10^3 CFU/g soil. All treatments were inoculated with *Foc* TR4.

The results showed that the disease index of the control group was 79.17%, which

was highly significantly higher than that of all other treatments. Inoculation with strain Sy2-11 at concentrations of 1×10^7, 1×10^6, and 1×10^5 CFU/g soil yielded disease indices of 18.06%, 19.44%, and 20.83%, respectively, with no significant differences among these three treatments. The disease index following inoculation at 1×10^4 CFU/g soil was 27.78%, which was significantly higher than the three high-concentration treatments mentioned above. Furthermore, the disease index for inoculation at 1×10^3 CFU/g soil was 37.50%, which was significantly higher than that at 1×10^4 CFU/g soil. However, the two other *Streptomyces* strains showed the highest disease indices: 45.83% for strain 5-6 and 48.61% for strain H7 (Fig. R4).

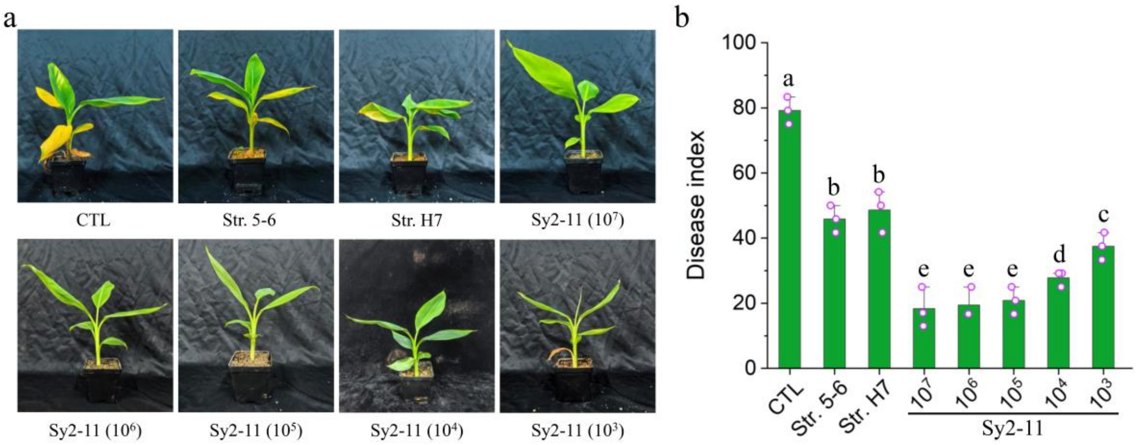

Fig. R4. The inhibitory effect of inoculating different concentrations of strain Sy2-11 against banana *Fusarium* wilt. (a) phenotypic comparison of banana seedlings following treatment with different concentrations of strain Sy2-11. (b) Disease index comparison among inoculating different concentrations of strain Sy2-11. CTL: control treatment consisted of plants treated with sterilized water; Str. 5-6: plants inoculated with strain 5-6. Str. H7: plants inoculated with strain H7. Statistical significance was assessed using LSD multiple comparison tests, with different letters indicating significant differences ($P < 0.05$).

Point 6: Is 10-HCA a nutrient source of Sy2-11. If so, it may provide biological insight for the induction pattern.

Response to comment: We thank the reviewer for the valuable suggestions. Following your advice, we investigated the effect of 10-HCA on the growth of strain Sy2-11. Excitingly, in vitro experiments showed that 10 μM and 100 μM 10-HCA significantly promoted the growth of strain Sy2-11 compared with common carbon sources (D-fructose, D-glucose, D-mannitol, and sucrose) (Supplementary Fig. 13). Specifically, relative to sucrose (the optimal carbon source), 10 μM 10-HCA

increased the cell number of strain Sy2-11 by 2.22-fold, while 100 μM 10-HCA enhanced it by 5.75-fold. In contrast, 1000 μM 10-HCA inhibited the growth of strain Sy2-11, with its cell abundance only reaching 0.3×10^3 (Fig. 4f, g). We have supplemented the relevant content in the Methods, Results, and Discussion sections of the revised manuscript, respectively. Please refer to lines 242-250 in the revised manuscript for details.

Point 7: How about other enriched metabolites e.g. Docosapentaenoic acid and 8,11 eicosadiynoic acid. Why are they not selected for further analyses? Some discussion will be helpful.

Response to comment: Thanks for the reviewer's suggestions, To explore the mechanisms underlying the recruitment of *Bacillus* spp. in the rhizosphere, we used microfluidic chemotaxis assays to evaluate the chemotactic responses of *Bacillus* isolates to the top 40 upregulated metabolites. These top 40 metabolites include Rose oxide, Dipyridamole, Calicogorgin A, 2-Ethyl-5-imino-1-cyclopenten-1-ol, Perindopril, 7Z, 10Z, 13Z, 16Z, 19Z-docosapentaenoic acid, o-Cresol, Taurine, 2-heptenedial, 10-hydroxy capric acid, N6-Methyl-L-lysine, Arginyl-Threonine, Adipic acid, 1-Stearoyl-2-hydroxy-sn-glycero-3-phosphoethanolamine, Raffinose, 8,11-eicosadiynoic acid, 3-ketosphingosine, etc. (Details are shown in Fig. 4d). Among which docosapentaenoic acid and 8,11-eicosadiynoic acid were ranked 6th and 16th, respectively. The results showed that the *Bacillus* strain exhibited no chemotaxis towards these two compounds.

Point 8: Is the SynCom experiment shown in Fig. 7 performed under non-sterile condition? To investigate the effect of Sy2-11 on disease resistance through a recruitment of Bacillus spp., I would suggest including Sy2-11 treated germ-free plants in the presence of a Bacillus-free communities. If that is not feasible, please use sterile soil followed by Foc infection. I would expect that the protective effect by Sy2-11 will be lost or weaken due to the absence of antagonistic Bacillus strains. This is recommended as another S. yongxingensis strain e.g. JCM 34965, was previously shown to produce the antifungal compound niphimycin C. Although the authors have a no-plant control i.e. SST throughout the manuscript, it does not rule out a possibility

that an antifungal compound will be produced only in the presence of plants, thus contributing to the observed phenotype.

Response to comment: We thank the reviewer for the suggestions. The SynCom experiment shown in Fig. 7 was conducted under axenic conditions, as described in Lines 804-805 of the methods section.

To investigate potential mechanisms by which the strain Sy2-11 controls the banana Fusarium wilt, specifically, whether it acts through metabolite production or by driving the assembly of the rhizosphere microbiome. We established a generic assay by inoculating strain Sy2-11 in sterile soil and natural soil to assess its impact on BFW incidence. In natural soil, after 35 days of inoculation, we observed that the disease incidence of banana seedlings inoculated with strain Sy2-11 was only 13.33%, representing a 60% reduction compared to the control. However, when the soil was sterilized, the disease incidence of banana seedlings inoculated with strain Sy2-11 was 66.67%, which was 13% lower than that of the control group. Results showed that strain Sy2-11 significantly reduced BFW incidence in the presence of rhizosphere microbiome.

We also offer our sincere apologies for the ambiguous wording in our manuscript. To this end, we have made corresponding revisions in the manuscript to ensure that the expressions are clearer and more comprehensible. For details, please refer to lines 102-104 in the revised manuscript.

Point 9: Some of the figure legends are lacking in details. I suggest reworking to make them more self-explanatory.

(1) Fig 2g is unclear to me and many labels are repetitive. I am not sure how to interpret a LDA value of 4.78, is that relevant?

Response to comment: We appreciate your valuable suggestions. We have updated the taxonomic information of the labels, where the prefixes c__, o__, f__, g__, and s__ for the ordinate label terms represent Class, Order, Family, Genus, and Species, respectively. The abscissa title has been revised from LDA to "-log10(LDA score) in LEfSe". In LEfSe analysis, the LDA score is used to measure the discriminant effect size for group differentiation, with a higher LDA score denoting a greater contribution

to intergroup differences. We have also attached the corresponding references (Segata et al., 2011; https://doi.org/10.1186/gb-2011-12-6-r60).The LDA score of *Bacillus* was 4.78, the highest among all genera in the PST group. Therefore, it can be inferred that *Bacillus* contributes the most to distinguishing the PST group from other groups and acts as a significant biomarker for the PST group. We have also added more detailed information to the figure legends.

(2) I am not sure what kind of information is conveyed in Fig 2f with no indication of what IncMSE represents.

Response to comment: We appreciate your valuable suggestions. The abscissa title of Fig. 2f has been revised from IncMSE to "Increase in Mean Squared Error". This parameter denotes the percentage increase in the model's Mean Squared Error (MSE) when a given variable is permuted, with a higher value reflecting a greater importance of the variable. The genus *Bacillus* exerted the most prominent effect on the increase in the model's MSE, reaching 6.01%. This demonstrates a significant contribution of this variable to the model and infers its high importance in the microbiome data.

(3) Same for Fig. 3e, information is not sufficiently provided for me to understand the dots. If the authors are using the same color scheme as in Fig 3a, please be specific. Consider highlighting some non-Bacillus strains that were shown to be highly enriched in the PST treatment.

Response to comment: We appreciate your valuable suggestions. The same color-coded legend is adopted in Fig. 3a and Figs. 3e-f, meaning that an identical color represents the same genus in both Fig. 3a and Figs. 3e-f. For instance, pink denotes *Bacillus* and dark blue denotes Pseudolabrys across all these panels. As indicated by the microbial bar plot in Fig. 2b, in addition to *Bacillus*, *Lactobacillus*, and *Streptococcus* are also enriched in the PST group. These two genera are represented by red and orange, respectively, in Fig. 3a, and likewise, they are highlighted with the same corresponding colors in Figs. 3e-f. We have also added more detailed information to the figure legends.

(4) For Fig. 6e and f. What are the dots, what the colors represent, how can we

understand the scale (0-100). It is not clear to me how this plot gives rise to a conclusion that microbial compositions between the strain Sy2-11and 10-HCA treatment groups are similar. I do not see particularly strong enrichment of dots along the axes. Please represent the data in another format that is more intuitive.

Response to comment: We appreciate your valuable suggestions, and we sincerely apologize for the confusion caused by the unclear legend information in Fig. 6e. We have revised the legend of Fig. 6e, where grey, green, red and purple now represent different relative abundances of ASVs, corresponding to < 0.1%, 0.1% - < 0.5%, 0.5% - 1% and > 0.1%, respectively. For ASVs with a relative abundance above 1%, their sizes are additionally mapped to their abundance values. In addition, we have added arrows to the coordinate axes of the ternary plot: the direction of each arrow corresponds to the respective treatment group, and the axis scales represent the relative abundances of ASVs. Furthermore, a red dashed auxiliary line has been included; points on this line indicate that the corresponding ASVs have identical relative abundances in the HCA and PST groups (i.e., HCA: PST = 1:1). As shown in the legend, ASVs with a relative abundance greater than 0.1% are relatively concentrated on both sides of this auxiliary line. Together with the results presented in Figs. 6c-d, these observations collectively demonstrate that the HCA and PST groups have more similar microbial compositions.

(5) Fig 6f appears redundant.

Response to comment: We appreciate your valuable suggestions. Fig. 6d shows the distribution of the top 20 genera with the highest abundances across the three groups, and a preliminary observation reveals that the genus abundances in the PST and HCA groups are more similar. Furthermore, Fig. 6e presents an analysis of all ASVs, and the overall results are consistent with our expectations. Fig. 6f is intended to illustrate that *Bacillus* is a microbe significantly enriched in both the HCA and PST groups; for more intuitive visualization, we have replaced the original plot with a scatter plot. In addition, we performed a linear fitting analysis for all genera at the genus level, with the fitting equation having a slope of 0.95 and a coefficient of determination (R^2) of 0.81. This indicates that the HCA: PST abundance ratio is indeed close to 1:1, and the

fitting model has a high rate of explaining the observed variation. These results statistically confirm once again that the HCA and PST groups have highly similar microbial compositions, and that *Bacillus* is the most significantly enriched microbe in both groups.

Point 10: 10. The materials and methods could benefit from some clarification.
(1) No information provided regarding the culturing condition of Sy2-11.

Response to comment: We thank the reviewer for the suggestions. We have added detailed information on strain Sy2-11, including its source, isolation details, cultural, physiological, and biochemical characteristics, as well as genomic data, to the methods section of the manuscript. For details, please refer to lines 564-577.

(2) What kind of hydroponic system and what media are used in the cultivation.

Response to comment: We appreciate the reviewer's suggestions. For root exudate collection, a hydroponic split-root system was utilized. During the initial rooting stage, banana plantlets were cultured in sterile Hoagland's nutrient solution. In contrast, a sterile water culture system was employed during the subsequent bacterial inoculation and root exudate collection phases.

Point 11: 11. Have you checked for plant root colonization after three days of spore inoculation [Ln621] for the collection of metabolites?

Response to comment: We greatly appreciate your valuable insight regarding the efficient colonization of strain Sy2-11 on banana roots. To determine the colonization of *Streptomyces* on banana plants, a sterile environment group was established. The colonization of *Streptomyces* on the surface of banana roots was observed using a scanning electron microscope (SEM, TM4000Plus, Hitachi, Tokyo, Japan) at 3 dpi. The results demonstrated in the axenic hydroponic system used for root exudate collection, strain Sy2-11 could effectively colonize banana roots. Furthermore, we have added relevant content to the Methods and Supplementary Materials (**Supplementary Fig. 22**), as detailed in lines 688-691.

Point 12: Have you checked for potential contamination between the left and right

chamber of the split root system? How do you make sure the metabolites are coming from plants or from microbes too?

Response to comment: We thank the reviewer for the suggestions. The banana plantlets used in the experiments were tissue-cultured virus-free plantlets, and both the split-root system and plantlet cultivation experiments were conducted under axenic conditions. Sterile water was added to the collection chambers for root exudate collection. We also performed YE agar plate assays on the sterile water from the left chambers in both the pre-experiment and the formal experiment to ensure that the root exudate collection system was free from contamination. The results confirmed that the root exudate collection system was free of contamination. We have also added this information to the methods and supplementary materials sections (Supplementary Fig. 23). Please refer to lines 698-701.

Apr-16-2026

Dear Reviewers:

We highly appreciate your favorable assessment of our work and are happy to submit a revised version of our manuscript. According to Reviewers' suggestions, we provide a point-by-point explanation of the changes and our response to your comments. The amendments are highlighted in red in the manuscript.

Response to Reviewer #1:

The manuscript has been improved significantly. However, several issues remain to be addressed, including inconsistencies between the data and their descriptions, overstatements that extend beyond the evidence presented, and a number of minor points.

Point 1: Inconsistencies between data and description: Lines 167-177. The description of the co-occurrence network results is not fully supported by the data presented in the figures.

First, regarding the experimental design for assessing the effect of Sy2-11 inoculation: To evaluate its impact on the rhizosphere microbiome, a comparison between the PST and CTL groups is necessary. To evaluate its impact on the bulk soil microbiome, a comparison between the SST and IS groups would be required.

Second, the authors state: "the average number of edges per node was similar between the strain Sy2-11 treated and SST groups, both of which were lower than the CTL group (Fig. 3d). "However, based on Fig. 3d, it is difficult to visually ascertain whether the PST (presumably Sy2-11 treated) group is more similar to the SST group or to the CTL group. Statistical significance markers should be clearly presented to support this claim.

Subsequently, the authors conclude that "pathogen pressure disrupts microbiome structure, reducing network complexity and stability." The provided data do not sufficiently support this conclusion. Contrary to the authors' claim that Sy2-11 inoculation "enhances network complexity and stability," the data in Fig. 3d suggest that inoculation may have reduced network complexity (as indicated by the lower

average edges per node) compared to the healthy CTL group. Furthermore, claims of enhanced stability require support from dedicated stability metrics, such as Robustness, Vulnerability, Fragility, Compositional stability, the ratio of negative to positive correlations, or cohesion, rather than simple node and edge counts. A more rigorous assessment of stability would ideally involve time-series data.

Finally, the authors claim: "The co-occurrence networks further indicated higher proportions of positive edges and modularity in the strain Sy2-11 treated group networks compared to the CTL and SST groups (Fig. 3a)." This cannot be deduced from the network visualization in Fig. 3a alone. Conversely, the data presented in Fig. 3c suggest that the SST group may have a higher proportion of positive correlations. Quantitative results for the proportion of positive edges and modularity index should be provided in a separate panel to substantiate this claim.

Response to comment: Thank you for your careful review of our article and for providing valuable feedback. We have added the network diagram for IS in the manuscript and revised Fig. 3 accordingly. In addition, we have substantially revised the section on network analysis as follow:

The results revealed that the IS network comprised multiple highly clustered core modules, characterized by dense within-module connectivity but very few between-module links. The numbers of nodes and edges were substantially higher than those in the CTL, PST, and SST groups (**Supplementary Fig. 10**). In IS and SST groups, *Muribaculaceae* was the dominant core genus and occupied most of the major modules within the network. By contrast, the PST and CTL groups exhibited significantly higher numbers of degrees, nodes, and edges than the SST group, indicating that taxa in the CTL and PST groups formed more interaction links (**Fig. 3a-c**). Compared with the CTL group, the PST group contained more nodes but relatively fewer edges, along with a higher proportion of positive interactions (**Fig. 3a-d**). These network matrices suggest that, in the presence of banana seedlings, the pathogen established a relatively stable pathogenic microbial community, whereas the introduction of strain Sy2-11 disrupted this community, reduced network complexity and stability, and thereby enhanced soil disease resistance.

Please refer to lines 188-201 for details in the revised manuscript.

Point 2: Overstatements / Claims extending beyond the presented evidence.

(1). Lines 27-32 (Abstract): Certain claims in the abstract may extend beyond the scope strongly supported by the data. The description of the mechanism "whereby Streptomyces, acting as a novel and beneficial elicitor, releases sesquiterpene signals to trigger 10-HCA secretion in banana plants" is presented as a new finding. However, stating that this constitutes a "novel strategy" might be an overstatement, as the general strategy of using microbial inoculation to indirectly manipulate the rhizosphere microbiome for disease suppression has been reported previously. It is recommended to temper this claim, for instance, by describing it as a "potential" or "promising" novel strategy.

(2). A similar adjustment is suggested for the statement in Lines 468-469.

(3). Line 561: The phrase "microbiome-oriented biological breeding" is puzzling in this context, as the present study does not involve work related to plant genotype or breeding programs. Its inclusion here is unclear and potentially misleading.

Response to comment: Thank you for your careful review and constructive comments on our manuscript.

(1) According to the reviewer's suggestions, we have revised the last sentence of the abstract as follows: “These findings provide a promising strategy for rhizosphere micro-ecological regulation and sustainable soil-borne disease control, with significant potential for advancing sustainable agriculture.”

(2) The statement in Lines 475-476 has been revised as follows: “This study, therefore, provides a promising strategy for the efficient utilization of *Streptomyces*.”

(3) In accordance with the reviewer's suggestion, we have revised this part of the manuscript in line 561, as follow: This study will provide theoretical support and effective approaches for “rhizosphere microecological regulation”, and it will play a more active role in sustainable agriculture. Please refer to Lines 545-547 for details.

Point 3: Minor points

(1). To enhance readability, it is suggested to add a brief introductory description of strain Sy2-11 at the beginning of the Results section (around Line 94).

Response to comment: We greatly appreciate your careful review of our manuscript and the valuable comments provided. We fully agree with your opinion. A brief introduction of strain Sy2-11 was added at the beginning of the Results section as follow: In our previous study, a novel strain, *S. yongxingensis* sp. nov. 2-11, was newly identified from the soft coral *Menella woodin*[10]. The type strain Sy2-11^T (=GDMCC 4.213^T=JCM 34965^T) is a gram-positive, aerobic, and non-motile actinobacteria forming branched substrate and aerial mycelia, which differentiate into short and spiral spore chains. Strain Sy2-11 was highly effective in controlling BFW. Please refer to lines 94-98 for details in the revised manuscript.

(2). Regarding formatting:

In Figure 2, the "P" in P-values should be italicized.

All microbial genus names in the text and figures should be formatted in italics.

Response to comment: We thank the reviewer for pointing out the formatting errors. We sincerely apologize for these mistakes and have now corrected all of them, including Figures 1, 2, 3, 4, 6, Supplementary Fig. 1, Supplementary Fig. 6, and Supplementary Fig. 7.

(3). Data specification check:

Line 411: Please verify and specify whether the values reported are log10FC or log2FC.

Response to comment: Thank you for pointing out the error. The authors are very sorry for this mistake and have corrected it. It’s log_2FC.

(4). References require the following corrections:

Line 931: "N. Phytol." should be corrected to "New Phytol."

Line 966: "N. Phytol." should be corrected to "New Phytol."

Line 970: "Arabidopsis thaliana" should be italicized.

Response to comment: Thank you for pointing out the error. The authors are very sorry for this mistake and have corrected it.

Response to Reviewer #2:

The authors conducted series of experiments and have nicely addressed all comments in the revised manuscript. The discussion section is overly lengthy. Discussions can be more focused and concise. Please revise it accordingly.

Response to comment: We greatly appreciate your careful review of our manuscript and the valuable comments provided. According to the reviewer's suggestions, we have revised and simplified the Discussion section accordingly.

Response to Reviewer #3:

The authors have addressed most of my concerns/suggestions in the revision. However, the manuscript still required additional clarification, specifically on the colonization competence of Sy2-11 strain.

If I understood correctly, Sy2-11 was isolated from marine soft coral collected from 15-20m depth of the ocean. It is surprising that this marine strain can colonize and survive on banana root. The authors have performed multiple experiments but they are not entirely conclusive.

Point 1: The use of 16S-based sequencing failed to distinguish Sy2-11 from strains sharing the same partial 16S sequences as reflected in the detection of the same ASV in non-inoculated control.

Response to comment: We greatly appreciate your careful review of our manuscript and the valuable comments provided. It is well known that the 16S rRNA of *Streptomyces* lacks species-specific sequences. It is difficult to distinguish *Streptomyces* species based on 16S rRNA, which can only be used to differentiate genera. Furthermore, the amplicon sequences corresponding to ASVs are only 400 bp in length, which makes it impossible to identify specific *Streptomyces* species. we generated a GFP-labeled strain Sy2-11 and observed its colonization of banana roots using confocal laser scanning microscopy (CLSM) (**Supplementary Fig. 2**). These observations demonstrated that strain Sy2-11 can effectively colonize banana roots.

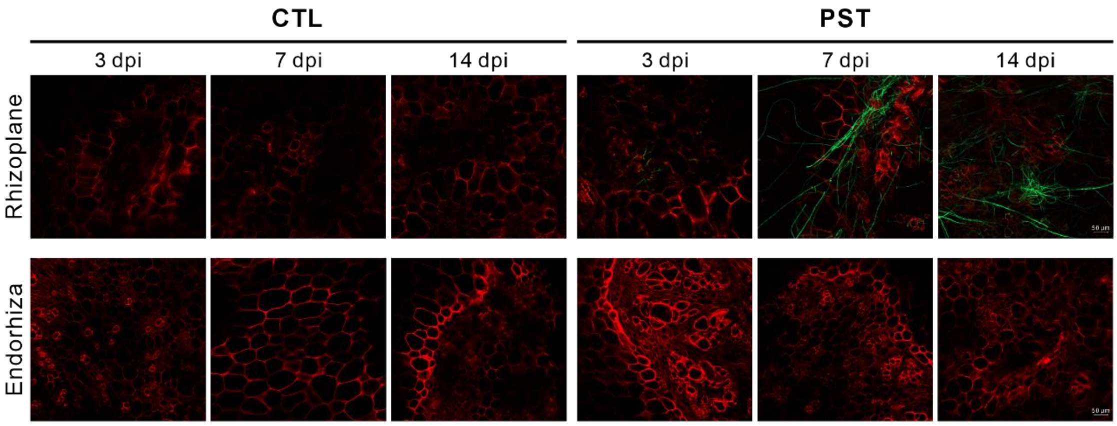

Supplementary Fig. 2. Colonization of banana rhizoplane and endorhiza by strain Sy2-11. Roots from uninoculated plants (CTL) and plants inoculated with strain Sy2-11 (PST) were

observed using confocal laser scanning microscopy (CLSM) at 3, 7, and 14 days post-inoculation (dpi).

Point 2: What are those thread-like structures represent in Supp Fig 2. Are you suggesting that those are live bacteria? What is the time point for observation (i.e. how many days after inoculation)? If only early time point is taken, it may not reflect true colonization, see below.

Response to comment: Thank you for your careful review and constructive comments on our manuscript. To determine the ability of strain Sy2-11 to colonize the banana rhizosphere soil, rhizoplane, and endorhiza, we first labeled strain Sy2-11 with a GFP marker to investigate its competence to colonize banana roots using confocal laser scanning microscopy (CLSM). Abundant GFP-Sy2-11 mycelia were observed on the root surface at 3, 7, and 14 dpi (**Supplementary Fig. 2**). In contrast, no GFP-Sy2-11 fluorescence was detected inside root tissues, indicating that the strain colonizes the rhizosphere effectively, and has the potential to establish long-term associations with banana plants. Second, we generated an ampicillin-resistant derivative of strain Sy2-11 and quantified its colonization density in the banana rhizosphere soil, rhizoplane, and endorhiza. Results showed that the colonization counts of strain Sy2-11 in rhizosphere soil were 5.36×10^5, 3.08×10^5, and 2.55×10^4 CFU/g soil at 3, 7, and 14 dpi, respectively. In contrast, the colonization count of strain Sy2-11 in the rhizoplane was 1.68×10^3, 4.30×10^3, and 5.53×10^3 CFU/g soil at 3, 7, and 14 dpi, respectively. However, no strain Sy2-11 was detected within banana root tissues (**Supplementary Fig. 3**). For details, please refer to lines 111-120 of the manuscript.

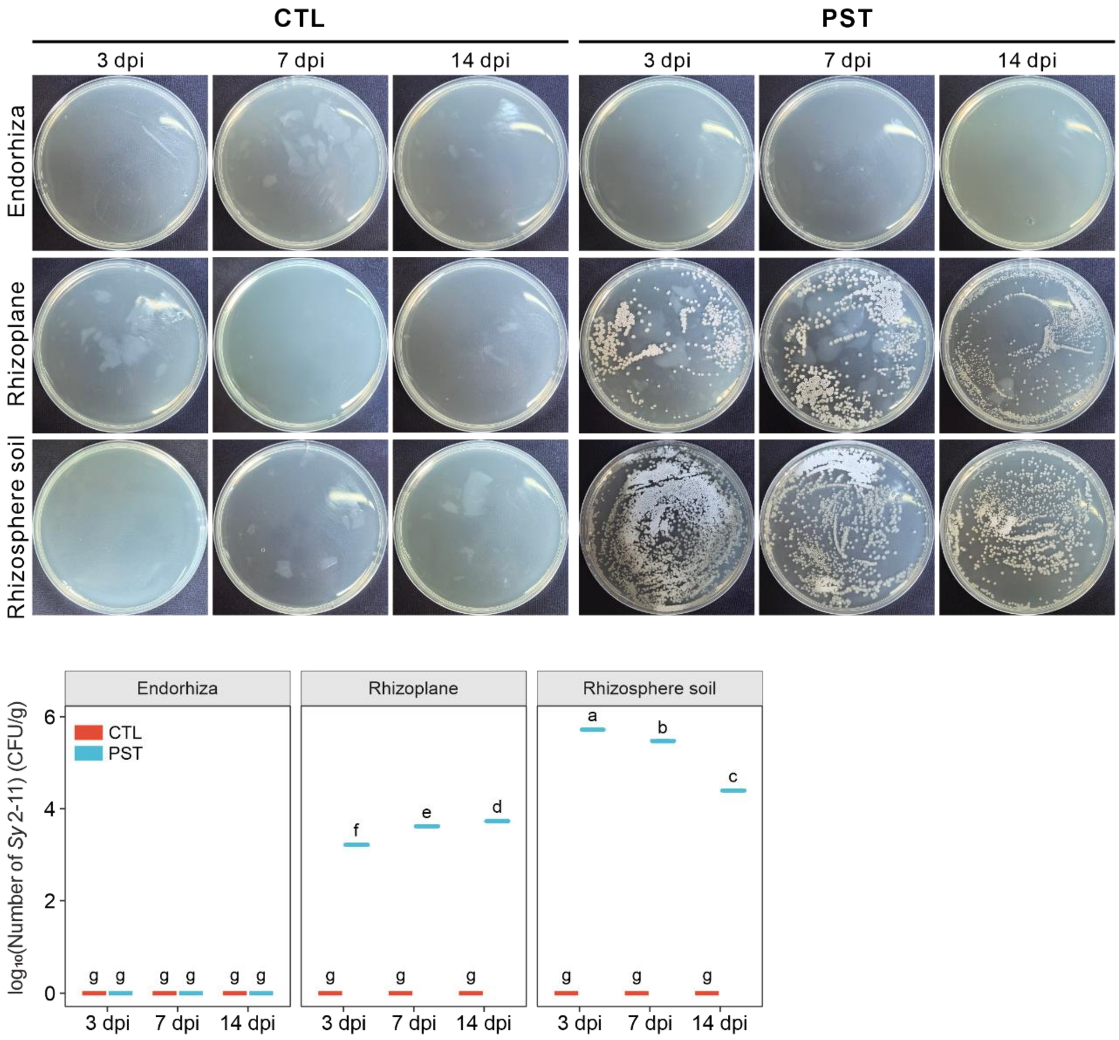

Supplementary Fig. 3. Colonization dynamics of strain Sy2-11 to colonize the banana rhizosphere soil, rhizoplane, and endorhiza. Soils and roots from uninoculated plants (CTL) and plants inoculated with strain Sy2-11 (PST) were used to quantify their population at 3, 7, and 14 days post-inoculation (dpi).

Point 3: Fig R3 needed to be strengthened. First, information related to the input inoculum, method of inoculation, time of incubation and the washing condition were missing. Second, the time of co-incubation should be long enough to match the experimental design to have a protective effect. Third, if the experiment was performed using high input and short time of incubation, one cannot exclude the possibility that the strains were thriving on residual nutrients without persistent colonization. For instance, if you repeat the experiment using dead plant materials, maybe you can see the same pattern of colonization.

Response to comment: We greatly appreciate the reviewer’s valuable comments.

Following your suggestion, we have provided detailed experimental procedures in the Methods section of the manuscript and added the corresponding observations in the **Results section**. For details, please refer to lines 111-120 and 584-592 of the manuscript.

Point 4: As stated before, direct plant root colonization was not demonstrated. Provided that Streptomyces strains survived under harsh condition (for example upon changes from marine to soil), it is possible that Sy2-11 were germinating as a survival strategy and produced metabolites (including antibiotics) that shaped microbial composition. SST is not a perfect control as explained before. Without a 10-HCA biosynthesis mutant, one cannot rule out this possibility. Regardless, I do not think it will affect the major conclusions but the authors should address these clearly in the result section based on evidence rather than speculation. The model has to be revised accordingly.

Response to comment: We greatly appreciate your careful review of our manuscript and the valuable comments provided. We fully agree with your opinion. To determine the ability of strain Sy2-11 to colonize the banana rhizosphere soil, rhizoplane, and endorhiza, we first labeled strain Sy2-11 with a GFP marker to investigate its competence to colonize banana roots using confocal laser scanning microscopy (CLSM). Second, we generated an ampicillin-resistant derivative of strain Sy2-11 and quantified its colonization density in the banana rhizosphere and roots. Results showed that strain Sy2-11 can effectively colonize banana roots.

To clarify the model by which strain Sy2-11 enriches antagonistic *Bacillus* by inducing banana root secretion of 10-HCA, we initially considered two experimental approaches. First, we considered comparing the rhizosphere microbiomes of wild-type banana plants and banana mutants defective in 10-HCA biosynthesis, in order to directly assess the impact of 10-HCA on rhizosphere microbial assembly and *Bacillus* enrichment. Second, we considered comparing the rhizosphere microbiomes of plants treated with exogenous 10-HCA and plants inoculated with strain Sy2-11, to further elucidate the role of 10-HCA in microbiome modulation and *Bacillus*

recruitment. However, the biosynthetic pathway of 10-HCA in banana roots is complex. Disruption of genes involved in its biosynthesis module would inevitably trigger a cascade of linked reactions, leading to the disappearance of some original root exudates and the emergence of new ones. This would make it difficult to distinguish whether changes in the rhizosphere microbiome are directly caused by the absence of 10-HCA or indirectly by the newly produced exudates. Therefore, as shown in **Fig. 6**, we adopted a more conservative strategy and investigated rhizosphere microbiome changes mediated by exogenous 10-HCA application, with the aim of clarifying whether the enrichment of antagonistic *Bacillus* is attributable to 10-HCA secretion. Results showed that banana seedlings in both the strain Sy2-11 and 10-HCA treatments remained healthy compared with the control group (Fig. 6a-b). Microbiota community analysis showed that no significant differences in α-diversity indices between the strain Sy2-11 treatment and 10-HCA treatment, whereas both treatments differed significantly from the control group. The microbial community composition was highly similar between strain Sy2-11 and 10-HCA treatments. Among the differentially abundant taxa, the genus *Bacillus* was significantly enriched in both the strain Sy2-11 and 10-HCA treatments compared with the control group (Fig. 6c-f). These results further confirm that strain Sy2-11 induces banana roots to secrete 10-HCA, which in turn recruits disease-suppressive *Bacillus* to participate in the disease resistance process.

Furthermore, to verify the model in which strain Sy2-11 induces banana roots to secrete 10-HCA, thereby enriching antagonistic *Bacillus*, we conducted a soil-based cultivation experiment using a split-root system shown in **Fig. R1a**. The experimental design included two treatments: (1) CTL: plants treated with sterilized water; (2) PST: plants inoculated with strain Sy2-11 (1×10^6 CFU/g soil). Rhizosphere soil was collected from the left compartment for 16S rRNA gene sequencing. The results showed that *Bacillus* was significantly enriched in the left-compartment soil of the PST group, indicating that the assembly of the disease-suppressive microbiome is mediated by banana root exudates.

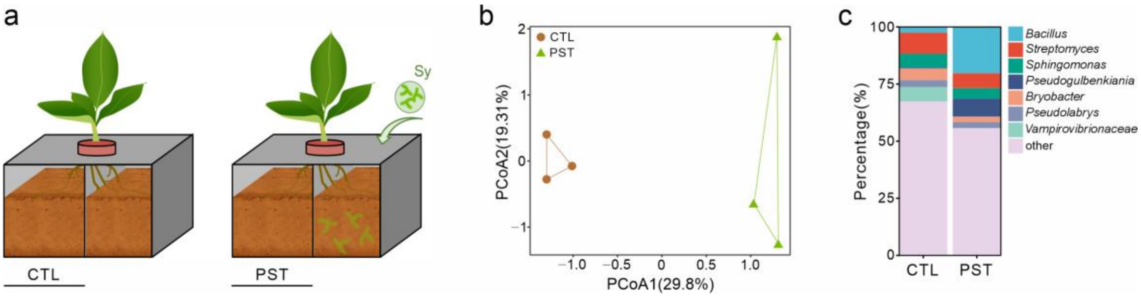

Fig. R1. *Bacillus* as the core microbiota recruited by strain Sy2-11-induced banana roots. (**a**) Flowchart of the experimental design for a split-root system. (**b**) The impact of strain Sy2-11 on the β-diversity of bacterial communities in the banana rhizosphere. (**c**) Bar plot displaying the relative abundance of ASVs at the genus level across the CTL and PST experimental groups, highlighting the top seven genera.

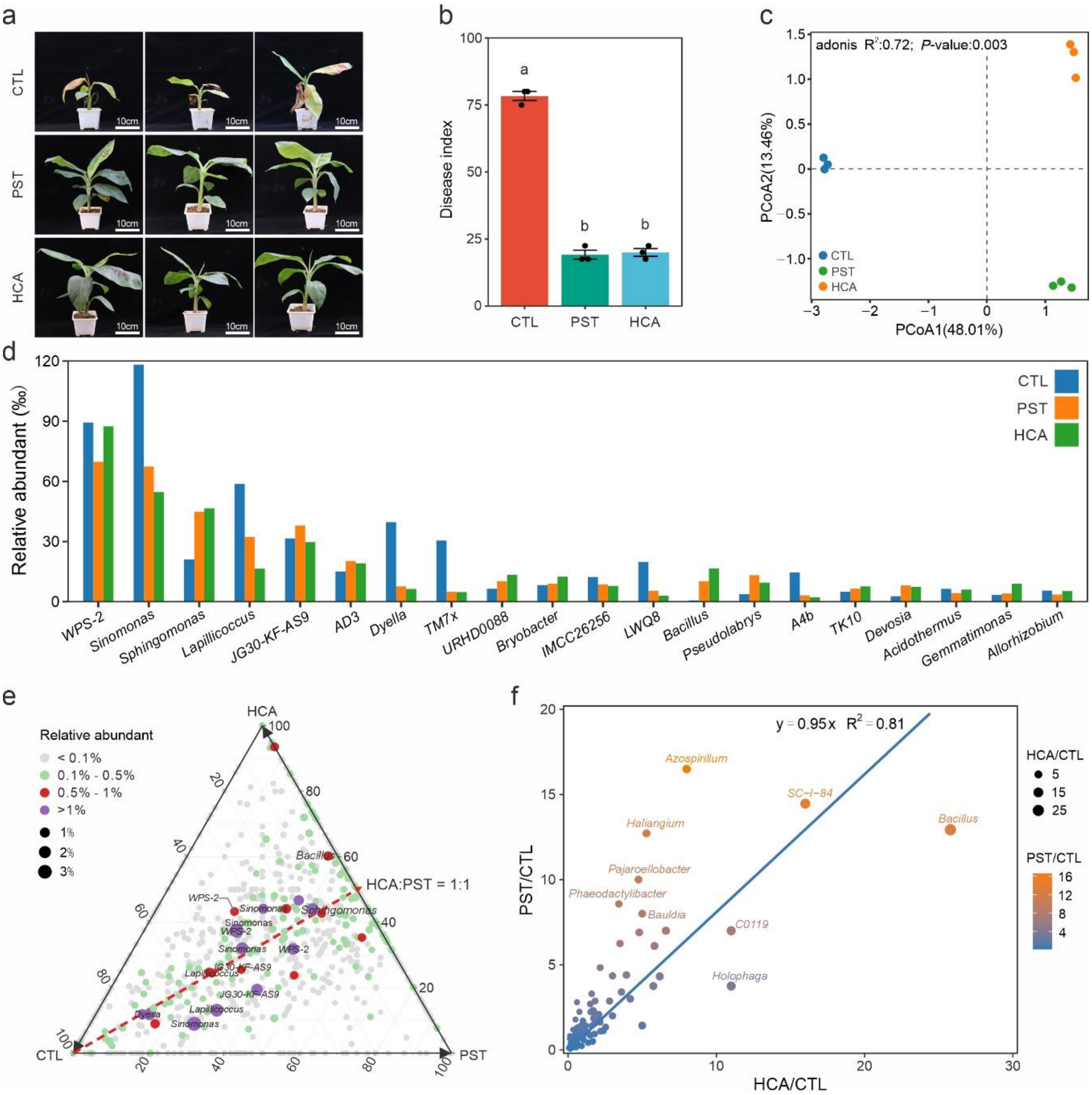

Fig. 6. Impact of 10-HCA on wilt disease incidence and rhizosphere microbiome of banana plants. (**a**) phenotypic comparison of banana seedlings following treatment with strain Sy2-11 and 10-HCA. Seedings in the 10-HCA and PST groups showed healthy growth, while those in the CTL group exhibited obvious symptoms of *Fusarium* wilt disease. Images were taken at 35 dpi. (**b**)

Disease index comparison among the CTL, PST, and 10-HCA groups. Statistical significance was assessed using LSD multiple comparison tests, with different letters indicating significant differences ($P < 0.05$). (**c**) Bacterial *β*-diversity in the rhizosphere soils was analyzed using principal coordinate analysis (PCoA). The symbol colors in (c) correspond to the legend provided in (d). The R^2 and *P*-value were derived from a PERMANOVA (permutational multivariate analysis of variance) test. (**d**) Bar chart illustrating the relative abundance of the top 20 genera. (**e**) Ternary plot revealing the overall distribution similarity between the PST group and the 10-HCA group. Colors (grey, green, red, and purple) and point sizes indicate different levels of ASV relative abundance, as explained in the legend. The red dashed line represents an HCA:PST ratio of 1:1. Several highly abundant genera are labeled with text. (**f**) Genus-level fold change analysis relative to the CTL group revealed a strong similarity in microbial profiles between the HCA and PST treatments. Besides, *Bacillus* was identified as the most significantly enriched genus in both the PST and 10-HCA groups compared to the CTL group. CTL: control treatment consisted of plants treated with sterilized water; PST: plants inoculated with strain Sy2-11; HCA: plants inoculated with 10-HCA.

Point 5: Based on reference 61, Sy2-11 was growing on M1, M2, M3, and M4 agar media. Please specify the composition of the media and clarify whether this medium carried high amount of salt or specific nutrients to mimic the marine environment.

Response to comment: Thank you for your careful review of our article and for providing valuable feedback. In reference 61, strain Sy2-11 was isolated from a soft coral by a serial dilution method on M1, M2, M3, and M4 agar media. The compositions of M1-M4 are provided in **Table R1**. Subsequently, to investigate the cultural characteristics of strain Sy2-11, the purified strain Sy2-11 was cultivated on eight different media, including ISP2, ISP3, ISP4, ISP5, ISP6, ISP7, and PDA. The results showed that strain Sy2-11 grew vigorously on all eight culture media. The compositions of the eight media used for the cultural characterization are shown in **Table R2**. We later used PDA medium to determine the antifungal activity of strain Sy2-11, and employed FM1 fermentation medium (Soluble starch 10 g, Yeast extract 4 g, Peptone 2 g, Glucose 20 g, K_2HPO_4 0.5 g, $CaCO_3$ 2 g, $MgSO_4$ 0.5 g, $(NH_4)_2SO_4$ 0.5 g, H_2O 1000 mL, natural pH) for fermentation and metabolite collection.

During the initial isolation of the strain, half-strength sea salt was added to the medium to mimic its original growth environment. Subsequently, the cultural characterization showed that strain Sy2-11 could grow well on all characterization media without sea salt. Therefore, all subsequent experiments were carried out using

regular media without sea salt. In general, strain Sy2-11 was cultured in YE liquid medium (Yeast extract 4 g, Malt extract 10 g, Glucose 4 g, H_2O 1000 mL, pH 7.3) to prepare the seed culture for subsequent inoculation. Pot experiments further demonstrated that strain Sy2-11 was able to colonize the banana rhizosphere soil and root surface effectively.

Table R1 Compositions and concentrations of the isolation media

Media	Compositions (g/L)
M1	Glucose 4 g, Yeast extract 4 g, Malt extract 5 g, Sea salt 15 g.
M2	Soluble starch 10 g, Peptone 1 g, Yeast extract 0.5 g, $MgSO_4$ 1 g, K_2HPO_4 1 g, $(NH_4)_2SO_4$ 2 g, $CaCO_3$ 2 g, Sea salt 15 g.
M3	Chitin 4 g, $CaCO_3$ 2 g, K_2HPO_4 0.3 g, $MgSO_4$ 0.5 g, KH_2PO_4 0.7 g, $FeSO_4$ 0.01 g, $MnCl_2$ 0.01 g, Sea salt 15 g.
M4	Fucose 5 g, Proline 1 g, $MgSO_4$ 1 g, K_2HPO_4 1 g, $(NH_4)_2SO_4$ 1 g, $CaCO_3$ 2 g, Sea salt 15 g.
Considerations	All media contained 18 g/L agar as a solidifying agent and were adjusted to pH 7.2. To inhibit the growth of fungi, 50 mg/L streptomycin and 50 mg/L $K_2Cr_2O_7$ were added to the basic media.

Table R2 Compositions of the media used for cultural characterization

Name	Compositions (g/L)
Yeast malt extract agar （ISP2/YE）	Yeast extract 4 g, Malt extract 10 g, Glucose 4 g, H_2O 1000 mL, Agar 20 g, pH 7.3.
Oatmeal agar（ISP3）	Oatmeal 20 g, Trace salt solution 1 mL, Agar 20 g, pH 7.2.
Inorganic salt starch agar （ISP4）	Soluble starch 10 g, K_2HPO_4 1 g, NaCl 1 g, $MgSO_4 \cdot 7H_2O$ 1 g, $(NH_4)_2SO_4$ 2 g, $CaCO_3$ 2 g, Trace salt solution 1 mL, H_2O 1000 mL, Agar 20 g, pH 7.0-7.4.
Glycerol asparagine agar （ISP5）	Asparagine 1 g, Glycerol 10 g, K_2HPO_4 1 g, Trace salt solution 1 mL, H_2O 1000 mL, Agar 20 g, pH 7.0-7.4.
Peptone yeast extract agar	Peptone 15 g, Proteose peptone 5 g, Ammonium ferric citrate 0.5 g, K_2HPO_4

（ISP6）	1 g, Sodium thiosulfate 0.08 g, Yeast extract 1 g, H_2O 1000 mL, Agar 20 g, pH 7.0-7.4.
Tyrosine agar（ISP7）	Glycerol 15 g, tyrosine 0.5 g, Asparagine 1 g, K_2HPO_4 0.5 g, $MgSO_4·7H_2O$ 0.5 g, NaCl 0.5 g, $FeSO_4·7H_2O$ 0.01 g, Trace salt solution 1 mL, DDH_2O 1000 mL, Agar 20 g, pH 7.2-7.4.
PDA	Potato 200 g, Glucose 20 g, Agar 17-20 g, ddH_2O 1000 mL, pH 7.0.
Trace salt solution	$FeSO_4·7H_2O$ 0.1 g, $ZnSO_4·7H_2O$ 0.1 g, $MnCl_2·4H_2O$ 0.1 g, H_2O 100 mL.

Point 6: New data should be integrated as part of the manuscript. Specifically, Fig R4 related to the dosage dependent effect of Sy2-11 on banana Fusarium wilt disease severity should be included as a main figure. Note that the use of strain 5-6 and H7 with comparable input (10^6 cfu/g) also reduced disease incidence, suggesting that inoculation of Streptomyces strains with high concentration have some general protective effect, supporting the suspicion that 10-HCA independent mechanism contributed significantly to protection. Please discuss accordingly. Same for the data related to the variations in protective effect in the presence or absence of indigenous microbiota.

Response to comment: Thank you for your insightful comments. We have supplemented the manuscript with the relevant methods and results. For the methods section, please refer to lines 578-583. For the results section, please refer to lines 143-151. In addition, we have further discussed this content and added it to the Discussion section, as detailed in lines 402-413.

May-10-2026

Dear Reviewers:

We highly appreciate your favorable assessment of our work and are happy to submit a revised version of our manuscript. According to Reviewers' suggestions, we provide a point-by-point explanation of the changes and our response to your comments. The amendments are highlighted in red in the manuscript.

Response to Reviewer #3:

The authors have addressed all my concerns especially on the colonization competence of Sy2-11 on banana. The model is strengthened and well-supported. I still find some textual edits needed but I believe it can be addressed during the proofing process without another round of revision.

ln 118: trial instead of trail

ln 150: if these streptomyces strains were not sequenced, I cannot tell the whether these strains are similar to Sy2-11. It is better to remove the word 'homologous' if the authors are not sure.

ln 411-412. If the authors insist that these data cannot be included in the manuscript. Please remove the statement 'Our study showed that the direct antagonistic effect of strain Sy2-11 against Foc TR4 was completely lost in sterilized soil'.

Response to comment: Thank you for your careful review and constructive comments on our manuscript.

(1) Thank you for pointing out the error, we have already changed "trail" to "trial" in line 118.

(2) According to the reviewer's suggestions, we have already deleted the word "homologous" in line 150. Additionally, we have also removed the statement: "Our study showed that the direct antagonistic effect of strain Sy2-11 against *Foc* TR4 was completely lost in sterilized soil.".
